# Supplementary material for: Associations of smoking and alcohol consumption with healthy ageing: a systematic review and meta-analysis of longitudinal studies
Source: BMJ Open. 2018 Apr 17;8(4):e019540. doi: 10.1136/bmjopen-2017-019540 (PMC5905752; doi:10.1136/bmjopen-2017-019540)
Supplement: Supplementary file 1 [file bmjopen-2017-019540supp001.pdf]

MOOSE Checklist for Meta-analyses of Observational Studies

Item No	Recommendation	Reported on Page No
Reporting of background should include		
1	Problem definition	To address the need for more accurate estimates of the relationship between smoking and healthy ageing and alcohol consumption and healthy ageing (p. 6)
2	Hypothesis statement	In systematic reviews focusing on elderly people, smoking is associated with premature mortality and an increased incidence of Alzheimer's disease. Studies showed that light to moderate alcohol consumption is associated with a decrease in dementia and cognitive incidence, as well as with a reduced risk for cardiovascular disease mortality, incident coronary heart disease, incident stroke, stroke mortality and all-cause mortality(p. 5)
3	Description of study outcome(s)	The primary outcome of this review was health status measured by healthy ageing, and any other term related to it (e.g. successful ageing, active ageing, healthy survival etc.) (p. 7)
4	Type of exposure or intervention used	Eligible studies had to meet the following criteria: i. being published in an electronic journal article; ii. constitute an original peer-reviewed longitudinal study; and iii. report any kind of longitudinal association between smoking and/or alcohol consumption and healthy ageing (p. 7)
5	Type of study designs used	longitudinal study (p. 7)
6	Study population	Studies that included cohorts of individuals who were institutionalised or hospitalised and animal studies were also excluded (p. 7)
Reporting of search strategy should include		
7	Qualifications of searchers (eg, librarians and investigators)	Credentials are indicated in the title page
8	Search strategy, including time period included in the synthesis and key words	Search strategy and selection criteria (p.6-7) and Appendix 3.docx
9	Effort to include all available studies, including contact with authors	In case that full text could not be retrieved, the corresponding author of the paper was contacted (p. 7).<br>Other relevant systematic reviews of healthy ageing and reference lists of the eligible studies were also searched (p.7)
10	Databases and registries searched	MEDLINE (PubMed interface), EMBASE (OVID interface), Psychinfo (OVID interface) and CENTRAL (Cochrane Central Register of Controlled Trials) (p. 6-7)
11	Search software used, name and version, including special features used (eg, explosion)	EndNote (ENDNOTE X7, Thomson Reuters), (p. 7)
12	Use of hand searching (eg, reference lists of obtained articles)	Other relevant systematic reviews of healthy ageing and reference lists of the eligible studies have also been searched (p. 7)
13	List of citations located and those excluded, including justification	Details of the literature search are outlined in the PRISMA flow-chart: Figure 1. The citation list is available upon request.
14	Method of addressing articles published in languages other than English	Acknowledgements (p. 20)
15	Method of handling abstracts and unpublished studies	exclusion of papers that were abstracts, conference papers, cross-sectional studies (p. 9-10)
16	Description of any contact with authors	we planned to contact authors for any missing data; however, this was not needed.

Reporting of methods should include		
17	Description of relevance or appropriateness of studies assembled for assessing the hypothesis to be tested	Search strategy and selection criteria paragraph (p. 6-7)
18	Rationale for the selection and coding of data (eg, sound clinical principles or convenience)	Data extraction and quality assessment paragraph (p. 8) and Table 1 and Table 2. We extracted data from each study that were relevant to the population, the exposure, the outcome and any confounder.
19	Documentation of how data were classified and coded (eg, multiple raters, blinding and interrater reliability)	Data extraction and quality assessment paragraph (p. 8).
20	Assessment of confounding (eg, comparability of cases and controls in studies where appropriate)	We extracted information regarding the different confounders that were used per study (Table 2)
21	Assessment of study quality, including blinding of quality assessors, stratification or regression on possible predictors of study results	Two investigators assessed study quality by using the QUIPS tool (Quality Assessment paragraph, p.11 and Table A2)
22	Assessment of heterogeneity	Heterogeneity was assessed by the I^2 (Data Analysis paragraph, p. 9)
23	Description of statistical methods (eg, complete description of fixed or random effects models, justification of whether the chosen models account for predictors of study results, dose-response models, or cumulative meta-analysis) in sufficient detail to be replicated	random effects meta-analysis was used (p. 9)
24	Provision of appropriate tables and graphics	We provided the PRISMA flow-chart, tables with the characteristics of studies, table with the results of meta-analysis and forest plots
Reporting of results should include		
25	Graphic summarizing individual study estimates and overall estimate	Figure 2 and Figure 3
26	Table giving descriptive information for each study included	Table 1 and Table 2
27	Results of sensitivity testing (eg, subgroup analysis)	Subgroup analysis (page 12-13)
28	Indication of statistical uncertainty of findings	95% confidence intervals were presented with all estimators, I^2 values, and results of the trim-and-fill algorithm. Table 3.

Item No	Recommendation	Reported on Page No
Reporting of discussion should include		
29	Quantitative assessment of bias (eg, publication bias)	Publication bias was assessed Egger test and the trim-and-fill algorithm (Table 3)
30	Justification for exclusion (eg, exclusion of non-English language citations)	We did not exclude any study due to language. We excluded studies for the meta-analysis because they did not report a relevant statistic to OR or because alcohol conversion to comparable metrics was not possible
31	Assessment of quality of included studies	Data extraction and quality assessment paragraph (p. 8) and Table A2.
Reporting of conclusions should include		
32	Consideration of alternative explanations for observed results	Limitations are discussed in parag. Strengths and Limitations (p. 17). The most important are: adjustment by different use of covariates, different follow-up time and attrition rate, self-reported questionnaires and inconsistency in the way smoking/alcohol have been measured among the different cohorts.
33	Generalization of the conclusions (ie, appropriate for the data presented and within the domain of the literature review)	The positive impact of smoking abstinence and smoking cessation on healthy ageing is identified in both the majority of our primary studies and in our pooled effect estimate as well (even when we adjusted for publication bias). A positive relationship between limited

		consumption of drinking could also be argued but more research is needed.
34	Guidelines for future research	Conclusion parag. (p. 19)
35	Disclosure of funding source	Funding parag. (p.20-21)

From: Stroup DF, Berlin JA, Morton SC, et al, for the Meta-analysis Of Observational Studies in Epidemiology (MOOSE) Group. Meta-analysis of Observational Studies in Epidemiology. A Proposal for Reporting. *JAMA*. 2000;283(15):2008-2012. doi: 10.1001/jama.283.15.2008.
Transcribed from the original paper within the NEUROSURGERY® Editorial Office, Atlanta, GA, United Sates. August 2012.
