# Supplementary material for: Associations of smoking and alcohol consumption with healthy ageing: a systematic review and meta-analysis of longitudinal studies
Source: BMJ Open. 2018 Apr 17;8(4):e019540. doi: 10.1136/bmjopen-2017-019540 (PMC5905752; doi:10.1136/bmjopen-2017-019540)
Supplement: Supplementary file 2 [file bmjopen-2017-019540supp002.pdf]

PROSPERO International prospective register of systematic reviews

ATHLOS: impact of physical activity, smoking and alcohol on healthy ageing throughout the lifespan: protocol for a systematic review

Christina Daskalopoulou, Matthew Prina, Martin Prince, Artemis Koukounari

Citation

Christina Daskalopoulou, Matthew Prina, Martin Prince, Artemis Koukounari. ATHLOS: impact of physical activity, smoking and alcohol on healthy ageing throughout the lifespan: protocol for a systematic review. PROSPERO 2016:CRD42016038130 Available from
http://www.crd.york.ac.uk/PROSPERO_REBRANDING/display_record.asp?ID=CRD42016038130

Review question(s)

What is the impact of physical activity, smoking and alcohol consumption across the life span on healthy ageing?

Quantification and summary of the relationship between the aforementioned lifestyle factors and healthy ageing by using a meta-analytical approach.

Searches

The literature search will include any related term or synonym of healthy ageing and text words related to physical activity, smoking and alcohol. Where possible, the latter will be developed using medical subject headings (MeSH). We will search MEDLINE (PubMed interface), EMBASE (OVID interface), PsycINFO (OVID interface), Web of Science and CENTRAL (Cochrane Central Register of Controlled Trials).

In addition, scanning of the reference lists of the included studies will take place so as to identify any relevant studies that were not included by the initial research. Finally, authors of relevant studies will be contacted in case that supplementary information is required.

Types of study to be included

This review will be restricted to original studies (peer-reviewed).

Opinion pieces, books, letters, editorials, abstracts, dissertations/thesis and/or conference proceedings will not be considered.

Condition or domain being studied

Health status measured by healthy ageing.

Participants/ population

Eligible studies will include people, who are community-based, across the whole life span. Hospitalised or institutionalised populations will not been considered. Participants being disabled or diagnosed with dementia, frailty or any chronic disease will be included in the systematic review as long as they are community-based. There will be no other restrictions, for instance gender, age, education level, occupation and/or ethnicity.

Intervention(s), exposure(s)

Associations of either physical activity, smoking and/or alcohol consumption to one of the outcomes of healthy ageing, irrespectively of the way the latter is being measured/defined.

Comparator(s)/ control

Not applicable.

Context

Community-based populations and not clinical ones.

Outcome(s)

Primary outcomes

Health status measured by healthy ageing.

Secondary outcomes

Not applicable.

Data extraction, (selection and coding)

An EndNote (ENDNOTE X7, Thomson Reuters) library will be created so as to store all the studies retrieved by the electronic databases. Using EndNote's auto-deduplication function, duplicate citations will be removed. Since auto-deduplication is thought to be only partially successful (Qi et al., 2013), the remaining duplicates will be identified by hand-searching techniques. To do this, references will be alphabetically ordered according to the first authors' names and thereafter according to their titles.

Studies from the electronic database searches will be initially determined as relevant or not by title and abstract screening. References will be characterised as "Not Eligible" and "Potentially Eligible" by screening the title and the abstract of the study.

In order to include a study in the "Potential Eligible" folder the following criteria will be applied:

Study characteristics: "potentially eligible studies" have to be published in an electronic journal article and constitute an original peer-reviewed longitudinal study. We will include studies that examined healthy ageing as an outcome and that reported on the following determinants: physical activity, smoking and/or alcohol consumption.

Study participants: to be "potentially eligible", the title or abstract of the study must clearly state that the study population is an observational study/cohort study and that people are not hospitalised or belong to a specific clinical population. Participants being disabled or diagnosed with dementia, frailty or any chronic disease will be included in the systematic review as long as they are community based. There will be no other restrictions.

Interventions: To be "potentially eligible", the title or abstract must clearly state that the study reports the associations of either physical activity, smoking and/or alcohol consumption to one of the outcomes of healthy ageing, irrespectively of the way the latter is being measured/defined.

The final inclusion will be based on full text screening. Full text studies of "potentially eligible" references will be retrieved and assessed for their final inclusion by the first reviewer and a second researcher. A random sample of those, accounting for the 5% of the total studies, will be checked by a senior epidemiologist. Once the screening process is completed, the creation of a PRISMA flow diagram will be generated so as to illustrate the study selection process.

Risk of bias (quality) assessment

The risk of bias in primary studies will be assessed by using the Quality in Prognosis Studies (QUIPS) tool (Hayden et al., 2013). Validity and bias should be assessed by considering the following 6 areas: participation, attrition, prognostic factor measurement, confounding measurement and account, outcome measurement and analysis and reporting. During the first step, the 6 aforementioned potential sources of bias will be classified as either "yes", "partly", "no" or "unsure" by the first reviewer. Each study will then be characterised with an overall risk bias rating of "high", "moderate" or "low".

This characterisation will be defined as follows: "low" risk of bias will have these studies which fulfil all or the majority of the 6 QUIPS tool domains (no source of bias should be characterised as "yes" and at most one "partly"). "Moderate" risk of bias will have the studies that fullfil some of the aforementioned criteria; and "high" risk of bias will have those that meet few or none of the criteria.

During the next step, the first reviewer and the senior epidemiologist will judge if any of the "high" and "moderate" level studies present fatal flaws or considerable amount of bias and hence, in order to avoid reaching any misleading conclusion, it would be better to exclude these studies from the results (Sterne et al., 2001). The reasons based on which any studies have been excluded will be provided with full detail in a supplementary table.

Publication bias will be assessed for syntheses including five or more studies using a funnel plot and the Egger regression asymmetry test.

Strategy for data synthesis

The impact of physical activity, smoking and alcohol consumption on healthy ageing across the lifespan will be presented. In particular, the different levels of these factors and how these are related to healthy ageing will be extracted.

We will consider the different levels/measurements of these factors as statistically significant if i) the reported P-value is less than 0.05; ii) the author reported that there was a significant association; and iii) the 95% confidence interval around odds ratio or a similar statistic did not include 1.

Studies that refer no impact of the aforementioned determinants will also be presented. In addition, any concerns regarding the generalisability of the findings will be mentioned. For example, a small sample size, ethnicity/gender bias or important number of participants that were lost to follow-up will be referred.

Assuming non-substantial heterogeneity in study characteristics (e.g. determinant measurements, outcomes, etc.), we will carry out a meta-analysis to pool together effect estimates for each determinant. I-squared statistic will be used in order to assess the heterogeneity of the studies and based on this and the theoretical ground of the included studies, a fixed or random effect meta-analysis will be performed (Higgins et al., 2011). If a meta-analysis is not feasible due to amplified heterogeneity for the outcome and/or the lifestyle determinant measurement, a narrative synthesis for summarising the evidence will be performed.

In case of any discrepancies among the studies, study designs, populations, precision and publication bias will be assessed. Quality of our evidence will be considered as high when further research is very unlikely to change our confidence in the estimate of effect, moderate when further research is likely to have a considerable impact on our confidence and may change our estimate. The quality of evidence will be considered as low, in case that further research is very likely to have an important impact on our confidence in the estimate of effect, and is likely to change the estimate; and very low, when we are not certain about the estimate effect.

Quality of our findings will be assessed by performing a sensitivity analysis, should the selected studies permit it. For example, we will repeat the meta-analysis by excluding studies with low quality and see what will be the impact of it to our conclusions.

Analysis of subgroups or subsets

If the selected data permit, sub-group analyses will be carried out so as to stratify for different physical activity measurements (i.e subjective/self-report vs objective assessment of PA).

Dissemination plans

The findings of this review will be presented to relevant scientific conferences, for example ATHLOS consortium workshops, as well as to academic journals. Furthermore, conclusions will be disseminated through research and university networks.

Contact details for further information

Christina Daskalopoulou

King's College London, Institute of Psychiatry, Psychology & Neuroscience, Centre for Global Mental Health, Department of Health Services and Population Research, David Goldberg Centre, De Crespigny Park, Denmark Hill, London SE5 8AF, UK.

christina.daskalopoulou@kcl.ac.uk

Organisational affiliation of the review

King's College London, Institute of Psychiatry, Psychology & Neuroscience, Centre for Global Mental Health, Department of Health Services and Population Research

http://www.kcl.ac.uk/ioppn/depts/hspr/index.aspx

Review team

Ms Christina Daskalopoulou, King's College London
Dr Matthew Prina, King's College London
Professor Martin Prince, King's College London
Dr Artemis Koukounari, King's College London

Collaborators

Dr Carolina Kralj, King's College London

Anticipated or actual start date

14 March 2016

Anticipated completion date

31 December 2016

Funding sources/sponsors

Department of Health Services and Population Research, Institute of Psychology, Psychiatry and Neuroscience at King’s College London is the Sponsor, meaning that it has overall control of the data. This project falls under the ATHLOS (Ageing Trajectories of Health: Longitudinal Opportunities and Synergies) project, funded by the European Union’s Horizon 2020 Research and Innovation Programme under grant agreement number 63531.

Conflicts of interest

None known

Language

English

Country

England

Subject index terms status

Subject indexing assigned by CRD

Subject index terms

Age Factors; Aging; Alcohol Drinking; Exercise; Health; Health Status; Humans; Motor Activity; Risk Factors; Smoking

Reference and/or URL for protocol

http://www.crd.york.ac.uk/PROSPEROFILES/38130_PROTOCOL_20160326.pdf

Stage of review

Ongoing

Date of registration in PROSPERO

26 April 2016

Date of publication of this revision

26 April 2016

Stage of review at time of this submission	Started	Completed
Preliminary searches	Yes	No
Piloting of the study selection process	Yes	No

Formal screening of search results against eligibility criteria	No	No
Data extraction	No	No
Risk of bias (quality) assessment	No	No
Data analysis	No	No

PROSPERO
International prospective register of systematic reviews
The information in this record has been provided by the named contact for this review. CRD has accepted this information in good faith and registered the review in PROSPERO. CRD bears no responsibility or liability for the content of this registration record, any associated files or external websites.
