# Supplementary material for: Associations of smoking and alcohol consumption with healthy ageing: a systematic review and meta-analysis of longitudinal studies
Source: BMJ Open. 2018 Apr 17;8(4):e019540. doi: 10.1136/bmjopen-2017-019540 (PMC5905752; doi:10.1136/bmjopen-2017-019540)
Supplement: Supplementary file 3 [file bmjopen-2017-019540supp003.pdf]

Systematic Review Protocol: ATHLOS: Impact of Physical Activity, Smoking and Alcohol on Healthy Ageing throughout the Life Span: protocol for a systematic review.

Embase, Psychinfo (OVID interface) and Cochrane Library

(health* ageing) OR (health* aging) OR (successful ageing) OR (successful aging) OR (positive ageing) OR (positive aging) OR (productive ageing) OR (productive aging) OR (ageing well) OR (aging well) OR (optim* ageing) OR (optim* aging) OR (unimpaired ageing) OR (unimpaired aging) OR (effective ageing) OR (effective aging) OR (robust ageing) OR (robust aging) OR (exceptional survival)

AND ((smoking) OR (tobacco) OR (cigarette*) OR (physical activity) OR (physical inactivity) OR (exercise*) OR (alcohol) OR (Alcohol Drinking))

MedLine search (PubMed/PMC)

(("health* ageing") OR ("health* aging") OR ("successful ageing") OR ("successful aging") OR ("positive ageing") OR ("positive aging") OR ("productive ageing") OR ("productive aging") OR ("ageing well") OR ("aging well") OR ("optim* ageing") OR ("optim* aging") OR ("unimpaired ageing") OR ("unimpaired aging") OR ("effective ageing") OR ("effective aging") OR ("robust ageing") OR ("robust aging") OR ("exceptional survival")) AND (("motor activity"[MeSH Terms]) OR ("smoking"[MeSH Terms]) OR ("Alcohol Drinking"[MesH Terms]))
