# Supplementary material for: Associations of smoking and alcohol consumption with healthy ageing: a systematic review and meta-analysis of longitudinal studies
Source: BMJ Open. 2018 Apr 17;8(4):e019540. doi: 10.1136/bmjopen-2017-019540 (PMC5905752; doi:10.1136/bmjopen-2017-019540)
Supplement: Supplementary file 4 [file bmjopen-2017-019540supp004.pdf]

Table A1: Areas of information included in the definition of healthy ageing

Authors	Survival	Health Status	Physical Performance	Diseases	Mental Health	Subjective Measurements	Others
Andrews et al., 2002			x		x		
Bell et al., 2014	x		x	x	x		
Britton et al., 2008		x	x	x	x		
Burke et al., 2001				x			
Ford et al., 2000							x
Gu et al., 2009	x		x				
Guralnik & Kaplan, 1989			x				
Gureje et al., 2014			x	x		x	
Hamer et al., 2013			x	x	x		
Hodge, English et al., 2013	x		x	x	x		
Hodge, O'Dea et al., 2014	x		x	x	x		
Kaplan et al., 2008		x	x				
LaCroix et al., 2016	x		x	x			
Li et al., 2001			x		x	x	
Liu & Su, 2016			x	x	x		
Newman et al., 2003			x	x	x		
Newson et al., 2010	x			x			
Pruchno & Wilson-Genderson, 2015			x	x		x	
Reed et al., 1998	x		x	x	x		
Sabia et al., 2012			x	x	x		
Sarnak et al., 2008			x	x	x		
Shields & Martel, 2006		x	x		x		
Sun et al., 2011	x		x	x	x		
Tampubolon, 2016							x
Terry et al., 2005	x			x	x		
Vaillant & Mukamal, 2001	x		x			x	x
Vaillant & Western, 2001	x		x			x	x
Willcox et al., 2006	x		x	x	x		
**Areas of information included in the definition of Healthy Ageing**	**12**	**3**	**23**	**18**	**16**	**5**	**4**
