# Supplementary material for: Associations of smoking and alcohol consumption with healthy ageing: a systematic review and meta-analysis of longitudinal studies
Source: BMJ Open. 2018 Apr 17;8(4):e019540. doi: 10.1136/bmjopen-2017-019540 (PMC5905752; doi:10.1136/bmjopen-2017-019540)
Supplement: Supplementary file 5 [file bmjopen-2017-019540supp005.pdf]

Table A2: Quality assessment of the studies – aggregated results of QUIPS tool

Author, Year of Publication	The study sample represents the population of interest on key characteristics, sufficient to limit potential bias of the observed relationship between PF and outcome.	Loss to follow-up is not associated with key characteristics sufficient to limit potential bias to the observed relationship between PF and outcome.	PF is adequately measured in study participants to sufficiently limit potential bias.	Outcome of interest is adequately measured in study participants to sufficiently limit potential bias.	Important potential confounders are appropriately accounted for, limiting potential bias with respect to the relationship between PF and outcome.	The statistical analysis is appropriate for the design of the study, limiting potential for presentation of invalid or spurious results.	Total
Andrews et al., 2002	Low Bias	Moderate Bias	Moderate Bias	Low Bias	Moderate Bias	Low Bias	Moderate Bias
Bell et al., 2014	Low Bias	Low Bias	Low Bias	Low Bias	Moderate Bias	Low Bias	Low Bias
Britton et al., 2008	Low Bias	Moderate Bias	Low Bias	Low Bias	Moderate Bias	Low Bias	Low Bias
Burke et al., 2001	Low Bias	Low Bias	Low Bias	Moderate Bias	Moderate Bias	Low Bias	Low Bias
Ford et al., 2000	Low Bias	Moderate Bias	Moderate Bias	Moderate Bias	Low Bias	Low Bias	Moderate Bias
Gu et al., 2009	Low Bias	Low Bias	Low Bias	Low Bias	Moderate Bias	Low Bias	Low Bias
Guralnik & Kaplan, 1989	Low Bias	Moderate Bias	Moderate Bias	Moderate Bias	Moderate Bias	Low Bias	Moderate Bias
Gureje et al., 2014	Low Bias	Moderate Bias	Low Bias	Low Bias	Moderate Bias	Low Bias	Low Bias
Hamer et al., 2013	Low Bias	Low Bias	Moderate Bias	Low Bias	Low Bias	Low Bias	Low Bias
Hodge, English et al., 2013	Low Bias	Moderate Bias	Low Bias	Low Bias	Low Bias	Low Bias	Low Bias
Hodge, O'Dea et al., 2014	Low Bias	High Bias	Low Bias	Low Bias	Low Bias	Low Bias	Moderate Bias
Kaplan et al., 2008	Low Bias	Moderate Bias	Low Bias	Moderate Bias	Low Bias	Low Bias	Low Bias
LaCroix et al., 2016	Low Bias	Moderate Bias	Low Bias	Low Bias	Moderate Bias	Low Bias	Low Bias
Li et al., 2001	Low Bias	High Bias	Low Bias	Low Bias	Moderate Bias	Low Bias	Moderate Bias
Liu & Su, 2016	Low Bias	Moderate Bias	Low Bias	Low Bias	Moderate Bias	Low Bias	Low Bias
Newman et al., 2003	Low Bias	Low Bias	Low Bias	Low Bias	Moderate Bias	Low Bias	Low Bias
Newson et al.,	Low Bias	Moderate Bias	Low Bias	Low Bias	Moderate Bias	Low Bias	Low Bias

2010							
Pruchno & Wilson-Genderson, 2015	Low Bias	Moderate Bias	Low Bias	Low Bias	Moderate Bias	Low Bias	Low Bias
Reed et al., 2011	Low Bias	Moderate Bias	Low Bias	Low Bias	Low Bias	Moderate Bias	Low Bias
Sabia et al., 2012	Low Bias	Low Bias	Low Bias	Moderate Bias	Low Bias	Low Bias	Low Bias
Sarnak et al., 2008	Low Bias	Moderate Bias	Moderate Bias	Low Bias	Moderate Bias	Low Bias	Moderate Bias
Shields & Martel, 2006	Low Bias	Moderate Bias	Low Bias	Low Bias	Low Bias	Low Bias	Low Bias
Sun et al., 2011	Low Bias	Low Bias	Low Bias	Low Bias	Low Bias	Low Bias	Low Bias
Tampubolon, 2016	Low Bias	Low Bias	Low Bias	Low Bias	Moderate Bias	Low Bias	Low Bias
Terry et al., 2005	Low Bias	Low Bias	Low Bias	Moderate Bias	Moderate Bias	Moderate Bias	Moderate Bias
Vaillant & Mukamal, 2001	High Bias	Moderate Bias	Low Bias	Moderate Bias	Moderate Bias	Low Bias	High Bias
Vaillant & Western, 2001	High Bias	Low Bias	Low Bias	Low Bias	Moderate Bias	Low Bias	Moderate Bias
Willcox et al., 2006	Moderate Bias	Moderate Bias	Low Bias	Low Bias	Moderate Bias	Low Bias	Moderate Bias
