# Supplementary figures and images for: Associations of smoking and alcohol consumption with healthy ageing: a systematic review and meta-analysis of longitudinal studies
Source: BMJ Open. 2018 Apr 17;8(4):e019540. doi: 10.1136/bmjopen-2017-019540 (PMC5905752; doi:10.1136/bmjopen-2017-019540)

Figure A1: Smoking and healthy ageing-age stratified

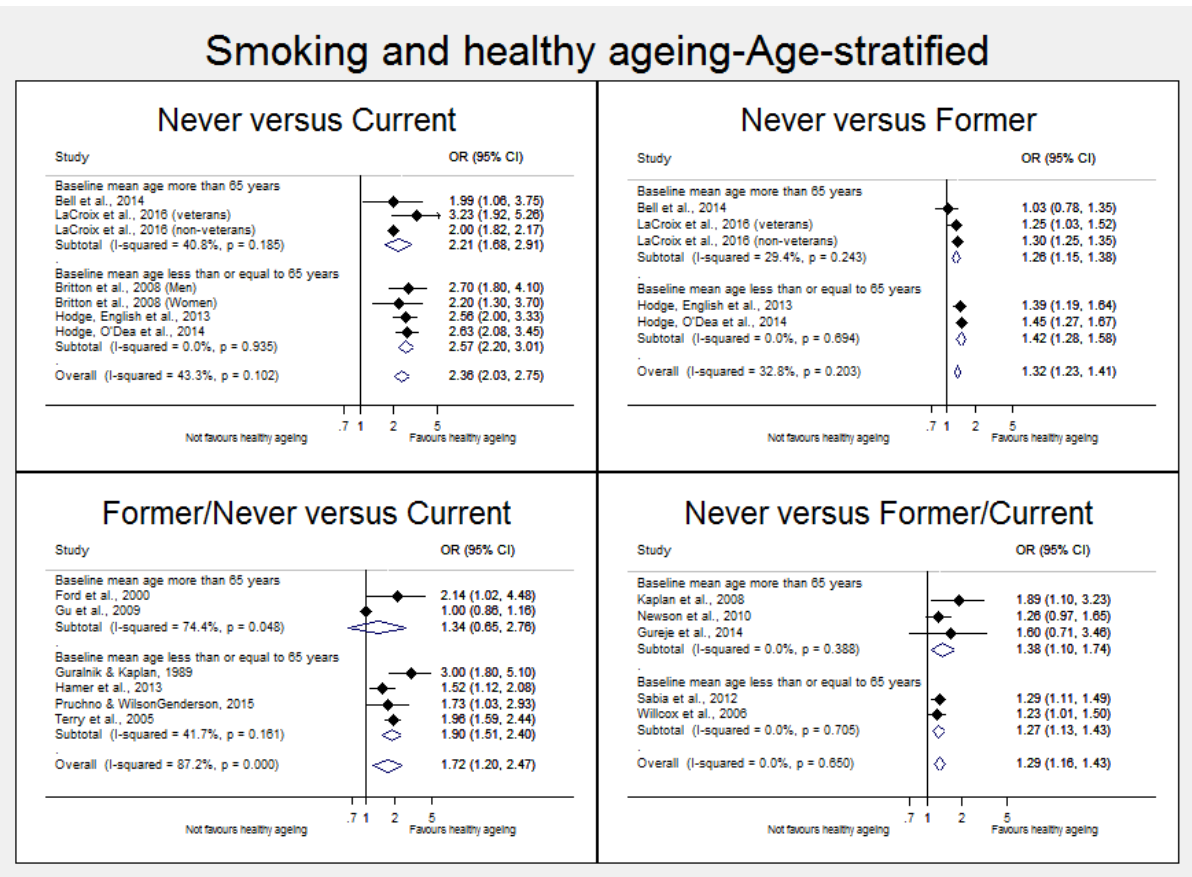

Supplement: Supplementary file 6 [file bmjopen-2017-019540supp006.pdf]

Figure A2: Smoking and healthy ageing-follow-up time stratified

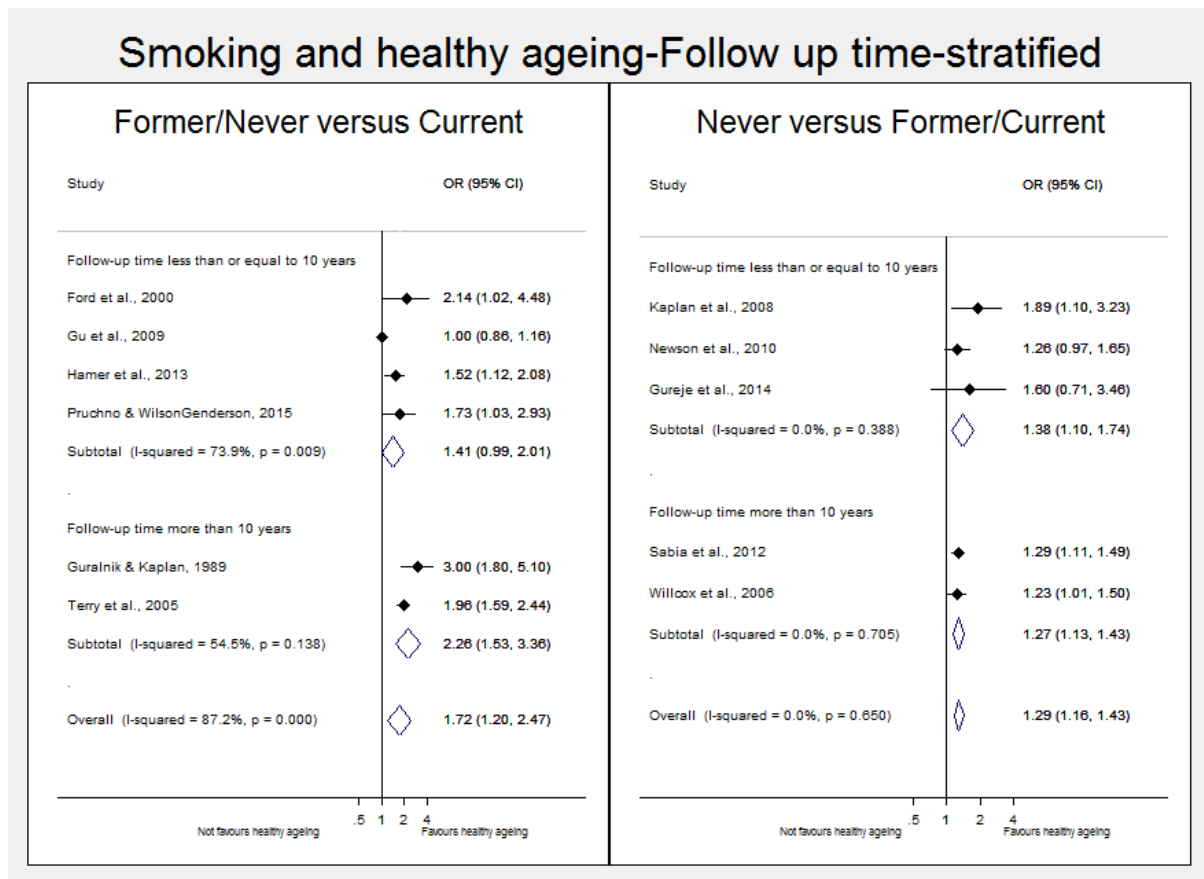

Supplement: Supplementary file 7 [file bmjopen-2017-019540supp007.pdf]

Figure A3: Alcohol and healthy ageing-follow-up stratified

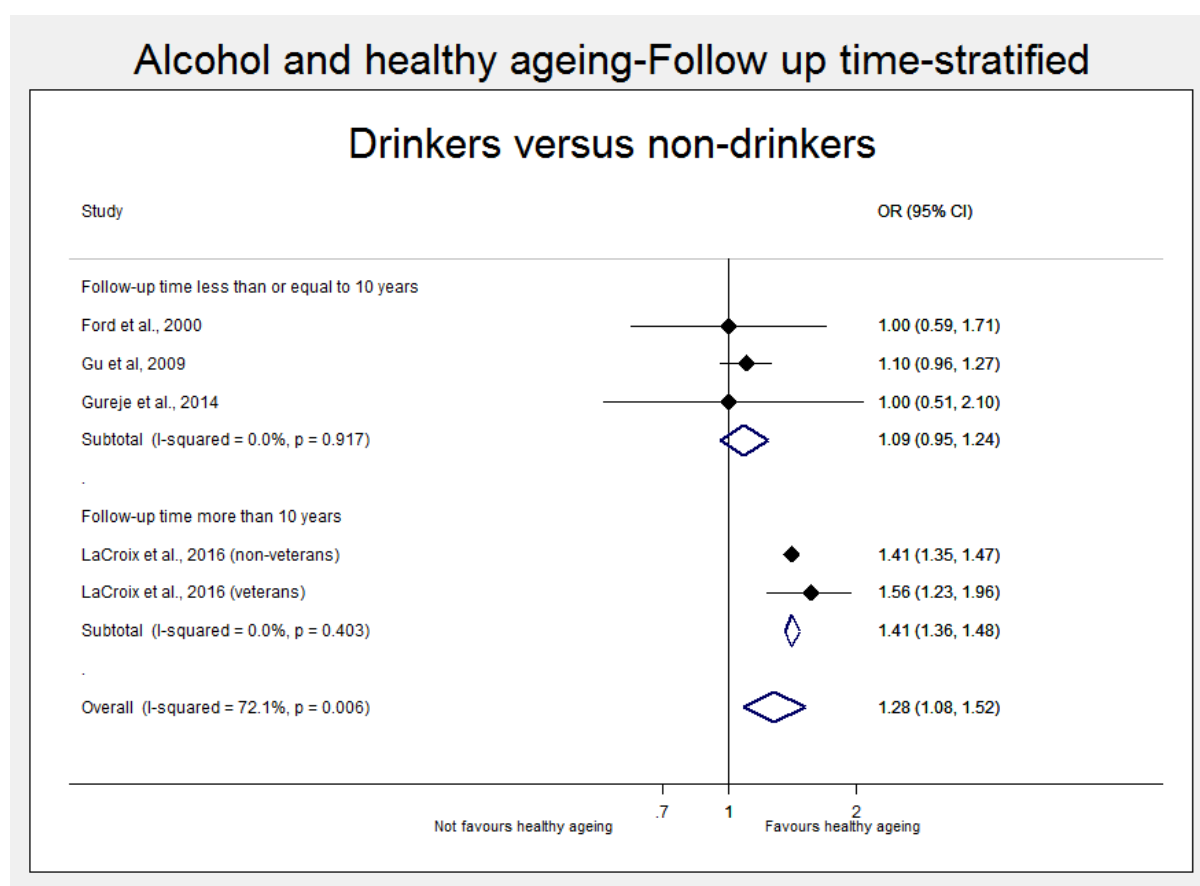

Supplement: Supplementary file 8 [file bmjopen-2017-019540supp008.pdf]
