# Supplementary material for: Associations of smoking and alcohol consumption with healthy ageing: a systematic review and meta-analysis of longitudinal studies
Source: BMJ Open. 2018 Apr 17;8(4):e019540. doi: 10.1136/bmjopen-2017-019540 (PMC5905752; doi:10.1136/bmjopen-2017-019540)
Supplement: Supplementary file 9 [file bmjopen-2017-019540supp009.pdf]

Table A3: Sensitivity analysis of meta-analytic results

Analysis	Number of Studies	DerSimonian-Laird ORs and 95%CI	p-value	I^2	Paule-Mandel ORs and 95%CI	p-value	I^2
Never vs Current Smokers	7*	2.36 (2.03-2.75)	<.001	43.3%	2.29 (2.03-2.59)	<.001	23.6%
Never vs Former Smokers	5†	1.32 (1.23-1.41)	<.001	32.8%	1.31 (1.21-1.43)	<.001	50.0%
Past/Never vs Current	6‡	1.72 (1.20-2.47)	.003	87.2%	1.69 (1.25-2.29)	<.001	81.3%
Never vs Past/Current	5§	1.29 (1.16-1.43)	<.001	0.0%	1.29 (1.16-1.43)	<.001	0.0%
Drinkers vs Non-Drinkers	5‖	1.28 (1.08-1.52)	.004	72.1%	1.29 (1.10-1.50)	.002	68.2%
Light vs Non-Drinkers	3¶	1.12 (1.03-1.22)	.010	0.0%	1.12 (1.03-1.22)	.010	0.0%
Moderate vs Non-Drinkers	4**	1.35 (0.93-1.97)	.112	71.4%	1.37 (0.90-2.08)	.138	77.0%
High to Non-Drinkers	3††	1.25 (1.09-1.44)	.002	0.0%	1.25 (1.09-1.44)	.002	0.0%

OR: Odds Ratio; CI: Confidence Interval

* Bell et al., 2014; Britton et al., 2008 (Males); Britton et al., 2008 (Females); Hodge, English et al., 2013; Hodge, O'Dea et al., 2014; LaCroix et al., 2016 (veterans); LaCroix et al., 2016 (non-veterans)

† Bell et al., 2014; Hodge, English et al., 2013; Hodge, O'Dea et al., 2014; LaCroix et al., 2016 (veterans); LaCroix et al., 2016 (non-veterans)

‡ Ford et al., 2000; Gu et al., 2009; Guralnik & Kaplan, 1989; Hamer et al., 2013; Pruchno & Wilson-Genderson, 2015; Terry et al., 2005

§ Kaplan et al., 2008; Newson et al., 2010; Gureje et al., 2014; Sabia et al., 2012; Willcox et al., 2006

‖ Ford et al., 2000; Gu et al, 2009; Gureje et al., 2014; LaCroix et al., 2016 (veterans); LaCroix et al., 2016 (non-veterans)

¶ Hodge, English et al., 2013; Hodge, O'Dea et al., 2014; Sun et al., 2011

** Britton et al., 2008 (Males); Britton et al., 2008 (Females); Guralnik & Kaplan, 1989; Sun et al., 2011

†† Hodge, English et al., 2013; Hodge, O'Dea et al., 2014; Sun et al., 2011
