# Supplementary material for: Associations of smoking and alcohol consumption with healthy ageing: a systematic review and meta-analysis of longitudinal studies
Source: BMJ Open. 2018 Apr 17;8(4):e019540. doi: 10.1136/bmjopen-2017-019540 (PMC5905752; doi:10.1136/bmjopen-2017-019540)
Supplement: Author's manuscript [file bmjopen-2017-019540.draft_revisions.pdf]

BMJ Open is committed to open peer review. As part of this commitment we make the peer review history of every article we publish publicly available.

When an article is published we post the peer reviewers' comments and the authors' responses online. We also post the versions of the paper that were used during peer review. These are the versions that the peer review comments apply to.

The versions of the paper that follow are the versions that were submitted during the peer review process. They are not the versions of record or the final published versions. They should not be cited or distributed as the published version of this manuscript.

BMJ Open is an open access journal and the full, final, typeset and author-corrected version of record of the manuscript is available on our site with no access controls, subscription charges or pay-per-view fees (http://bmjopen.bmj.com).

If you have any questions on BMJ Open's open peer review process please email info.bmjopen@bmj.com

Associations of Smoking and Alcohol Consumption with Healthy Ageing: A Systematic Review and Meta-analysis of longitudinal studies

Journal:	*BMJ Open*
Manuscript ID	bmjopen-2017-019540
Article Type:	Research
Date Submitted by the Author:	11-Sep-2017

Complete List of Authors:	Daskalopoulou, Christina; King's College London, Health Service and Population Research Centre for Global Mental Health & Primary Care Research<br>Stubbs, B; King's College London, Health Service and Population Research Department; South London and Maudsley NHS Foundation Trust, Physiotherapy Department<br>Kralj, Carolina; King's College London, Health Service and Population Research Centre for Global Mental Health & Primary Care Research<br>Koukounari, Artemis; Liverpool School of Tropical Medicine, Department of Clinical Sciences<br>Prince, Martin; King's College London, Health Service and Population Research Centre for Global Mental Health & Primary Care Research<br>Prina, A.Matthew; King's College London, Health Service and Population Research Centre for Global Mental Health & Primary Care Research

**Primary Subject Heading**:	Public health
Secondary Subject Heading:	Global health, Health policy, Public health, Smoking and tobacco
Keywords:	healthy ageing, smoking, alcohol consumption, drinking, meta-analysis

SCHOLARONE™ Manuscripts

Associations of Smoking and Alcohol Consumption with Healthy Ageing: A Systematic Review and Meta-analysis of longitudinal studies

Christina Daskalopoulou, MSc[1], Brendon Stubbs, PhD[2,3], Carolina Kralj, MD[1], Artemis Koukounari, PhD[4], Martin Prince, MD, PhD[1], and Matthew A. Prina, PhD[2]

[*] Address for correspondence: Mrs C. Daskalopoulou, King's College London, Institute of Psychiatry, Psychology and Neuroscience, David Goldberg Centre, De Crespigny Park, London SE5 8AF, UK. (Email: christina.daskalopoulou@kcl.ac.uk.com)

For peer review only

[1] Health Service and Population Research Department, King's College London, Institute of Psychiatry, Psychology and Neuroscience, Centre for Global Mental Health, London, UK;

[2] Health Service and Population Research Department, King's College London, Institute of Psychiatry, Psychology and Neuroscience, London, UK;

[3] Physiotherapy Department, South London and Maudsley NHS Foundation Trust, London, UK;

[4] Department of Clinical Sciences, Liverpool School of Tropical Medicine, Liverpool, UK.

For peer review only - http://bmjopen.bmj.com/site/about/guidelines.xhtml

Abstract (298 words)

Objectives The number of older people is growing across the world, however quantitative synthesis of studies examining the impact of lifestyle factors on the ageing process are rare. We conducted a systematic review and meta-analysis of longitudinal studies to synthesise the associations of smoking and alcohol consumption with healthy ageing (HA).

Methods Major electronic databases were searched from inception to March 2017 (PROSPERO reg. CRD42016038130). Studies were assessed for methodological quality. Random-effect meta-analysis was performed to calculate pooled odds ratios (OR) and 95% confidence intervals (CI).

Results In total, we identified 28 studies (n=184,543); 27 studies reported results on smoking, 22 on alcohol consumption. 23 studies reported a significant positive association of never or former smoking with HA and 4 non-significant. 12 studies reported a significant positive association of alcohol consumption with HA, 9 no association, 1 negative. Meta-analysis revealed increased pooled OR of HA for never smokers compared to current smokers (2.36, 95%CI 2.03-2.75), never smokers compared to former smokers (1.32, 95%CI 1.23-1.41), former or never smokers compared to current smokers (1.72, 95%CI 1.20-2.47), never smokers compared to past or current smokers (1.29, 95%CI 1.16-1.43); drinkers compared to non-drinkers (1.28, 95%CI 1.08-1.52), light drinkers compared to non-drinkers (1.12, 95%CI 1.03-1.22), moderate drinkers compared to non-drinkers (1.36, 95%CI 0.93-1.97) and high drinkers compared to non-drinkers (1.25, 95%CI 1.09-1.44). There was considerable heterogeneity in the definition and measurement of HA and alcohol consumption.

Conclusions There is consistent evidence from longitudinal studies that smoking is negatively associated with HA. The associations of alcohol consumption with HA are

For peer review only - http://bmjopen.bmj.com/site/about/guidelines.xhtml

For peer review only

equivocal. Future research should focus on the implementation of a single metric of HA, on the use of consistent drinking assessment among studies and on a full-range of confounding adjustment. Our research also highlighted the limited research on ageing in low-and-middle income countries.

Key words: healthy ageing; smoking; alcohol consumption; drinking; meta-analysis.

For peer review only

For peer review only - http://bmjopen.bmj.com/site/about/guidelines.xhtml

Strengths and limitations of this study

- This is the first systematic review and meta-analysis trying to quantify the associations of smoking and alcohol consumption with healthy ageing; our findings were also adjusted for publication bias.
- Independent double screening, searching of previous systematic reviews in the field and the reference lists of the eligible studies as well as not excluding non-English studies guarantee that limited number of studies may have not been considered.
- Heterogeneity in the definition and measurement of healthy ageing as well as different confounding adjustment in the initial studies may have influenced our meta-analytical results.
- The number of studies included in the meta-analysis regarding the associations of alcohol consumption was small due to the great heterogeneity in the measurement of it.
- Our findings may not be generalisable due to the limited research in low and middle income countries.

For peer review only

For peer review only - http://bmjopen.bmj.com/site/about/guidelines.xhtml

Words: 3,975

Introduction

Smoking has emerged as the leading contributing factor of many respiratory diseases and one of the most important risk factors for cardiovascular diseases, cancers of several organs and many other pathological conditions.[1] In systematic reviews focusing on elderly people, smoking is associated with premature mortality[2] and an increased incidence of Alzheimer's disease.[3] Smoking is also linked with a considerable societal cost as smoking-related absenteeism and premature deaths create an additional burden to the healthcare system and to the economy in general.[4] The healthcare cost of tobacco-related diseases accounted for almost 6% of the global health expenditures in 2012.[5]

Tobacco use and harmful use of alcohol are recognised by the United Nations Political Declaration on Non-Communicable Diseases (NCDs) among the most common risk factors.[6] Harmful use of alcohol results approximately in 3.3 million deaths annually (5.9% of all deaths) and alcohol-attributable deaths are the highest for middle-aged people.[7] Older people's sensitivity in alcohol increases whereas tolerance decreases, meaning that even the same amount of alcohol can create problems in later years.[8] In addition, older people with drinking problems often do not present to services due to stigma associated with the condition, and when they do, their symptoms can be easily confused with physical and mental issues associated with ageing.[9] Nevertheless, studies showed that light to moderate alcohol consumption is associated with a decreased risk in dementia,[10] as well as with a reduced risk for cardiovascular disease mortality, incident coronary heart disease, incident stroke, stroke mortality and all-cause mortality.[11]

Drinking and smoking are correlated in the general population[12] with smokers being more likely to drink and vice versa.[13] People who are smokers and heavy drinkers exhibit the highest mortality rate as indicated in a study of middle-aged male participants;[14] another

For peer review only - http://bmjopen.bmj.com/site/about/guidelines.xhtml

study also showed that men who smoked and drank more than 15 units per week of alcohol had almost three times higher all-cause mortality rate compared to non-smokers and non-drinkers.[15] As populations are ageing rapidly, with one in eight people currently aged 60 and over and with these estimates predicted to increase,[16] it is critical to understand the factors that will enable people to live longer and age in a healthy way.

To address the need for more accurate estimates of the relationship between smoking and healthy ageing and alcohol consumption and healthy ageing we conducted a systematic review and meta-analysis of longitudinal studies which examined the associations of these factors with the ageing process.

For peer review only

Methods

Search strategy and selection criteria

This systematic review has been written following the Preferred Reporting Items for a Systematic Review and Meta-Analysis (PRISMA) and the guidelines for Meta-Analyses Of Observational Studies in Epidemiology (MOOSE) to ensure accuracy and comprehensiveness.[17,18] (Appendix 1). A review protocol was prospectively registered in the international database of prospectively registered systematic reviews (PROSPERO) under registration number CRD42016038130 (Appendix 2). As part of a larger body of work considering modifiable lifestyle factors and healthy ageing, we originally planned to carry out a review focusing on the following: physical activity, smoking and alcohol consumption. The current systematic review specifically focuses on the findings related to smoking, alcohol consumption and healthy ageing outcomes since a sufficient amount of literature was identified on this topic alone. Our findings regarding healthy ageing and physical activity have been presented elsewhere.[19]

Two authors (C.D, C.K) searched MEDLINE (PubMed/PubMed Central interface), EMBASE (OVID interface), Psychinfo (OVID interface) and CENTRAL (Cochrane Central

For peer review only - http://bmjopen.bmj.com/site/about/guidelines.xhtml

Register of Controlled Trials) from inception up to April 2016. Searching methodology included any related term or synonym to healthy ageing and text word related to physical activity, smoking and alcohol consumption (Appendix 3). Other relevant systematic reviews and reference lists of the eligible studies were also searched. Finally, a second search was performed on 15th of March 2017 to include studies that were recently published.

Eligible studies had to meet the following criteria: i. being published in an electronic journal article; ii. constitute an original peer-reviewed longitudinal study; and iii. report any kind of longitudinal association between smoking and/or alcohol consumption and healthy ageing. Data on smoking and alcohol, through self-report and/or specific questionnaires, referred either to the current status and/or across the life span. The primary outcome of this review was health status measured by healthy ageing, and any other term related to it (e.g. successful ageing, active ageing, healthy survival etc.). Studies whose primary goal was the examination of a different determinant but included the aforementioned factors as covariates were also included. Due to the heterogeneity of the healthy ageing definition, studies reporting the latter as multiple outcomes or based solely on self-report were excluded. Studies that included cohorts of individuals who were institutionalised or hospitalised and animal studies were also excluded. No language restriction was applied.

In order to store the studies that were retrieved by the electronic search we used an EndNote (ENDNOTE X7, Thomson Reuters) library. After removal of duplicates, the two independent reviewers screened titles and abstracts of all potentially eligible papers. At the end of this procedure, any disagreement was solved by discussion. In case that an agreement could not be achieved, eligibility of the study was judged by discussion with a third senior researcher (A.M.P.). In case that full text could not be retrieved, the corresponding author of the paper was contacted via e-mail.

For peer review only - http://bmjopen.bmj.com/site/about/guidelines.xhtml

Data extraction and quality assessment

C.D. and C.K. independently extracted data from each study and a random sample of them was cross-checked by A.M.P. Setting/country of the study, data collection period, follow-up year, sample size, population, and baseline age information was recorded for each study. Definition and measurement of the healthy ageing outcome, of smoking and alcohol consumption were also recorded, as well as the odds ratios (OR) (or any other related statistic) and the 95% confidence intervals (CI). Reported statistics were extracted for the crude/unadjusted model and the most adjusted one reported in the study.

The methodological quality of the included studies was assessed by using the Quality in Prognosis Studies (QUIPS) tool. QUIPS evaluates six potential components of bias: inclusion, attrition, prognostic factor measurement, confounders, outcome measurement, and analysis and reporting.[20] During the application of the QUIPS tool smoking and/or alcohol consumption were considered as the only prognostic factors and all other variables, used as explanatory variables of the model, were considered as confounders. Since we included only longitudinal studies, the existence of attrition was expected. In the scenario that the attrition rate was high, authors' explanations were required in order to evaluate the study as bearing low risk of bias. Finally, the reliability of statistical models was evaluated according to the data presented; for instance, the reliability of studies that included results only for the statistical significant factors was judged with caution.

Data analysis

We performed meta-analyses in order to produce a pooled effect size estimate for the relationship between smoking and healthy ageing and alcohol consumption and healthy ageing. Separate meta-analyses were performed for the following categories:

For peer review only - http://bmjopen.bmj.com/site/about/guidelines.xhtml

Smoking: i) past and never smokers compared to current smokers; ii) former or never smokers compared to current smokers; iii) never smokers compared to former or current smokers.

Alcohol: For studies that provided alcohol quantities, we converted grams to drinks by assuming that 12 grams of alcohol are approximately one drink so as to have an alcohol consumption variable comparable among the studies.[21] In studies that provided alcohol consumption in an interval we took the middle of the interval as the most likely value. We created the following categories: light (< 1 drink/day), moderate (1–2 drinks/day), and high (> 2–4 drinks/day) consumption that were compared to never drinkers. Studies that did not report specific alcohol quantity but characterised the participants as drinkers or non-drinkers were examined separately.

We produced a pooled effect estimate of the aforementioned categories for the most adjusted models. In order to obtain results, at least three studies had to be included in the analysis. Due to the expected heterogeneity, a random effects meta-analysis using the DerSimonian-Laird model was performed.[22] If a study reported different results for men and women, both results were included, except in cases where a result for the mixed population was also provided; the same rationale was applied to cases that reported statistics per different sub-groups in the cohort. Heterogeneity was assessed with I^2 statistic for each analysis,[23] publication bias[24] was assessed with Egger bias test.[25] Finally, a trim-and-fill adjusted analysis was conducted to adjust for potential publication bias.[26] All analyses were performed using STATA 14 IC statistical software.

Results

The search identified 6,706 papers from the databases and 30 additional papers were obtained from other sources. After removing of duplicates and of papers that were conference papers, cross-sectional studies or animal studies, 73 papers were considered for full text-review. We

For peer review only - http://bmjopen.bmj.com/site/about/guidelines.xhtml

excluded 42 papers based on specific reasons after the full text-review and finally 28 studies are included in this report.[27-54] PRISMA flow diagram depicts the exact selection process **Figure 1**. Across the 28 eligible studies, there were 184,543 participants (almost 31% men), with sample size ranging from 456 to 68,153. Studies were published from 1989 to 2016 and half of them (14 out of 28) took place in USA. Studies were also conducted in England (four), in Australia (three), in China (two), in Canada (two), in Nigeria (one), in the Netherlands (one) and in Taiwan (one). Baseline age ranged from 14 to 109 years old and follow-up time from two years until death (>70 years old). Five studies examined men only and two studies examined women only; the majority of studies reported results for mixed populations (**Table 1**).

Healthy ageing, and any other term used as a synonym, was defined by including various domains of information in each study. These were grouped in the following categories: survival to a specific age or during follow-up, health status (either self-reported or measured by specific questionnaires), physical performance (including information regarding mobility, disabilities and/or difficulties in activities of daily living (ADL) and instrumental activities of daily living (iADL)), diseases (including chronic diseases and cancer), mental health and cognition status, subjective measurements of the participants (life satisfaction, happiness, and pain) and other (anthropometric measurements, personal assistance, social support). Most of the studies (23 out of 28) included physical performance to define healthy ageing and more than half of them included information regarding diseases and mental health (18 and 16 out of 28 respectively). Survival to a specific age was also an area often found in the definition of healthy ageing, whereas health status and subjective measurements were not so often included. Supplemental table: 'Table A1'.

In **Table 2** the analytical results of this systematic review are presented. Relevant statistics per study are provided for every group of smoking and alcohol consumption variable and for

For peer review only - http://bmjopen.bmj.com/site/about/guidelines.xhtml

the most and least adjusted models; confounders used for the final adjustment are also provided.

Quality Assessment

Of the 28 studies, one was evaluated as having high risk of bias, nine as moderate and 18 as having low. In aggregate, the quality of the included studies was above moderate. As it was expected, attrition and confounding were those issues that primarily contributed to moderate and high bias. Specifically, 18 out of 28 studies reported moderate or high risk of bias regarding the fact that the population lost to follow-up may be associated with key characteristics that could influence the observed relationship between the outcome and the factors. Similar results were also observed for the confounders' domain, where 18 out of 23 studies were characterised as having moderate risk of bias, meaning that important confounders may have not been appropriately accounted in the final model. Supplemental table: 'Table A2'.

Meta-analysis

Smoking and Healthy Ageing

From the 27 studies providing results for smoking we did a meta-analysis with 18 studies. We excluded studies that provided results using: i) healthy/successful years;[30,42] ii) Risk Ratio;[40] iii) coefficients from linear mixed models or generalised estimation equation models;[41,50] and iv) cigarette-packs.[45,52,53] All studies included in the meta-analysis were of moderate and low risk of bias.

Never smokers compared to current smokers had more than double increased odds of ageing healthily (OR 2.36, 95%CI 2.03-2.75, I^2 43.30%, 7 studies) even after adjusting for publication bias with the trim-and-fill algorithm (OR 2.11, 95%CI 1.82-2.45, 10 studies). Never smokers had increased odds ratios when compared to former smokers as well (OR

1.32, 95%CI 1.23-1.41, I^2 32.80%, 5 studies); no change of the combined effect estimate was observed by the trim-and-fill method. Never smokers were more likely to experience healthy ageing than current or former smokers (OR 1.29, 95%CI 1.16-1.43, I^2 0.00%, 5 studies) and this finding marginally altered when we adjusted for publication bias (OR 1.27, 95%CI 1.14-1.40, 9 studies). Never or former smokers were more likely to have a healthy late survival compared to current smokers (OR 1.72, 95%CI 1.20-2.47, I^2 87.20%, 6 studies) however, when we adjusted for publication bias this finding was no longer significant (OR 1.20, 95%CI 0.83-1.73, 9 studies).

There were also two studies that reported hazard ratios (HR);[47,48] the pooled HR of never smokers compared to current smokers was 1.55 (95%CI 1.18-2.02); while the pooled HR of former smokers compared to never smokers was 1.30 (95%CI 1.07-1.59). Egger test was not significant in any analysis that we performed (p-value>0.05), hence no evidence for publication bias was provided.

Alcohol Consumption and Healthy Ageing

From the 21 studies that provided statistics for the association of alcohol consumption with healthy ageing we performed meta-analyses using nine studies. We excluded studies for the following reasons: i) the standardisation of alcohol consumption was not possible;[28,38,40,44,46,48,52-54] ii) they provided healthy years[30] or β coefficients.[41,50]

Drinkers compared to non-drinkers had increased odds of ageing healthily (OR 1.28, 95%CI 1.08-1.52, I^2 72.10%, 5 studies); the same beneficial association of alcohol was also observed when we pooled results among studies that reported findings of light consumption (<1 drinker per day) and moderate consumption (1-2 drinks per day) compared to non-drinkers: (OR 1.12, 95%CI 1.03-1.22, I^2 0.00%, 3 studies); (OR 1.35, 95%CI 0.93-1.97, I^2 71.40%, 4 studies). However, in the latter comparison (i.e. moderate consumption compared to non-drinkers) results were not statistically significant (p-value 0.112). We also pooled results of

For peer review only - http://bmjopen.bmj.com/site/about/guidelines.xhtml

the studies reporting high (>2-4 drinks per day) consumption compared to non-drinkers; OR 1.25, (95%CI 1.09-1.44, I^2 0.00%, 3 studies). Results changed when we used the trim-and-fill algorithm only to the case of moderate to non-drinkers (OR 1.10, 95%CI 0.77-1.57, 6 studies). Egger test of bias was significant only in the analysis of light drinkers (p-value 0.043). Analytical results are presented in **Table 3**. The synthesis of the results is provided in **Figure 2** and **Figure 3**.

Discussion

Based on our knowledge, this systematic review is the first to examine the association of smoking and alcohol consumption with healthy ageing, which included a meta-analysis to produce a pooled effect estimate and also examined eligible studies for publication bias. 23 studies out of 27 reported a positive association between never or former smoking and healthy ageing and four reported a non-significant relationship. With regards to alcohol consumption, results were mixed but a positive association of drinking with healthy ageing was reported in 12 out of 22 studies. Nine studies did not find an association and one study reported a negative one.

The current systematic review adds to the plethora of evidence that smoking is associated with worse outcomes in older age. In fact, the only study that reported a positive association between current smoking and healthy ageing was that of Li et al. (2001)[40] where smokers exhibit increased risk ratios of healthy survival (RR 1.11, 95%CI 1.01-1.21); however authors recommend to not trusting this finding as the population attributable percentage (PARP) is very low after the five years of the follow-up (2.55%). In aggregate, being a non-smoker or a former smoker increases considerably the odds of ageing healthily. From our meta-analysis we found that never smokers have more than double the odds of a good late survival (OR 2.36, 95%CI 2.03-2.75) compared to current smokers. Furthermore, never smokers are also more likely to age in a healthy way by more than 30% (OR 1.32, 95%CI 1.23-1.41) compared

For peer review only - http://bmjopen.bmj.com/site/about/guidelines.xhtml

to former smokers. These findings indicate that smoking cessation is always beneficial and come in accordance with a 50 years study on doctors' mortality in relation with smoking. In this study, it was revealed that smoking cessation at age 60, 50, 40, or 30 years could increase life expectancy by 3, 6, 9, or 10 years respectively.[55] Our findings also confirm previous research examining the beneficial effect of non-smoking on successful ageing.[56] In addition, we found that never/former smokers compared to current smokers have an increased likelihood of healthy ageing (OR 1.72, 95%CI 1.20-2.47), which becomes non-significant when we adjust for publication bias (OR 1.20, 95%CI 0.83-1.73). This comes in agreement with the finding of a study examining the transitions within smoking categories and successful ageing, where former compared to current smokers cannot predict successful ageing.[57]

Positive associations between drinking and healthy ageing refer to limited alcohol consumptions; for example 1-40 gr per day,[37] 1-14 drinks per week,[38] 1-14 units per week for women and 1-21 units per week for men[46]. The one study reporting a negative association is that of Willcox et al. (2006);[54] however, this finding should be interpreted with caution as drinkers with more than 3 drinks per day are compared to non-drinkers only and there is no comparison among light or moderate drinkers and non-drinkers. From our meta-analysis, even if it included limited number of studies, we concluded that compared to never drinkers reasonable alcohol consumption is beneficial to healthy ageing; pooled OR for drinkers 1.28 (95%CI 1.08-1.52), light drinkers 1.12 (95%CI 1.03-1.22), moderate drinkers 1.36 (95%CI 0.93-1.97) and high drinkers 1.25 (95%CI 1.09-1.44). Nevertheless, associations are marginally statistically significant, while for the moderate category the pooled effect estimate is non-significant, so extra caution is needed before making a final conclusion.

Several studies of the associations of alcohol consumption with healthy ageing were excluded from our meta-analyses. However, among those there were seven studies that reported

For peer review only - http://bmjopen.bmj.com/site/about/guidelines.xhtml

positive association between alcohol consumption and healthy ageing,[38,40,41,44,46,48,50] four studies reported non-significant association,[28,30,52,53] and one study reported a negative association.[54] As a consequence, we expect that including these studies in our meta-analyses, the positive effect of light to moderate alcohol drinking would be reinforced. This finding comes in accordance with the fact that light to moderate alcohol consumption (≤1 drink per day) is also associated with a reduced risk of multiple cardiovascular outcomes,[11] better cognition and well-being,[58] and a reduced risk of substantial functional health decline.[59]

However, relating moderate alcohol consumption with health benefits should not come without question since there are many other issues to consider. Our results could be biased by confounders that have not been taken into account, such as health status or former and occasional drinkers to be counted as non-drinkers.[60] For instance, in the sub-group of drinkers the positive effect comes mainly from the study of LaCroix et al. (2016);[39] however, in that study former drinkers were also assumed as non-drinkers.

In addition, since typical drink sizes vary per country as well as the recommended daily or weekly amounts,[61] we cannot be sure that participants have been assigned to the correct drinking category. Our results should also be questioned regarding the fact that alcohol consumption was only assessed at baseline (exception to this is the study of Sun et al. (2011))[49]; during the baseline assessment some cohorts could have had middle-aged participants drinking up to four drinks per day and that could be considered a moderate consumption. However, older people do not have the same tolerance to alcohol[62] and for them moderate consumption should consist of up to two drinks per day. Likewise, it should have been considered that older adults tend to consume less alcohol than middle-aged groups[63] and hence baseline assessments perhaps are not representative of alcohol consumption in an older age. Nevertheless, more research is required in order to establish or reject the beneficial effects of limited alcohol consumption. In an extended systematic review and meta-analysis

For peer review only - http://bmjopen.bmj.com/site/about/guidelines.xhtml

of alcohol impact on mortality risk, the benefits of low-volume drinkers largely disappeared once studies were controlled for several design and methodological issues.[64]

Strengths and Limitations

The fact that this review was independently double screened, taking into account previous systematic reviews in the field and the reference lists of included papers allows a good amount of confidence that all relevant studies were included. Regarding the quality assessment of the studies, limited disagreement among the six different domains per study was reached between the two reviewers, who independently assessed them, concluding that the quality assessment tool was reliable and did not allow great amount of misjudgement. Attrition rate and not allowing important confounders to be included in the final models were important factors contributing to the quality of the considered studies. Hence future research should consider these domains more thoroughly.

However, the following limitations have to be taken into account. Smoking and alcohol consumption were not measured or/and defined in a consistent way among the different cohorts; hence it was not possible to create a pooled effect size estimate across all eligible studies. In addition, healthy ageing definition was a source of heterogeneity since each study provided its own definition. However, it has to be highlighted that majority of studies had definitions that included areas of physical performance, in agreement with the Depp and Jeste, (2006) review on successful ageing.[56] Alcohol measurement and drinking categorisation were also another part of the underlying heterogeneity. Similar comments have also been reported in other systematic reviews examining alcohol consumption.[64,65] Furthermore, self-reported questionnaires and the fact that each study was adjusted by using a different set of covariates, different follow-up time and attrition rate, and the different definition of healthy ageing increases the heterogeneity and the likelihood of bias. Especially for alcohol, research has shown that self-reported consumption is mostly underestimated.[66]

For peer review only - http://bmjopen.bmj.com/site/about/guidelines.xhtml

Due to the limited number of studies included in our meta-analyses we did not assess publication bias by funnel plots; instead we used Egger test and even though it was statistically non-significant in the majority of cases we applied the trim-and-fill method to examine the robustness of our findings. We used random effect meta-analysis to consider the heterogeneity of the studies, even though I^2 was not considerable in all analyses (**Table 3**). We did not assess heterogeneity with the Cochran's Q statistic due to the quite limited number of studies; performing meta-regression analysis and assessing the confounding effect of covariates was not possible either.

From our review, it is evident that the majority of the studies have been implemented in high-income countries (24 out of 28 studies). Thus, in accordance with a previous study,[67] there is limited research on ageing in low and middle income countries (LMIC), even though by 2050 80% of the people aged 60 years and over will live there.[68] In addition, the economic burden of smoking in those countries is the heaviest as it is estimated that by 2030 four out of five smoking-related deaths will occur there.[69] Nowadays, 80% of the more than one billion smokers live in LMIC.[70]

Conclusions

In conclusion, smoking abstinence and smoking cessation are positively associated with healthy ageing. As it takes more than 20 years for most smoking-related diseases to develop, the best practice would be to enforce smoking prevention policies, such as marketing bans and high taxation, and reduce smoking uptake among younger cohorts.[1] A positive relationship between limited consumptions of drinking could also be argued but more research should be done to reinforce this result. From our research it becomes evident that there is a need to make studies comparable in order to conclude with more confidence the generalisability of our findings. This could be achieved by adopting similar measurements of smoking and alcohol behaviour and by implementing a unanimous definition and metric of

For peer review only

For peer review only - http://bmjopen.bmj.com/site/about/guidelines.xhtml

healthy ageing. Finally, further research should focus on the examination of ageing in the older population of low-and-middle income countries.

Supplementary Material

Appendix1.docx (MOOSE checklist)

Appendix2.pdf (Prospero Protocol)

Appendix3.docx (Searching Methodology)

Table A1.docx (Healthy Ageing Definition)

Table A2.docx (Quality Assessment)

Contributors

CD designed the study, carried out the literature review, data extraction, statistical analysis, data interpretation, article preparation, article review and correspondence. BS contributed to the data interpretation and article review. CK contributed to the literature review and data extraction. AMP contributed to the study design, data interpretation, article preparation and article review. All authors contributed to the final report and approved the final version.

Acknowledgements

We thank Chih-Cheng Chang for his contribution to the translation of the non-English study.

Funding

This project falls under the ATHLOS (Ageing Trajectories of Health: Longitudinal Opportunities and Synergies) project, funded by the European Union's Horizon 2020 Research and Innovation Programme under grant agreement number 635316. The sponsor had no participation in the study design, data extraction, data interpretation, or writing of this study.

A. M. P. was supported by the MRC MR/K021907/1.

For peer review only

For peer review only - http://bmjopen.bmj.com/site/about/guidelines.xhtml

A. K. was supported by a grant funded by the National Institute for Health Research (NIHR) Biomedical Research Centre at South London and Maudsley National Health Service (NHS) Foundation Trust and Kings' College London where most of this work was conducted. The views expressed are those of the authors and not necessarily those of NIHR, the NHS, or the UK Government Department of Health. A .K. is now formally based at the Liverpool School of Tropical Medicine.

Declaration of interests

All authors declare no conflicts of interest.

Data sharing statement

Statistical code is available upon request to the corresponding author; no other additional data are available.

References

1. Gibson G, Loddenkemper R, Sibille Y, et al. The European Lung White Book. 1st ed: European Respiratory Society 2013.
2. Peters R, Poulter R, Warner J, et al. Smoking, dementia and cognitive decline in the elderly, a systematic review. *BMC geriatrics* 2008;8:36. doi: 10.1186/1471-2318-8-36
3. Gellert C, Schottker B, Brenner H. Smoking and all-cause mortality in older people: systematic review and meta-analysis. *Archives of internal medicine* 2012;172(11):837-44. doi: 10.1001/archinternmed.2012.1397
4. GHK. *A study on liability & the health costs of smoking DG SANCO (2008/C6/046) updated final report* http://ec.europa.eu/health//sites/health/files/tobacco/docs/tobacco_liability_final_en.pdf (accessed December 13, 2016).
5. Goodchild M, Nargis N, Tursan d'Espaignet E. Global economic cost of smoking-attributable diseases. *Tobacco Control* 2017 doi: 10.1136/tobaccocontrol-2016-053305
6. World Health Organization. *Global status report on alcohol and health 2014.* 2014. http://apps.who.int/iris/bitstream/10665/112736/1/9789240692763_eng.pdf.
7. Organization WH. Alcohol, Fact Sheet: World Health Organization.
8. Substance Abuse and Mental Health Services Administration (US). *Center for Substance Abuse Treatment. Substance Abuse Among Older Adults. Treatment Improvement Protocol (TIP) Series, No. 26.* 1998. https://www.ncbi.nlm.nih.gov/books/NBK64419/.
9. The Royal College of Psychiatrists. *Alcohol and Older People* http://www.rcpsych.ac.uk/healthadvice/problemsdisorders/alcoholandolderpeople.aspx (accessed February 7, 2017).
10. Mukamal KJ, Kuller LH, Fitzpatrick AL, et al. Prospective study of alcohol consumption and risk of dementia in older adults. *Jama* 2003;289(11):1405-13.

For peer review only - http://bmjopen.bmj.com/site/about/guidelines.xhtml

11. Ronksley PE, Brien SE, Turner BJ, et al. Association of alcohol consumption with selected cardiovascular disease outcomes: a systematic review and meta-analysis. *BMJ* 2011;342 doi: 10.1136/bmj.d671
12. De Leon J, Rendon DM, Baca-Garcia E, et al. Association between smoking and alcohol use in the general population: stable and unstable odds ratios across two years in two different countries. *Alcohol and alcoholism (Oxford, Oxfordshire)* 2007;42(3):252-7. doi: 10.1093/alcalc/agm029
13. Stotts AL, Schmitz JM, Grabowski J. Concurrent treatment for alcohol and tobacco dependence: are patients ready to quit both? *Drug and alcohol dependence* 2003;69(1):1-7.
14. Xu WH, Zhang XL, Gao YT, et al. Joint effect of cigarette smoking and alcohol consumption on mortality. *Preventive medicine* 2007;45(4):313-9. doi: 10.1016/j.ypmed.2007.05.015
15. Hart CL, Davey Smith G, Gruer L, et al. The combined effect of smoking tobacco and drinking alcohol on cause-specific mortality: a 30 year cohort study. *BMC public health* 2010;10:789. doi: 10.1186/1471-2458-10-789
16. United Nations. *World Population Ageing 2015.* 2015. http://www.un.org/en/development/desa/population/publications/pdf/ageing/WPA2015_Report.pdf.
17. Shamseer L, Moher D, Clarke M, et al. Preferred reporting items for systematic review and meta-analysis protocols (PRISMA-P) 2015: elaboration and explanation. *BMJ* 2015;349:g7647. doi: 10.1136/bmj.g7647
18. Stroup DF, Berlin JA, Morton SC, et al. Meta-analysis of observational studies in epidemiology: A proposal for reporting. *JAMA* 2000;283(15):2008-12. doi: 10.1001/jama.283.15.2008
19. Daskalopoulou C, Stubbs B, Kralj C, et al. Physical activity and healthy ageing: A systematic review and meta-analysis of longitudinal cohort studies. *Ageing research reviews* 2017;38:6-17. doi: 10.1016/j.arr.2017.06.003
20. Hayden JA, van der Windt DA, Cartwright JL, et al. Assessing bias in studies of prognostic factors. *Annals of internal medicine* 2013;158(4):280-6. doi: 10.7326/0003-4819-158-4-201302190-00009
21. Larsson SC, Wallin A, Wolk A, et al. Differing association of alcohol consumption with different stroke types: a systematic review and meta-analysis. *BMC medicine* 2016;14(1):178. doi: 10.1186/s12916-016-0721-4
22. DerSimonian R, Laird N. Meta-analysis in clinical trials. *Controlled clinical trials* 1986;7(3):177-88.
23. Higgins JP, Thompson SG, Deeks JJ, et al. Measuring inconsistency in meta-analyses. *Bmj* 2003;327(7414):557-60. doi: 10.1136/bmj.327.7414.557
24. Sterne JA, Egger M, Smith GD. Systematic reviews in health care: Investigating and dealing with publication and other biases in meta-analysis. *BMJ* 2001;323(7304):101-5.
25. Egger M, Smith GD, Schneider M, et al. Bias in meta-analysis detected by a simple, graphical test. *BMJ* 1997;315(7109):629-34. doi: 10.1136/bmj.315.7109.629
26. Duval S, Tweedie R. Trim and fill: A simple funnel-plot-based method of testing and adjusting for publication bias in meta-analysis. *Biometrics* 2000;56(2):455-63.
27. Andrews G, Clark M, Luszcz M. Successful Aging in the Australian Longitudinal Study of Aging: Applying the MacArthur Model Cross–Nationally. *Journal of Social Issues* 2002;58(4):749-65. doi: 10.1111/1540-4560.00288
28. Bell CL, Chen R, Masaki K, et al. Late-life factors associated with healthy aging in older men. *Journal of the American Geriatrics Society* 2014;62(5):880-8. doi: 10.1111/jgs.12796
29. Britton A, Shipley M, Singh-Manoux A, et al. Successful aging: the contribution of early-life and midlife risk factors. *Journal of the American Geriatrics Society* 2008;56(6):1098-105. doi: 10.1111/j.1532-5415.2008.01740.x
30. Burke GL, Arnold AM, Bild DE, et al. Factors associated with healthy aging: the cardiovascular health study. *Journal of the American Geriatrics Society* 2001;49(3):254-62.
31. Ford AB, Haug MR, Stange KC, et al. Sustained personal autonomy: a measure of successful aging. *Journal of aging and health* 2000;12(4):470-89. doi: 10.1177/089826430001200402

For peer review only - http://bmjopen.bmj.com/site/about/guidelines.xhtml

32. Gu D, Zhang Z, Zeng Y. Access to healthcare services makes a difference in healthy longevity among older Chinese adults. *Social science & medicine (1982)* 2009;68(2):210-9. doi: 10.1016/j.socscimed.2008.10.025
33. Guralnik JM, Kaplan GA. Predictors of healthy aging: prospective evidence from the Alameda County study. *American Journal of Public Health* 1989;79(6):703-8.
34. Gureje O, Oladeji BD, Abiona T, et al. Profile and determinants of successful aging in the Ibadan Study of Ageing. *Journal of the American Geriatrics Society* 2014;62(5):836-42. doi: 10.1111/jgs.12802
35. Hamer M, Lavoie KL, Bacon SL. Taking up physical activity in later life and healthy ageing: the English longitudinal study of ageing. *British journal of sports medicine* 2014;48(3):239-43. doi: 10.1136/bjsports-2013-092993
36. Hodge AM, English DR, Giles GG, et al. Social connectedness and predictors of successful ageing. *Maturitas* 2013;75(4):361-6. doi: 10.1016/j.maturitas.2013.05.002
37. Hodge AM, O'Dea K, English DR, et al. Dietary patterns as predictors of successful ageing. *The journal of nutrition, health & aging* 2014;18(3):221-7. doi: 10.1007/s12603-013-0405-0
38. Kaplan MS, Huguet N, Orpana H, et al. Prevalence and Factors Associated With Thriving in Older Adulthood: A 10-Year Population-Based Study. *The journals of gerontology Series A, Biological sciences and medical sciences* 2008;63(10):1097-104.
39. LaCroix AZ, Rillamas-Sun E, Woods NF, et al. Aging Well Among Women Veterans Compared With Non-Veterans in the Women's Health Initiative. *The Gerontologist* 2016;56 Suppl 1:S14-26. doi: 10.1093/geront/gnv124
40. Li C, Zhang M, He Y, et al. Impact of health behavior on successful aging: A 5-year follow-up study among community elderly. *Chinese Mental Health Journal* 2001(15):324-26 (in Chinese).
41. Liu LF, Su PF. What factors influence healthy aging? A person-centered approach among older adults in Taiwan. *Geriatrics & gerontology international* 2017;17(5):697-707. doi: 10.1111/ggi.12774
42. Newman AB, Arnold AM, Naydeck BL, et al. "Successful aging": effect of subclinical cardiovascular disease. *Archives of internal medicine* 2003;163(19):2315-22. doi: 10.1001/archinte.163.19.2315
43. Newson RS, Witteman JC, Franco OH, et al. Predicting survival and morbidity-free survival to very old age. *Age (Dordrecht, Netherlands)* 2010;32(4):521-34. doi: 10.1007/s11357-010-9154-8
44. Pruchno RA, Wilson-Genderson M. A Longitudinal Examination of the Effects of Early Influences and Midlife Characteristics on Successful Aging. *The journals of gerontology Series B, Psychological sciences and social sciences* 2015;70(6):850-9. doi: 10.1093/geronb/gbu046
45. Reed DM, Foley DJ, White LR, et al. Predictors of healthy aging in men with high life expectancies. *American Journal of Public Health* 1998;88(10):1463-8.
46. Sabia S, Singh-Manoux A, Hagger-Johnson G, et al. Influence of individual and combined healthy behaviours on successful aging. *CMAJ : Canadian Medical Association journal = journal de l'Association medicale canadienne* 2012;184(18):1985-92. doi: 10.1503/cmaj.121080
47. Sarnak MJ, Katz R, Fried LF, et al. Cystatin C and aging success. *Archives of internal medicine* 2008;168(2):147-53. doi: 10.1001/archinternmed.2007.40
48. Shields M, Martel L. Healthy living among seniors. *Health reports* 2006;16 Suppl:7-20.
49. Sun Q, Townsend MK, Okereke OI, et al. Alcohol Consumption at Midlife and Successful Ageing in Women: A Prospective Cohort Analysis in the Nurses' Health Study. *PLOS Medicine* 2011;8(9):e1001090. doi: 10.1371/journal.pmed.1001090
50. Tampubolon G. Trajectories of the healthy ageing phenotype among middle-aged and older Britons, 2004–2013. *Maturitas* 2016;88:9-15. doi: 10.1016/j.maturitas.2016.03.002
51. Terry DF, Pencina MJ, Vasan RS, et al. Cardiovascular risk factors predictive for survival and morbidity-free survival in the oldest-old Framingham Heart Study participants. *Journal of the American Geriatrics Society* 2005;53(11):1944-50. doi: 10.1111/j.1532-5415.2005.00465.x

For peer review only - http://bmjopen.bmj.com/site/about/guidelines.xhtml

52. Vaillant GE, Mukamal K. Successful aging. *The American journal of psychiatry* 2001;158(6):839-47. doi: 10.1176/appi.ajp.158.6.839
53. Vaillant GE, Western RJ. Healthy aging among inner-city men. *International psychogeriatrics* 2001;13(4):425-37.
54. Willcox BJ, He Q, Chen R, et al. Midlife risk factors and healthy survival in men. *Jama* 2006;296(19):2343-50. doi: 10.1001/jama.296.19.2343
55. Doll R, Peto R, Boreham J, et al. Mortality in relation to smoking: 50 years' observations on male British doctors. *BMJ* 2004;328(7455):1519. doi: 10.1136/bmj.38142.554479.AE
56. Depp CA, Jeste DV. Definitions and predictors of successful aging: a comprehensive review of larger quantitative studies. *The American journal of geriatric psychiatry : official journal of the American Association for Geriatric Psychiatry* 2006;14(1):6-20. doi: 10.1097/01.JGP.0000192501.03069.bc
57. Pruchno R, Hahn S, Wilson-Genderson M. Cigarette smokers, never-smokers, and transitions: implications for successful aging. *International journal of aging & human development* 2012;74(3):193-209. doi: 10.2190/AG.74.3.b
58. Lang I, Wallace RB, Huppert FA, et al. Moderate alcohol consumption in older adults is associated with better cognition and well-being than abstinence. *Age and ageing* 2007;36(3):256-61. doi: 10.1093/ageing/afm001
59. Chen LY, Hardy CL. Alcohol consumption and health status in older adults: a longitudinal analysis. *Journal of aging and health* 2009;21(6):824-47. doi: 10.1177/0898264309340688
60. Stockwell T, Greer A, Fillmore K, et al. How good is the science? *Bmj* 2012;344:e2276; author reply e94. doi: 10.1136/bmj.e2276.
61. International Alliance for Responsible Drinking. *Drinking Guidelines - Data by Country - Worldwide* 2016. http://www.iard.org/policy-tables/drinking-guidelines-general-population.
62. Institute of Alcohol Studies. *Older people and alcohol Factsheet* http://www.ias.org.uk/uploads/pdf/Factsheets/Alcohol%20and%20older%20people%20FS%20May%202013.pdf (accessed December 14, 2016).
63. NHS Health. *Alcohol and Ageing. Is alcohol a major threat to healthy ageing for the baby boomers?* http://lx.iriss.org.uk/sites/default/files/resources/Alcohol%20and%20ageing.pdf (accessed December 15, 2016).
64. Stockwell T, Zhao J, Panwar S, et al. Do "Moderate" Drinkers Have Reduced Mortality Risk? A Systematic Review and Meta-Analysis of Alcohol Consumption and All-Cause Mortality. *Journal of studies on alcohol and drugs* 2016;77(2):185-98. doi: 10.15288/jsad.2016.77.185
65. Reid MC, Boutros NN, O'Connor PG, et al. The health-related effects of alcohol use in older persons: a systematic review. *Substance abuse* 2002;23(3):149-64. doi: 10.1080/08897070209511485
66. Stockwell T, Zhao J, Macdonald S. Who under-reports their alcohol consumption in telephone surveys and by how much? An application of the 'yesterday method' in a national Canadian substance use survey. *Addiction (Abingdon, England)* 2014;109(10):1657-66. doi: 10.1111/add.12609
67. Chatterji S, Byles J, Cutler D, et al. Health, functioning, and disability in older adults—present status and future implications. *The Lancet* 2015;385(9967):563-75. doi: 10.1016/s0140-6736(14)61462-8
68. WHO.World Health Organization. *Ageing and health Factsheet* http://www.who.int/mediacentre/factsheets/fs404/en/ (accessed November 8, 2016).
69. World Health Organization. *WHO report on the global tobacco epidemic, 2008 : the MPOWER package.* 2008. http://apps.who.int/iris/bitstream/10665/43818/1/9789241596282_eng.pdf.
70. World Health Organization. *Tobacco Factsheet* http://www.who.int/mediacentre/factsheets/fs339/en (accessed December 9, 2016).

For peer review only - http://bmjopen.bmj.com/site/about/guidelines.xhtml

Table 1: Baseline characteristics of the eligible studies

Studies/Authors	Country	Panel	Data collection period	Follow-up*	Sample Size	Gender	Baseline Age
Andrews et al., 2002[27]	Australia	Australian Longitudinal Study of Aging (ALSA)	1992	8	1,403	55% men	> 70
Bell et al., 2014[28]	USA	Honolulu Heart Program (HHP)	1991 - 1993	up to 21 years	1,292	100% men	71-82
Britton et al., 2008[29]	England	Whitehall II study	1985 - 1988	17	5,823	71% men	35 - 55
Burke et al., 2001[30]	USA	Cardiovascular Health Study (CHS)	1989 - 1990, 1992 - 1993	6.5 and 3.5	3,342	39% men	> 65
Ford et al., 2000[31]	USA	N/A	1993	2	602	33% men	> 70
Gu et al., 2009[32]	China	Chinese Longitudinal Healthy Longevity Survey (CLHLS)	2002	3	15,972	45% men	65 - 109
Guralnik & Kaplan, 1989[33]	USA	Alameda County Study	1965	19	841	N/A	46 - 70
Gureje et al., 2014[34]	Nigeria	Ibadan Study of Ageing (ISA)	Aug 2003 - Nov 2004	5.3	930	61% men	> 65
Hamer et al., 2013[35]	England	English Longitudinal Study of Ageing (ELSA)	2002 - 2003	8	3,454	42% men	63.7 (M)
Hodge, English et al., 2013[36]	Australia	Melbourne Collaborative Cohort Study	1990 - 1994	11.7	5,512	37% men	63 (M)
Hodge, O'Dea et al., 2014[37]	Australia	Melbourne Collaborative Cohort Study	1990 - 1994	11.1 (WM)	6,309	39% men	64.1 (WM)
Kaplan et al., 2008[38]	Canada	Canadian National Population Health Survey (NPHS)	1994 - 1995	10	2,432	44% men	65 - 85
LaCroix et al., 2016[39]	USA	Women's Health Initiative (WHI)	1993 - 1998	16	68,153	100% women	68.9 (WM)
Li et al., 2001[40]	China	Shanghai Mental Health Center	1987	5	3,024	43% men	67.3 (M)
Liu & Su, 2016[41]	Taiwan	Taiwan Longitudinal Study on Aging (TLSA)	1993	14	3,155	56% men	71.7 (M)

For peer review only

For peer review only - http://bmjopen.bmj.com/site/about/guidelines.xhtml

Newman et al., 2003[42]	USA	Cardiovascular Health Study (CHS)	1989 - 1990, 1992 - 1993	8	2,932	39% men	71.9 (WM)
Newson et al., 2010[43]	Netherlands	Rotterdam Study	1990 - 1993	7.9	2,008	34% men	75.8 (M)
Pruchno & Wilson-Genderson, 2015[44]	USA	ORANJ BOWL: Ongoing Research on Aging in New Jersey: Bettering Opportunities for Wellness in Life	2006 -2008	4	2,614	37% men	60.5 (WM)
Reed et al., 1998[45]	USA	Honolulu Heart Program (HHP)	1965 - 1968	28	8,006	100% men	45 - 68
Sabia et al., 2012[46]	England	Whitehall II study	1991 - 1994	>16.3(Med)	5,100	70% men	51.3 (M)
Sarnak et al., 2008[47]	USA	Cardiovascular Health Study (CHS)	1989-1990 and 1992-1993 (African-American cohort)	4.3	2,140	38% men	74
Shields & Martel, 2006[48]	Canada	National Population Health Survey (NPHS)	1994-1995	8	1,309	N/A	> 65
Sun et al., 2011[49]	USA	Nurses' Health Study (NHS)	1980-1986	20 and 16	13,894	100% women	58 (Med)
Tampubolon, 2016[50]	England	English Longitudinal Study of Ageing (ELSA)	2004	9	14,765	46% men	50 - 89
Terry et al., 2005[51]	USA	Framingham Heart Study (FHS)	1948-1971	45	2,531	44% men	40 - 50
Vaillant & Mukamal, 2001[52]	USA	Study of Adult Development at Harvard University	circa 1940	until 60 or death	724	100% men	born mainly in the 1920s
Vaillant & Western, 2001[53]	USA	Study of Adult Development	1940 - 1945	60 y. (until 70 years or death)	456	100% men	14
Willcox et al., 2006[54]	USA	Honolulu Heart Program/Honolulu Asia Aging Study (HHP/HAAS)	1965-1968	40	5,820	100% men	54 (WM)

*: mean years, unless otherwise specified; M:mean; WM: Weighted Mean; Med: Median

For peer review only

Table 2: Aggregated Results of Smoking and Alcohol Consumption to Healthy Ageing

Authors	Smoking Variable	Statistics and 95%CI	Alcohol Variable	Statistics and 95%CI	Confounders
Andrews et al., 2002[27]	Past smoker (Yes or No)	no statistically significant (N/A)	-	-	Sociodemographic, Economic
Bell et al., 2014[28]	Past, Current, Never	Unhealthy vs Healthy Survival; OR, 95%CI<br>Past: 1.03, (0.78-1.35)<br>Current: 1.99, (1.06-3.75)<br>Never: Ref	Never (0 ounces/month);<br>Moderate to Heavy (>15 ounces/month, >1 drink per day);<br>Mild (>0–15 ounces/month, ≤1 drink per day)	Unhealthy vs Healthy Survival; OR, 95%CI<br>Never: 1.10, (0.81-1.50)<br>Moderate to Heavy: 1.04, (0.73-1.47)<br>Mild: Ref	Age
Britton et al., 2008[29]	Never, Ex-smoker, Current	OR, 95%CI, Men:<br>Never: 2.7, (1.8-4.1), Not SEP adj: 3.2, (2.1-4.7)<br>Ex-smoker: 2.5, (1.6-3.7), Not SEP adj: 2.6, (1.8-4.0)<br>Current smoker: Ref<br>Women:<br>Never: 2.2, (1.3-3.7), Not SEP adj: 2.4, (1.5-3.9)<br>Ex-smoker: 2.2, (1.3-3.7), Not SEP adj: 2.6, (1.5-4.5)<br>Current smoker: Ref	Alcohol consumption in the previous week (1 Unit = 8 g ethanol).<br>0 unit/week, 1-14 units/week, 15 units/week	OR, 95%CI, Men:<br>0: 1.2, (0.9-1.8), Not SEP adj: 1.0, (0.7-1.4)<br>1-14: 1.0, (0.8-1.2), Not SEP adj: 1.0, (0.8-1.2)<br>15: Ref<br>Women<br>0: 0.5, (0.3-0.9), Not SEP adj: 0.3, (0.2-0.5)<br>1-14: 1.0, (0.7-1.5), Not SEP adj: 0.8, (0.5-1.1)<br>15: Ref	Sociodemographic, Economic, Model Characteristics

For peer review only - http://bmjopen.bmj.com/site/about/guidelines.xhtml

Burke et al., 2001[30]	Former, Current, Never	Proportion of HY, 95%CI:<br>Model with Behavioural Factors only<br>Former: 0.75, N/A<br>Current: 0.56, N/A<br>Never: Ref<br>Model with Behavioural Factors & Subclinical Disease Factors<br>Former: 0.78, (0.69, 0.88)<br>Current: 0.66, (0.56, 0.78)<br>Never: Ref	Wine Drink: Yes, No	Proportion of HY, 95%CI:<br>Model with Behavioural Factors only<br>Wine Drink: 1.11, (0.99, 1.24)<br>Model with Behavioural Factors & Subclinical Disease Factors<br>Wine Drink: 1.04, (0.93, 1.17)	Sociodemographic, Economic, Health Behaviour, Diseases and Physical Measurements
Ford et al., 2000[31]	Current Smoker: Yes, No	OR, 95%CI:<br>No: 2.14, (1.02-4.48)<br>Yes: Ref	Current drinker (wine, beer, or liquor): Yes, No	OR, 95%CI:<br>No: 1.00, (0.59-1.71)	Sociodemographic, Economic, Health Behaviour, Diseases and Physical Measurements, Attitude and Social Environment
Gu et al., 2009[32]	Current Smoker: Yes, No	OR, 95% CI of access to healthcare at present & in childhood on healthy survival<br>Model I: 1.05, (0.91-1.21)<br>Model II: 1.00, (0.86-1.16)<br>Model III: 1.00, (0.86-1.16)<br>No: Ref	Current Drinker: Yes, No	OR, 95% CI of access to healthcare at present & in childhood on healthy survival<br>Model I: 1.21, (1.05-1.39)<br>Model II: 1.11, (0.96-1.28)<br>Model III: 1.10, (0.96-1.27)<br>No: Ref	Model I: Sociodemographic, Economic<br>Model II: Sociodemographic, Economic, Attitude and Social Environment<br>Model III: Sociodemographic, Economic, Attitude and Social Environment, Model Characteristics

For peer review only - http://bmjopen.bmj.com/site/about/guidelines.xhtml

Guralnik & Kaplan, 1989[33]	Past-Never, Current	OR, 95%CI<br>High function vs all others (Dead or Lower/Moderate)<br>Health Practices Model: Past-Never: 2.8, (1.6-4.8), Current: Ref<br>Combined Model: Past-Never: 3.0, (1.8-5.1), Current: Ref<br><br>High function vs Deceased<br>Past-Never: 6.1, (3.3-8.3), Current: Ref<br>High function vs Low/Moderate<br>Past-Never: 2.2, (1.3, 3.8), Current: Ref	None, 1-60, >60 (drinks/month)	OR, 95%CI<br>High function vs all others (Dead or Lower/Moderate)<br>Health Practices Model: 1-60 vs None: 2.4, (1.2-4.8)<br>1-60 vs >60: 1.7, (0.6-5.0)<br>Combined Model: 1-60 vs None: 2.1, (1.1-4.1), 1-60 vs >60: 1.7, (0.6-5.1)<br><br>High function vs Deceased<br>1-60 vs None: 3.1, (1.5-6.5), 1-60 vs >60: 2.5, (0.7-8.2)<br>High function vs Low/Moderate<br>1-60 vs None: 2.2, (1.1-4.4)<br>1-60 vs >60: 1.3, (0.4-4.0)	Health Practices Model: Health Behaviour, Physical Measurement<br>Combined Model: Sociodemographic, Economic, Diseases, Physical Measurement, Health Behaviour
Gureje et al., 2014[34]	Ever smoking: Yes, No	OR, 95%CI<br>Total: No: 1.6, (0.71-3.46), Yes: Ref<br>Male: No: 4.7, (1.61-14.52), Yes: Ref<br>Female: No: 0.4, (0.22, 1.13), Yes: Ref	Ever having alcohol: Yes, No	OR, 95%CI<br>Total: No: 1.0, (0.51, 2.10), Yes: Ref<br>Male: No: 0.5, (0.10, 2.73), Yes: Ref<br>Female: No: 0.3, (0.47, 2.18), Yes: Ref	Sociodemographic, Economic, Health Behaviour, Diseases and Physical Measurements, Attitude and Social Environment
Hamer et al., 2013[35]	Current, Ex/Non-smoker	OR, 95%CI<br>Current: 0.66, (0.48-0.89)<br>Ex/Non-smokers: Ref	Daily, at least Weekly, Rarely, Never	-	Sociodemographic, Economic, Health Behaviour

For peer review only - http://bmjopen.bmj.com/site/about/guidelines.xhtml

Hodge, English et al., 2013[36]	Former, Current, Never	OR, 95% CI<br>Former: 0.72, (0.61, 0.84)<br>Current: 0.45, (0.32, 0.65)<br>Never: Ref	Alcohol intake (g/day)	OR, 95%CI<br>1–20: 1.06, (0.90-1.24)<br>21–40: 1.16, (0.93-1.46)<br>41–60: 1.02, (0.72-1.45)<br>60+: 1.25, (0.79-1.97)<br>None: Ref	Sociodemographic, Economic, Health Behaviour, Diseases and Physical Measurements, Attitude and Social Environment
Hodge, O'Dea et al., 2014[37]	Former, Current, Never	OR, 95%CI<br>Model without BMI and WHR<br>Former: 0.67, (0.59-0.77)<br>Current: 0.39, (0.30-0.50)<br>Never: Ref<br><br>Model with BMI and WHR<br>Former: 0.69, (0.60-0.79)<br>Current: 0.38, (0.29-0.48)<br>Never: Ref	Alcohol intake (g/day)	OR, 95%CI<br>Model without BMI and WHR<br>1-20: 1.19, (1.03-1.36)<br>21-40: 1.37, (1.11-1.67)<br>41-60: 1.27, (0.94-1.74)<br>60+: 1.17, (0.78-1.76)<br>None: Ref<br><br>Model with BMI and WHR<br>1-20: 1.17, (1.02-1.34)<br>21-40: 1.33, (1.09-1.63)<br>41-60: 1.29, (0.95-1.76)<br>60: 1.20, (0.80-1.80)<br>None: Ref	Sociodemographic, Economic, Health Behaviour, Diseases and Physical Measurements, Model Characteristics
Kaplan et al., 2008[38]	Smoker, Never smoker	OR, 95%CI<br>Thrivers vs NonThrivers: Smoker: 1.89, (1.10-3.23), Never smoker: Ref<br>Thrivers vs Deceased: Smoker: 4.35, (2.44-7.69), Never smoker: Ref	Moderate (1-14 drinks/week), None or Heavy	OR, 95%CI<br>Thrivers vs NonThrivers, Moderate: 1.78, (1.07-2.95), None-Heavy: Ref<br>Thrivers vs Deceased, Moderate: 2.22, (1.25-3.95), None-Heavy: Ref	Sociodemographic, Economic, Attitude and Social Environment, Health Behaviour, Diseases and Physical Measurements

For peer review only - http://bmjopen.bmj.com/site/about/guidelines.xhtml

LaCroix et al., 2016[39]	Current, Past, Never	OR, 95%<br>Crude Model, Veterans<br>Current: 0.35, (0.22-0.57), Past: 0.85, (0.71-1.02), Never: Ref<br>Adjusted Model, Veterans<br>Current: 0.31, (0.19-0.52), Past: 0.80, (0.66-0.97), Never: Ref<br>Crude Model, Non-Veterans<br>Current: 0.49, (0.45-0.54), Past: 0.83, (0.80-0.86), Never: Ref<br>Adjusted Model, Non-Veterans<br>Current: 0.50, (0.46-0.55), Past: 0.77, (0.74-0.80), Never: Ref<br><br>Model for Veterans Only:<br>Crude: Current: 0.35, (0.22-0.57), Past: 0.85, (0.71-1.02), Never: Ref<br>Adjusted: Current: 0.31, (0.18-0.52), Past: 0.79, (0.65-0.95), Never: Ref	Drinker, Non-Drinker	OR, 95%<br>Crude Model, Veterans<br>Non-drinker: 0.59, (0.48-0.73), Drinker: Ref<br>Adj. Model, Veterans<br>Non-drinker: 0.64, (0.51-0.81), Drinker: Ref<br>Crude Model, Non-Veterans<br>Non-drinker: 0.67, (0.64-0.69), Drinker: Ref<br>Adj. Model, Non-Veterans<br>Non-drinker: 0.71, (0.68-0.74), Drinker: Ref<br><br>Model for Veterans Only:<br>Crude: Non-drinker: 0.59, (0.48-0.73), Drinker: Ref<br>Adj.: Non-drinker: 0.62, (0.49-0.78), Drinker: Ref	Crude: Age<br>Adj.: Sociodemographic, Economic, Model Characteristics, Health Behaviour, Diseases and Physical Measurements
Li et al., 2001[40]	Current, Former, Never	RR, 95%CI<br>Current: 1.11, (1.01-1.21)<br>Former: 0.87, (0.74-1.03)<br>Never: 0.96, (0.88-1.05)	Current, Former, Non-Drinker	RR, 95%CI<br>Current: 1.16, (1.07-1.27)<br>Former: 0.87, (0.68-1.12)<br>Never: 0.89, (0.81-0.96)	Sociodemographic, Health Behaviour
Liu & Su, 2016[41]	Smoking (Yes/ No)	β= 0.042, SE=0.03, p-value: 0.163<br>No: Ref	Alcohol (Yes/ No)	β=0.201, SE: 0.03, p-value < 0.001<br>No: Ref	Sociodemographic, Health Behaviour, Attitude & Social Environment

For peer review only - http://bmjopen.bmj.com/site/about/guidelines.xhtml

Newman et al., 2003[42]	Self-Reported Pack-years:<br>None<br>=<10<br>>10-20<br>>20-40<br>>40	Proportion of SY, 95% CI<br>Current Smoker: 0.77, (0.65-0.90)<br>(multiplication factor)<br>=<10 : 0.84 (0.73-0.98)<br>>10-20 : 0.90 (0.76-1.07)<br>>20-40 : 0.99 (0.85-1.15)<br>>40 : 0.82 (0.71-0.95)<br>None: Ref	-	-	Sociodemographic, Economic, Health Behaviour, Diseases and Physical Measurements
Newson et al., 2010[43]	Never, Former or Current	Survival Morbidity free vs non-survival, OR, 95% CI:<br>Age and sex-adjusted model: 1.38, (1.07, 1.77)<br>Full model: 1.26, (0.97, 1.65)	average consumption(gr)/day	not statistically significant	Sociodemographic, Economic, Health Behaviour, Diseases and Physical Measurements, Attitude & Social Environment
Pruchno & Wilson-Genderson, 2015[44]	Current Smoker (Yes, No)	Successful: Ref<br>Unsuccessful: b=0.55, SD=0.26, 95% CI=(1.03, 2.93), exp(b)=1.74<br>Subjective only: b=0.32, SD=0.25, 95% CI=(0.83, 2.27), exp(b)=1.38<br>Objective only: b=0.24, SD=0.23, 95% CI=(0.81, 1.99), exp(b)=1.27	Days per week where have at least one drink of alcohol (0-7)	Successful: Ref<br>Unsuccessful: b=-0.09, SD=0.08, 95% CI=(0.83, 1.00), exp(b)=0.91<br>Subjective only: b=-0.02, SD=0.04, 95% CI=(0.90, 1.06), exp(b)=0.98<br>Objective only: b=0.01, SD=0.03, 95% CI=(0.94, 1.08), exp(b)=1.01	Sociodemographic, Economic, Health Behaviour, Attitude & Social Environment
Reed et al., 1998[45]	Cigarette pack-years = (usual number of cigarettes/day) * (number of years)	OR, 95% CI<br>Healthy vs Illness With Impairment: 0.52, (0.39-0.70)<br>Healthy vs Illness Without Impairment: 0.46, (0.33-0.64)<br>Healthy vs Impairment Without Illness: -<br><br>Survival Ratio, 95%CI: 0.72, (0.65-0.79)	monthly intake of beer, wine, and liquor (ml of ethanol/day).	-	Sociodemographic, Health Behaviour, Diseases and Physical Measurement, Model Characteristics
Sabia et al., 2012[46]	Current or Former smoker, Never	OR, 95% CI<br>Never Smoker: 1.29, (1.11, 1.49)<br>Former or Current Smoker: Ref	Abstinence: no alcohol in the last week<br>Moderate: 1–14 units/wk women; 1–21 units/wk men	OR, 95% CI<br>Moderate : 1.31, (1.12, 1.53)<br>No Moderate: Ref	Sociodemographic, Economic, Health Behaviour

For peer review only

For peer review only - http://bmjopen.bmj.com/site/about/guidelines.xhtml

			Heavy: ≥15 units/wk women;<br>≥21 units/wk men.		
Sarnak et al., 2008[47]	Current, Former, Never	HR, 95% CI<br>Current: 1.73, (0.79, 1.80)<br>Former: 1.21, (0.92, 1.58)<br>Never: Ref	-	-	Sociodemographic, Diseases and Physical Measurements
Shields & Martel, 2006[48]	Current daily smokers, had quit daily smoking within the past 15 years, had quit at least 15 years ago or had never smoked every day	HR, 95% CI<br>Current: 0.7, (0.5, 1.0)<br>Quit during past 15 yrs: 0.7, (0.5, 0.9)<br>Never smoked/ Quit for 15+ years: Ref	Weekly/ Occasional drinker, Non-drinker	HR, 95% CI<br>Weekly/ Occasional drinker: 1.4, (1.1, 1.8)<br>Non-drinker: Ref	Sociodemographic, Economic, Health Behaviour, Attitude & Social Environment
Sun et al., 2011[49]	-	-	Alcohol Intake Levels (g/d)<br>i. 0<br>ii. =<5.0<br>iii. 5.1–15.0<br>iv. 15.1–30.0<br>v. 30.1–45.0	Fully adj. OR, 95% CI<br>i. 0 : Ref<br>ii. <=5.0 : 1.11 (0.96–1.29)<br>iii. 5.1–15.0 : 1.19 (1.01–1.40)<br>iv. 15.1–30.0: 1.28 (1.03–1.58)<br>v. 30.1–45.0 : 1.24 (0.87–1.76)<br><br>Age adj. OR, 95% CI<br>i. 0 : Ref<br>ii. <=5.0 : 1.12 (0.97–1.29)<br>iii. 5.1–15.0 : 1.22 (1.05–1.43)<br>iv. 15.1–30.0: 1.26 (1.03–1.53)<br>v. 30.1–45.0 : 1.05 (0.75–1.46)	Sociodemographic, Economic, Health Behaviour, Diseases and Physical Measurements

For peer review only

For peer review only - http://bmjopen.bmj.com/site/about/guidelines.xhtml

Tampubolon, 2016[50]	Current, No current	Baseline Model, Annual Rate of Phenotypic Decline: b: -0.679, 95%CI: (-0.852, -0.507) Gender Interaction Model, Annual Rate of Phenotypic Decline: b: -0.685, 95%CI: (−0.858,−0.513)	Drink daily Less than daily: Ref	Baseline Model, Annual Rate of Phenotypic Decline: b=0.414, 95%CI=(0.309, 0.519) Gender Interaction Model, Annual Rate of Phenotypic Decline: b=0.412, 95%CI=(0.307, 0.517)	Baseline Model: Sociodemographic, Economic, Health Behaviour, Diseases and Physical Measurements
Terry et al., 2005[51]	Present (smoked within the year before any baseline examination), Absent	OR, 95%CI: 0.51, (0.41, 0.63)	-	-	Sociodemographic, Economic, Health Behaviour, Diseases and Physical Measurement
Vaillant & Mukamal, 2001[52]	Smoking in < 30 pack-years (from age 15-50) Smoking in >= 30 pack-years (from age 15-50)	OR, 95%CI Happy-Well Men vs Sad-Sick or Prematurely Dead Smoking <30 pack-years: College Men at Age 75–80, 4.81, (0.84, 27.7) Smoking <30 pack-years: Core-City Men at Age 65–70, 4.56, (2.29, 9.11) Smoking in >=30 pack-years: Ref	Alcohol Abuse Absent (Yes, No)	OR, 95%CI Happy-Well Men vs Sad-Sick or Prematurely Dead No alcohol abuse: College Men at Age 75–80, - No alcohol abuse: Core-City Men at Age 65–70, 1.11, (0.53–2.35)	Sociodemographic, Diseases and Physical Measurement, Health Behaviour, Attitude and Social Environment
Vaillant & Western, 2001[53]	0-30 pack-years, > = 30 pack-years	OR, 95% CI Happy-Well vs Sad-Sick and Prematurely Dead Univariate Model: Smoking < 30 pack-years: 5.08 (2.85, 9.0) Multivariate Model: Smoking < 30 pack-years: 5.00 (2.38, 10.5)	Alcohol Abuse Absent (Yes, No)	OR, 95%CI Happy-Well vs Sad-Sick and Prematurely Dead Univariate Model: No Alcohol abuse: 2.64 (1.45, 4.81) Multivariate Model: No Alcohol abuse: 0.94 (0.72, 2.05).	Univariate: Diseases and Physical Measurements Multivariate: Sociodemographic, Economic, Health Behaviour, Diseases and Physical Measurement, Attitude and Social Environment

For peer review only - http://bmjopen.bmj.com/site/about/guidelines.xhtml

Willcox et al., 2006[54]	Ever smoker, Never smoker	OR, 95%CI<br>Usual Survival vs Exceptional Survival<br>Age-adj. : 1.27 (1.06-1.53),<br>Fully-adj. : 1.23 (1.01-1.50)<br>Never Smokers: Ref	3 or more drinks/day, less than 3 drinks/day	OR, 95%CI<br>Usual Survival vs Exceptional Survival<br>Age-adj. : 1.84 (1.29-2.62) ,<br>Fully-adj.: 1.61 (1.11-2.34)<br>Less than 3 drinks/d: Ref	Sociodemographic, Economic, Health Behaviour, Diseases and Physical Measurement

For peer review only

Table 3: Results of meta-analysis-Healthy Ageing compared to Smoking and Alcohol Consumption

Analysis	Number of Studies	ORs and 95%CI	p-value	I^2	Egger Bias; p-value	Trim-and-fill ORs and 95%CI	Number of Filled Studies
Never vs Current Smokers	7*	2.36 (2.03-2.75)	<.001	43.3%	1.42; .060	2.11 (1.82-2.45)	10
Never vs Former Smokers	5†	1.32 (1.23-1.41)	<.001	32.8%	-0.09; .935	unchanged	-
Past/Never vs Current	6‡	1.72 (1.20-2.47)	.003	87.2%	3.55; .142	1.20 (0.83-1.73)	9
Never vs Past/Current	5§	1.29 (1.16-1.43)	<.001	0.0%	1.08; .157	1.27 (1.14-1.40)	9
Drinkers vs Non-Drinkers	5‖	1.28 (1.08-1.52)	.004	72.1%	-1.25; .350	unchanged	-
Light vs Non-Drinkers	3¶	1.12 (1.03-1.22)	.010	0.0%	-8.17; .043	unchanged	-
Moderate vs Non- Drinkers	4**	1.35 (0.93-1.97)	.112	71.4%	1.77; .549	1.10 (0.77-1.57)	6
High to Non-Drinkers	3††	1.25 (1.03-1.44)	.002	0.0%	-0.63; .828	unchanged	-

OR: Odds Ratio; CI: Confidence Interval

* Bell et al., 2014[28]; Britton et al., 2008 (Males)[29]; Britton et al., 2008 (Females)[29]; Hodge, English et al., 2013[36]; Hodge, O'Dea et al., 2014[37]; LaCroix et al., 2016 (veterans)[39]; LaCroix et al., 2016 (non-veterans)[39]

† Bell et al., 2014[28]; Hodge, English et al., 2013[36]; Hodge, O'Dea et al., 2014[37]; LaCroix et al., 2016 (veterans)[39]; LaCroix et al., 2016 (non-veterans)[39]

‡ Ford et al., 2000[31]; Gu et al., 2009[32]; Guralnik & Kaplan, 1989[33]; Hamer et al., 2013[35]; Pruchno & Wilson-Genderson, 2015[44]; Terry et al., 2005[51]

§ Kaplan et al., 2008[38]; Newson et al., 2010[43]; Gureje et al., 2014[34]; Sabia et al., 2012[46]; Willcox et al., 2006[54]

|| Ford et al., 2000[31]; Gu et al, 2009[32]; Gureje et al., 2014[34]; LaCroix et al., 2016 (veterans)[39]; LaCroix et al., 2016 (non-veterans)[39]

¶ Hodge, English et al., 2013[36]; Hodge, O'Dea et al., 2014[37]; Sun et al., 2011[49]

** Britton et al., 2008 (Males)[29]; Britton et al., 2008 (Females)[29]; Guralnik & Kaplan, 1989[33]; Sun et al., 2011[49]

†† Hodge, English et al., 2013[36]; Hodge, O'Dea et al., 2014[37]; Sun et al., 2011[49]

For peer review only - http://bmjopen.bmj.com/site/about/guidelines.xhtml

For peer review only

For peer review only - http://bmjopen.bmj.com/site/about/guidelines.xhtml

PRISMA 2009 Flow Diagram

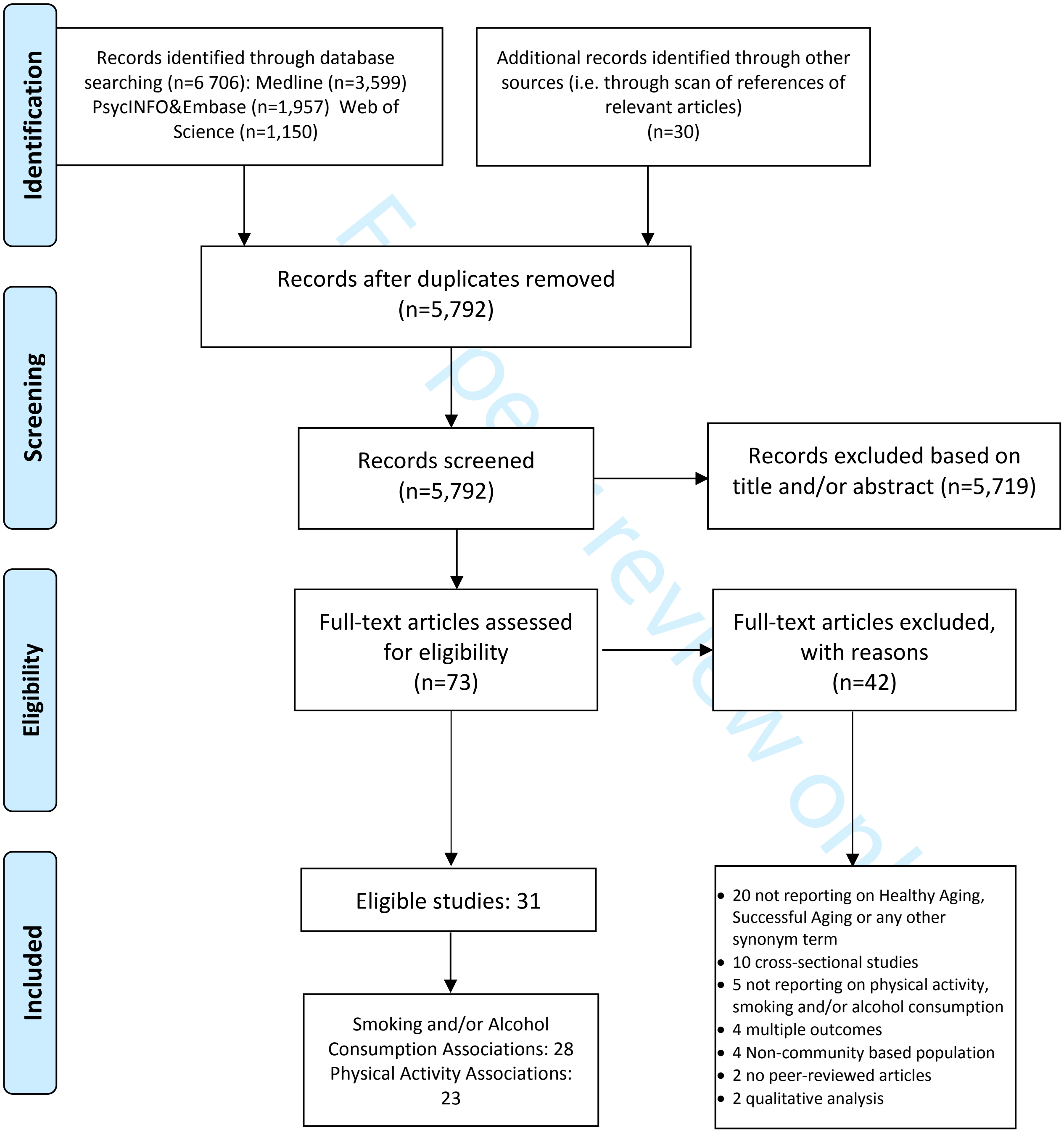

From: Moher D, Liberati A, Tetzlaff J, Altman DG, The PRISMA Group (2009). *P*referred *R*eporting *I*tems for *S*ystematic Reviews and *M*eta-*A*nalyses: The PRISMA Statement. PLoS Med 6(7): e1000097. doi:10.1371/journal.pmed1000097

For more information, visit www.prisma-statement.org.

For peer review only - http://bmjopen.bmj.com/site/about/guidelines.xhtml

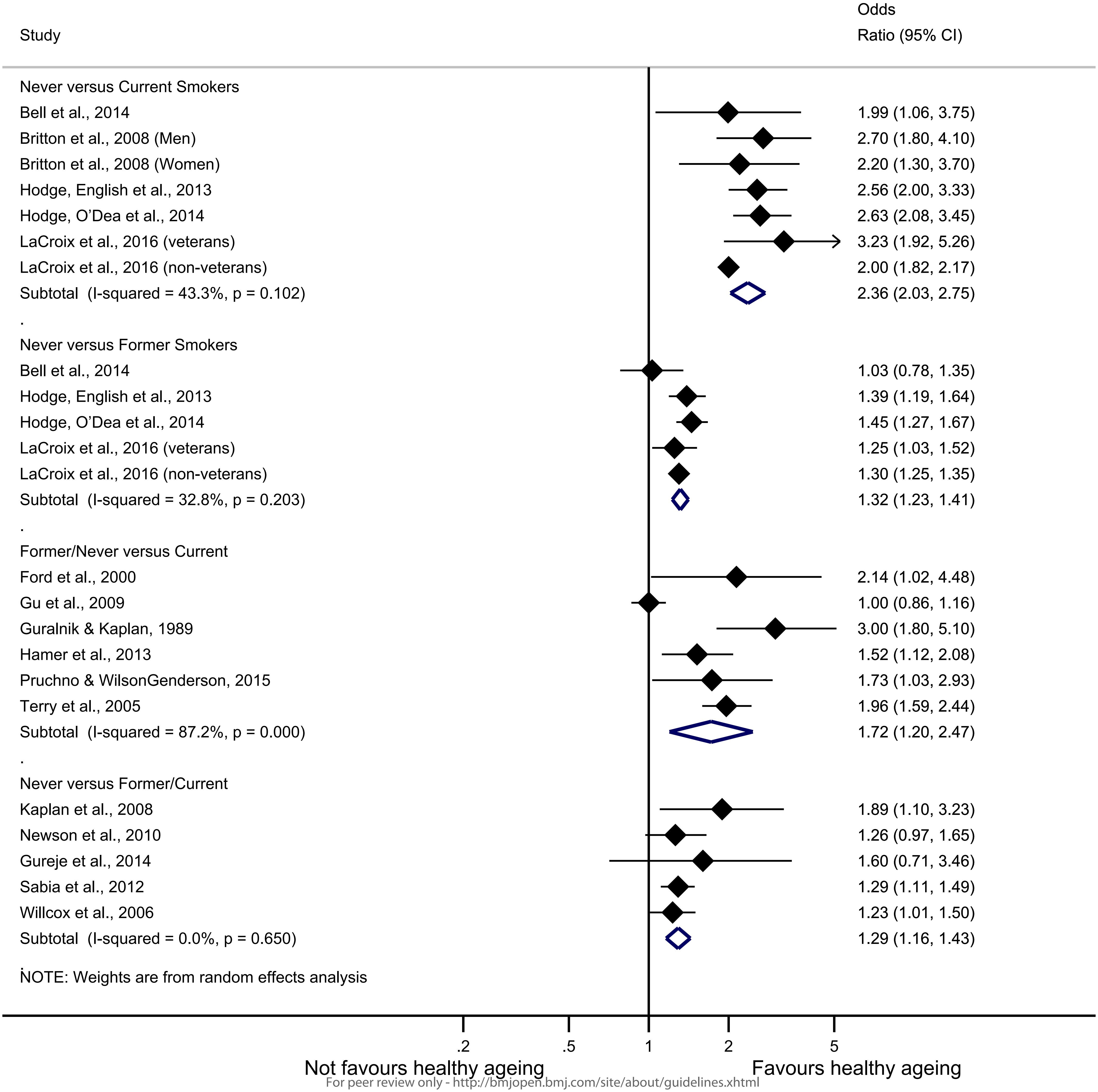
Study
Odds Ratio (95% CI)
Never versus Current Smokers
Bell et al., 2014 1.99 (1.06, 3.75)
Britton et al., 2008 (Men) 2.70 (1.80, 4.10)
Britton et al., 2008 (Women) 2.20 (1.30, 3.70)
Hodge, English et al., 2013 2.56 (2.00, 3.33)
Hodge, O'Dea et al., 2014 2.63 (2.08, 3.45)
LaCroix et al., 2016 (veterans) 3.23 (1.92, 5.26)
LaCroix et al., 2016 (non-veterans) 2.00 (1.82, 2.17)
Subtotal (I-squared = 43.3%, p = 0.102) 2.36 (2.03, 2.75)
.
Never versus Former Smokers
Bell et al., 2014 1.03 (0.78, 1.35)
Hodge, English et al., 2013 1.39 (1.19, 1.64)
Hodge, O'Dea et al., 2014 1.45 (1.27, 1.67)
LaCroix et al., 2016 (veterans) 1.25 (1.03, 1.52)
LaCroix et al., 2016 (non-veterans) 1.30 (1.25, 1.35)
Subtotal (I-squared = 32.8%, p = 0.203) 1.32 (1.23, 1.41)
.
Former/Never versus Current
Ford et al., 2000 2.14 (1.02, 4.48)
Gu et al., 2009 1.00 (0.86, 1.16)
Guralnik & Kaplan, 1989 3.00 (1.80, 5.10)
Hamer et al., 2013 1.52 (1.12, 2.08)
Pruchno & WilsonGenderson, 2015 1.73 (1.03, 2.93)
Terry et al., 2005 1.96 (1.59, 2.44)
Subtotal (I-squared = 87.2%, p = 0.000) 1.72 (1.20, 2.47)
.
Never versus Former/Current
Kaplan et al., 2008 1.89 (1.10, 3.23)
Newson et al., 2010 1.26 (0.97, 1.65)
Gureje et al., 2014 1.60 (0.71, 3.46)
Sabia et al., 2012 1.29 (1.11, 1.49)
Willcox et al., 2006 1.23 (1.01, 1.50)
Subtotal (I-squared = 0.0%, p = 0.650) 1.29 (1.16, 1.43)
.
NOTE: Weights are from random effects analysis
.2 .5 1 2 5
Not favours healthy ageing
Favours healthy ageing

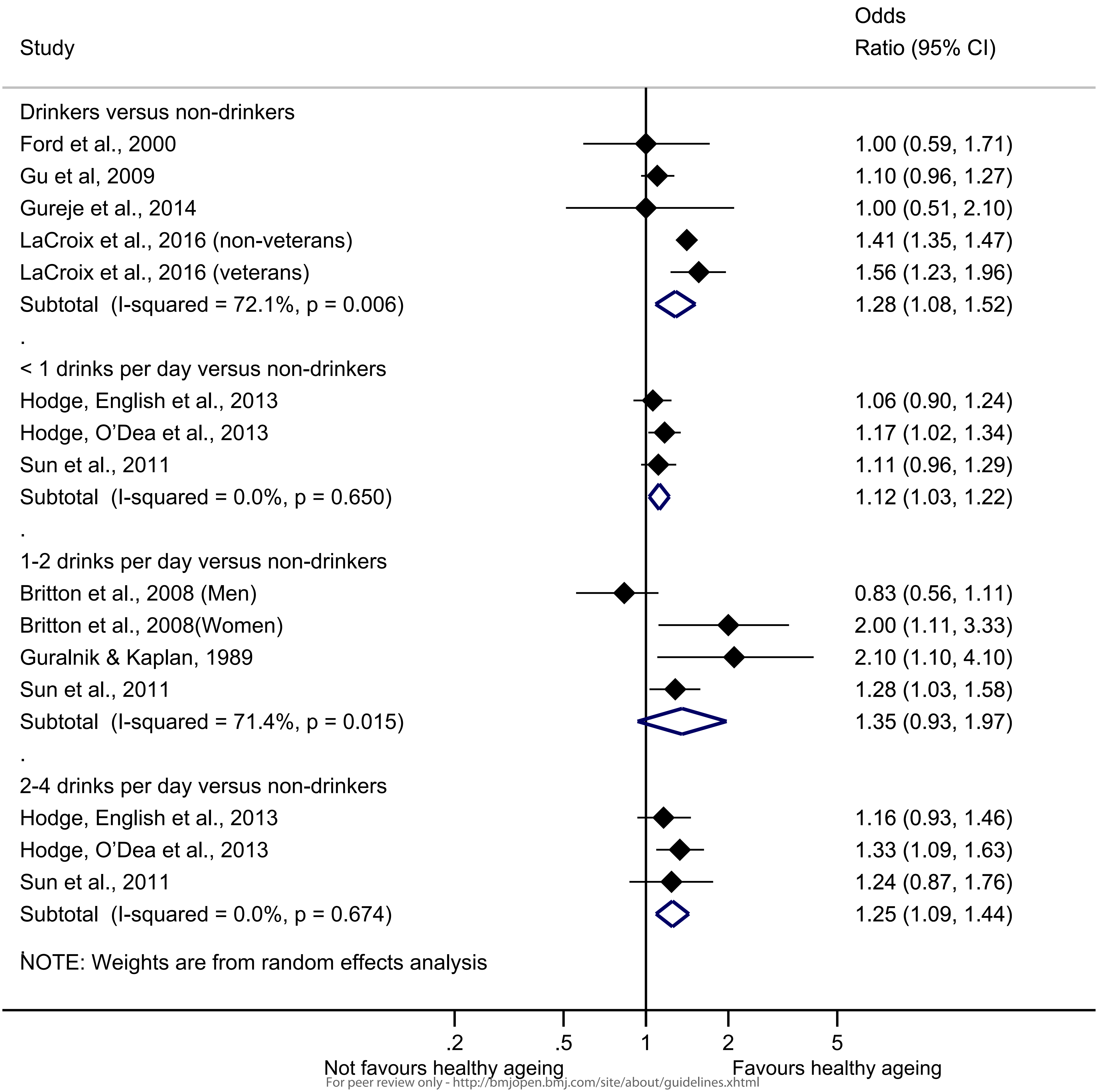
Study
Odds
Ratio (95% CI)
Drinkers versus non-drinkers
Ford et al., 2000 1.00 (0.59, 1.71)
Gu et al, 2009 1.10 (0.96, 1.27)
Gureje et al., 2014 1.00 (0.51, 2.10)
LaCroix et al., 2016 (non-veterans) 1.41 (1.35, 1.47)
LaCroix et al., 2016 (veterans) 1.56 (1.23, 1.96)
Subtotal (I-squared = 72.1%, p = 0.006) 1.28 (1.08, 1.52)
.
< 1 drinks per day versus non-drinkers
Hodge, English et al., 2013 1.06 (0.90, 1.24)
Hodge, O'Dea et al., 2013 1.17 (1.02, 1.34)
Sun et al., 2011 1.11 (0.96, 1.29)
Subtotal (I-squared = 0.0%, p = 0.650) 1.12 (1.03, 1.22)
.
1-2 drinks per day versus non-drinkers
Britton et al., 2008 (Men) 0.83 (0.56, 1.11)
Britton et al., 2008(Women) 2.00 (1.11, 3.33)
Guralnik & Kaplan, 1989 2.10 (1.10, 4.10)
Sun et al., 2011 1.28 (1.03, 1.58)
Subtotal (I-squared = 71.4%, p = 0.015) 1.35 (0.93, 1.97)
.
2-4 drinks per day versus non-drinkers
Hodge, English et al., 2013 1.16 (0.93, 1.46)
Hodge, O'Dea et al., 2013 1.33 (1.09, 1.63)
Sun et al., 2011 1.24 (0.87, 1.76)
Subtotal (I-squared = 0.0%, p = 0.674) 1.25 (1.09, 1.44)
NOTE: Weights are from random effects analysis
.2
.5
1
2
5
Not favours healthy ageing
Favours healthy ageing

MOOSE Checklist for Meta-analyses of Observational Studies

Item No	Recommendation	Reported on Page No
Reporting of background should include		
1	Problem definition	To address the need for more accurate estimates of the relationship between smoking and healthy ageing and alcohol consumption and healthy ageing (p. 6)
2	Hypothesis statement	In systematic reviews focusing on elderly people, smoking is associated with premature mortality and an increased incidence of Alzheimer's disease. Studies showed that light to moderate alcohol consumption is associated with a decrease in dementia and cognitive incidence, as well as with a reduced risk for cardiovascular disease mortality, incident coronary heart disease, incident stroke, stroke mortality and all-cause mortality(p. 5)
3	Description of study outcome(s)	The primary outcome of this review was health status measured by healthy ageing, and any other term related to it (e.g. successful ageing, active ageing, healthy survival etc.) (p. 7)
4	Type of exposure or intervention used	Eligible studies had to meet the following criteria: i. being published in an electronic journal article; ii. constitute an original peer-reviewed longitudinal study; and iii. report any kind of longitudinal association between smoking and/or alcohol consumption and healthy ageing (p. 7)
5	Type of study designs used	longitudinal study (p. 7)
6	Study population	Studies that included cohorts of individuals who were institutionalised or hospitalised and animal studies were also excluded (p. 7)
Reporting of search strategy should include		
7	Qualifications of searchers (eg, librarians and investigators)	Credentials are indicated in the title page
8	Search strategy, including time period included in the synthesis and key words	Search strategy and selection criteria (p.6-7) and Appendix 3.docx
9	Effort to include all available studies, including contact with authors	In case that full text could not be retrieved, the corresponding author of the paper was contacted (p. 7).<br>Other relevant systematic reviews of healthy ageing and reference lists of the eligible studies were also searched (p.7)
10	Databases and registries searched	MEDLINE (PubMed interface), EMBASE (OVID interface), Psychinfo (OVID interface) and CENTRAL (Cochrane Central Register of Controlled Trials) (p. 6-7)
11	Search software used, name and version, including special features used (eg, explosion)	EndNote (ENDNOTE X7, Thomson Reuters), (p. 7)
12	Use of hand searching (eg, reference lists of obtained articles)	Other relevant systematic reviews of healthy ageing and reference lists of the eligible studies have also been searched (p. 7)
13	List of citations located and those excluded, including justification	Details of the literature search are outlined in the PRISMA flow-chart: Figure 1. The citation list is available upon request.
14	Method of addressing articles published in languages other than English	Contributors (p. 18)
15	Method of handling abstracts and unpublished studies	exclusion of papers that were abstracts, conference papers, cross-sectional studies (p. 9-10)
16	Description of any contact with authors	we planned to contact authors for any missing data; however, this was not needed.

For peer review only

Reporting of methods should include		
17	Description of relevance or appropriateness of studies assembled for assessing the hypothesis to be tested	Search strategy and selection criteria paragraph (p. 6-7)
18	Rationale for the selection and coding of data (eg, sound clinical principles or convenience)	Data extraction and quality assessment paragraph (p. 8) and Table 1 and Table 2. We extracted data from each study that were relevant to the population, the exposure, the outcome and any confounder.
19	Documentation of how data were classified and coded (eg, multiple raters, blinding and interrater reliability)	Data extraction and quality assessment paragraph (p. 8).
20	Assessment of confounding (eg, comparability of cases and controls in studies where appropriate)	We extracted information regarding the different confounders that were used per study (Table 2)
21	Assessment of study quality, including blinding of quality assessors, stratification or regression on possible predictors of study results	Two investigators assessed study quality by using the QUIPS tool (Quality Assessment paragraph, p.11 and Table A2)
22	Assessment of heterogeneity	Heterogeneity was assessed by the I^2 (Data Analysis paragraph, p. 9)
23	Description of statistical methods (eg, complete description of fixed or random effects models, justification of whether the chosen models account for predictors of study results, dose-response models, or cumulative meta-analysis) in sufficient detail to be replicated	random effects meta-analysis was used (p. 9)
24	Provision of appropriate tables and graphics	We provided the PRISMA flow-chart, tables with the characteristics of studies, table with the results of meta-analysis and forest plots
Reporting of results should include		
25	Graphic summarizing individual study estimates and overall estimate	Figure 2 and Figure 3
26	Table giving descriptive information for each study included	Table 1 and Table 2
27	Results of sensitivity testing (eg, subgroup analysis)	Data did not allow to perform subgroup analysis
28	Indication of statistical uncertainty of findings	95% confidence intervals were presented with all estimators, I^2 values, and results of the trim-and-fill algorithm. Table 3.

Item No	Recommendation	Reported on Page No
Reporting of discussion should include		
29	Quantitative assessment of bias (eg, publication bias)	Publication bias was assessed Egger test and the trim-and-fill algorithm (Table 3)
30	Justification for exclusion (eg, exclusion of non-English language citations)	We did not exclude any study due to language. We excluded studies for the meta-analysis because they did not report a relevant statistic to OR or because alcohol conversion to comparable metrics was not possible
31	Assessment of quality of included studies	Data extraction and quality assessment paragraph (p. 8) and Table A2.
Reporting of conclusions should include		
32	Consideration of alternative explanations for observed results	Limitations are discussed in parag. Strengths and Limitations (p. 14). The most important are: adjustment by different use of covariates, different follow-up time and attrition rate, self-reported questionnaires and inconsistency in the way smoking/alcohol have been measured among the different cohorts.
33	Generalization of the conclusions (ie, appropriate for the data presented and within the domain of the literature review)	The positive impact of smoking abstinence and smoking cessation on healthy ageing is identified in both the majority of our primary studies and in our pooled effect estimate as well (even when we adjusted for publication bias). A positive relationship between limited

		consumption of drinking could also be argued but more research is needed.
34	Guidelines for future research	Conclusion parag. (p. 17)
35	Disclosure of funding source	Funding parag. (p.18)

From: Stroup DF, Berlin JA, Morton SC, et al, for the Meta-analysis Of Observational Studies in Epidemiology (MOOSE) Group. Meta-analysis of Observational Studies in Epidemiology. A Proposal for Reporting. *JAMA*. 2000;283(15):2008-2012. doi: 10.1001/jama.283.15.2008.
Transcribed from the original paper within the NEUROSURGERY® Editorial Office, Atlanta, GA, United Sates. August 2012.

For peer review only

UNIVERSITY of York
Centre for Reviews and Dissemination

NHS
National Institute for
Health Research

PROSPERO International prospective register of systematic reviews

ATHLOS: impact of physical activity, smoking and alcohol on healthy ageing throughout the lifespan: protocol for a systematic review

Christina Daskalopoulou, Matthew Prina, Martin Prince, Artemis Koukounari

Citation

Christina Daskalopoulou, Matthew Prina, Martin Prince, Artemis Koukounari. ATHLOS: impact of physical activity, smoking and alcohol on healthy ageing throughout the lifespan: protocol for a systematic review. PROSPERO 2016:CRD42016038130 Available from http://www.crd.york.ac.uk/PROSPERO_REBRANDING/display_record.asp?ID=CRD42016038130

Review question(s)

What is the impact of physical activity, smoking and alcohol consumption across the life span on healthy ageing?

Quantification and summary of the relationship between the aforementioned lifestyle factors and healthy ageing by using a meta-analytical approach.

Searches

The literature search will include any related term or synonym of healthy ageing and text words related to physical activity, smoking and alcohol. Where possible, the latter will be developed using medical subject headings (MeSH). We will search MEDLINE (PubMed interface), EMBASE (OVID interface), PsycINFO (OVID interface), Web of Science and CENTRAL (Cochrane Central Register of Controlled Trials).

In addition, scanning of the reference lists of the included studies will take place so as to identify any relevant studies that were not included by the initial research. Finally, authors of relevant studies will be contacted in case that supplementary information is required.

Types of study to be included

This review will be restricted to original studies (peer-reviewed).

Opinion pieces, books, letters, editorials, abstracts, dissertations/thesis and/or conference proceedings will not be considered.

Condition or domain being studied

Health status measured by healthy ageing.

Participants/ population

Eligible studies will include people, who are community-based, across the whole life span. Hospitalised or institutionalised populations will not been considered. Participants being disabled or diagnosed with dementia, frailty or any chronic disease will be included in the systematic review as long as they are community-based. There will be no other restrictions, for instance gender, age, education level, occupation and/or ethnicity.

Intervention(s), exposure(s)

Associations of either physical activity, smoking and/or alcohol consumption to one of the outcomes of healthy ageing, irrespectively of the way the latter is being measured/defined.

Comparator(s)/ control

Not applicable.

Context

Community-based populations and not clinical ones.

For peer review only - http://bmjopen.bmj.com/site/about/guidelines.xhtml

Outcome(s)

Primary outcomes

Health status measured by healthy ageing.

Secondary outcomes

Not applicable.

Data extraction, (selection and coding)

An EndNote (ENDNOTE X7, Thomson Reuters) library will be created so as to store all the studies retrieved by the electronic databases. Using EndNote's auto-deduplication function, duplicate citations will be removed. Since auto-deduplication is thought to be only partially successful (Qi et al., 2013), the remaining duplicates will be identified by hand-searching techniques. To do this, references will be alphabetically ordered according to the first authors' names and thereafter according to their titles.

Studies from the electronic database searches will be initially determined as relevant or not by title and abstract screening. References will be characterised as "Not Eligible" and "Potentially Eligible" by screening the title and the abstract of the study.

In order to include a study in the "Potential Eligible" folder the following criteria will be applied:

Study characteristics: "potentially eligible studies" have to be published in an electronic journal article and constitute an original peer-reviewed longitudinal study. We will include studies that examined healthy ageing as an outcome and that reported on the following determinants: physical activity, smoking and/or alcohol consumption.

Study participants: to be "potentially eligible", the title or abstract of the study must clearly state that the study population is an observational study/cohort study and that people are not hospitalised or belong to a specific clinical population. Participants being disabled or diagnosed with dementia, frailty or any chronic disease will be included in the systematic review as long as they are community based. There will be no other restrictions.

Interventions: To be "potentially eligible", the title or abstract must clearly state that the study reports the associations of either physical activity, smoking and/or alcohol consumption to one of the outcomes of healthy ageing, irrespectively of the way the latter is being measured/defined.

The final inclusion will be based on full text screening. Full text studies of "potentially eligible" references will be retrieved and assessed for their final inclusion by the first reviewer and a second researcher. A random sample of those, accounting for the 5% of the total studies, will be checked by a senior epidemiologist. Once the screening process is completed, the creation of a PRISMA flow diagram will be generated so as to illustrate the study selection process.

Risk of bias (quality) assessment

The risk of bias in primary studies will be assessed by using the Quality in Prognosis Studies (QUIPS) tool (Hayden et al., 2013). Validity and bias should be assessed by considering the following 6 areas: participation, attrition, prognostic factor measurement, confounding measurement and account, outcome measurement and analysis and reporting. During the first step, the 6 aforementioned potential sources of bias will be classified as either "yes", "partly", "no" or "unsure" by the first reviewer. Each study will then be characterised with an overall risk bias rating of "high", "moderate" or "low".

This characterisation will be defined as follows: "low" risk of bias will have these studies which fulfil all or the majority of the 6 QUIPS tool domains (no source of bias should be characterised as "yes" and at most one "partly"). "Moderate" risk of bias will have the studies that fullfil some of the aforementioned criteria; and "high" risk of bias will have those that meet few or none of the criteria.

During the next step, the first reviewer and the senior epidemiologist will judge if any of the "high" and "moderate" level studies present fatal flaws or considerable amount of bias and hence, in order to avoid reaching any misleading conclusion, it would be better to exclude these studies from the results (Sterne et al., 2001). The reasons based on which any studies have been excluded will be provided with full detail in a supplementary table.

For peer review only - http://bmjopen.bmj.com/site/about/guidelines.xhtml

Publication bias will be assessed for syntheses including five or more studies using a funnel plot and the Egger regression asymmetry test.

Strategy for data synthesis

The impact of physical activity, smoking and alcohol consumption on healthy ageing across the lifespan will be presented. In particular, the different levels of these factors and how these are related to healthy ageing will be extracted.

We will consider the different levels/measurements of these factors as statistically significant if i) the reported P-value is less than 0.05; ii) the author reported that there was a significant association; and iii) the 95% confidence interval around odds ratio or a similar statistic did not include 1.

Studies that refer no impact of the aforementioned determinants will also be presented. In addition, any concerns regarding the generalisability of the findings will be mentioned. For example, a small sample size, ethnicity/gender bias or important number of participants that were lost to follow-up will be referred.

Assuming non-substantial heterogeneity in study characteristics (e.g. determinant measurements, outcomes, etc.), we will carry out a meta-analysis to pool together effect estimates for each determinant. I-squared statistic will be used in order to assess the heterogeneity of the studies and based on this and the theoretical ground of the included studies, a fixed or random effect meta-analysis will be performed (Higgins et al., 2011). If a meta-analysis is not feasible due to amplified heterogeneity for the outcome and/or the lifestyle determinant measurement, a narrative synthesis for summarising the evidence will be performed.

In case of any discrepancies among the studies, study designs, populations, precision and publication bias will be assessed. Quality of our evidence will be considered as high when further research is very unlikely to change our confidence in the estimate of effect, moderate when further research is likely to have a considerable impact on our confidence and may change our estimate. The quality of evidence will be considered as low, in case that further research is very likely to have an important impact on our confidence in the estimate of effect, and is likely to change the estimate; and very low, when we are not certain about the estimate effect.

Quality of our findings will be assessed by performing a sensitivity analysis, should the selected studies permit it. For example, we will repeat the meta-analysis by excluding studies with low quality and see what will be the impact of it to our conclusions.

Analysis of subgroups or subsets

If the selected data permit, sub-group analyses will be carried out so as to stratify for different physical activity measurements (i.e subjective/self-report vs objective assessment of PA).

Dissemination plans

The findings of this review will be presented to relevant scientific conferences, for example ATHLOS consortium workshops, as well as to academic journals. Furthermore, conclusions will be disseminated through research and university networks.

Contact details for further information

Christina Daskalopoulou

King's College London, Institute of Psychiatry, Psychology & Neuroscience, Centre for Global Mental Health, Department of Health Services and Population Research, David Goldberg Centre, De Crespigny Park, Denmark Hill, London SE5 8AF, UK.

christina.daskalopoulou@kcl.ac.uk

Organisational affiliation of the review

King's College London, Institute of Psychiatry, Psychology & Neuroscience, Centre for Global Mental Health, Department of Health Services and Population Research

For peer review only - http://bmjopen.bmj.com/site/about/guidelines.xhtml

UNIVERSITY of York
Centre for Reviews and Dissemination

NHS
National Institute for Health Research

http://www.kcl.ac.uk/ioppn/depts/hspr/index.aspx

Review team

Ms Christina Daskalopoulou, King's College London
Dr Matthew Prina, King's College London
Professor Martin Prince, King's College London
Dr Artemis Koukounari, King's College London

Collaborators

Dr Carolina Kralj, King's College London

Anticipated or actual start date

14 March 2016

Anticipated completion date

31 December 2016

Funding sources/sponsors

Department of Health Services and Population Research, Institute of Psychology, Psychiatry and Neuroscience at King's College London is the Sponsor, meaning that it has overall control of the data. This project falls under the ATHLOS (Ageing Trajectories of Health: Longitudinal Opportunities and Synergies) project, funded by the European Union's Horizon 2020 Research and Innovation Programme under grant agreement number 63531.

Conflicts of interest

None known

Language

English

Country

England

Subject index terms status

Subject indexing assigned by CRD

Subject index terms

Age Factors; Aging; Alcohol Drinking; Exercise; Health; Health Status; Humans; Motor Activity; Risk Factors; Smoking

Reference and/or URL for protocol

http://www.crd.york.ac.uk/PROSPEROFILES/38130_PROTOCOL_20160326.pdf

Stage of review

Ongoing

Date of registration in PROSPERO

26 April 2016

Date of publication of this revision

26 April 2016

Stage of review at time of this submission	Started	Completed
Preliminary searches	Yes	No
Piloting of the study selection process	Yes	No

For peer review only - http://bmjopen.bmj.com/site/about/guidelines.xhtml

University of York
Centre for Reviews and Dissemination

NHS
National Institute for Health Research

Formal screening of search results against eligibility criteria	No	No
Data extraction	No	No
Risk of bias (quality) assessment	No	No
Data analysis	No	No

PROSPERO
International prospective register of systematic reviews

The information in this record has been provided by the named contact for this review. CRD has accepted this information in good faith and registered the review in PROSPERO. CRD bears no responsibility or liability for the content of this registration record, any associated files or external websites.

For peer review only

For peer review only - http://bmjopen.bmj.com/site/about/guidelines.xhtml

Systematic Review Protocol: ATHLOS: Impact of Physical Activity, Smoking and Alcohol on Healthy Ageing throughout the Life Span: protocol for a systematic review.

Embase, Psychinfo (OVID interface) and Cochrane Library

(health* ageing) OR (health* aging) OR (successful ageing) OR (successful aging) OR (positive ageing) OR (positive aging) OR (productive ageing) OR (productive aging) OR (ageing well) OR (aging well) OR (optim* ageing) OR (optim* aging) OR (unimpaired ageing) OR (unimpaired aging) OR (effective ageing) OR (effective aging) OR (robust ageing) OR (robust aging) OR (exceptional survival)

AND ((smoking) OR (tobacco) OR (cigarette*) OR (physical activity) OR (physical inactivity) OR (exercise*) OR (alcohol) OR (Alcohol Drinking))

MedLine search (PubMed/PMC)

(("health* ageing") OR ("health* aging") OR ("successful ageing") OR ("successful aging") OR ("positive ageing") OR ("positive aging") OR ("productive ageing") OR ("productive aging") OR ("ageing well") OR ("aging well") OR ("optim* ageing") OR ("optim* aging") OR ("unimpaired ageing") OR ("unimpaired aging") OR ("effective ageing") OR ("effective aging") OR ("robust ageing") OR ("robust aging") OR ("exceptional survival")) AND (("motor activity"[MeSH Terms]) OR ("smoking"[MeSH Terms]) OR ("Alcohol Drinking"[MesH Terms]))

For peer review only

Table A1: Areas of information included in the definition of healthy ageing

Authors	Survival	Health Status	Physical Performance	Diseases	Mental Health	Subjective Measurements	Others
Andrews et al., 2002			x		x		
Bell et al., 2014	x		x	x	x		
Britton et al., 2008		x	x	x	x		
Burke et al., 2001				x			
Ford et al., 2000							x
Gu et al., 2009	x		x				
Guralnik & Kaplan, 1989			x				
Gureje et al., 2014			x	x		x	
Hamer et al., 2013			x	x	x		
Hodge, English et al., 2013	x		x	x	x		
Hodge, O'Dea et al., 2014	x		x	x	x		
Kaplan et al., 2008		x	x				
LaCroix et al., 2016	x		x	x			
Li et al., 2001			x		x	x	
Liu & Su, 2016			x	x	x		
Newman et al., 2003			x	x	x		
Newson et al., 2010	x			x			
Pruchno & Wilson-Genderson, 2015			x	x		x	
Reed et al., 1998	x		x	x	x		
Sabia et al., 2012			x	x	x		
Sarnak et al., 2008			x	x	x		
Shields & Martel, 2006		x	x		x		
Sun et al., 2011	x		x	x	x		
Tampubolon, 2016							x
Terry et al., 2005	x			x	x		
Vaillant & Mukamal, 2001	x		x			x	x
Vaillant & Western, 2001	x		x			x	x
Willcox et al., 2006	x		x	x	x		
**Areas of information included in the definition of Healthy Ageing**	**12**	**3**	**23**	**18**	**16**	**5**	**4**

For peer review only

Table A2: Quality assessment of the studies – aggregated results of QUIPS tool

Author, Year of Publication	The study sample represents the population of interest on key characteristics, sufficient to limit potential bias of the observed relationship between PF and outcome.	Loss to follow-up is not associated with key characteristics sufficient to limit potential bias to the observed relationship between PF and outcome.	PF is adequately measured in study participants to sufficiently limit potential bias.	Outcome of interest is adequately measured in study participants to sufficiently limit potential bias.	Important potential confounders are appropriately accounted for, limiting potential bias with respect to the relationship between PF and outcome.	The statistical analysis is appropriate for the design of the study, limiting potential for presentation of invalid or spurious results.	Total
Andrews et al., 2002	Low Bias	Moderate Bias	Moderate Bias	Low Bias	Moderate Bias	Low Bias	Moderate Bias
Bell et al., 2014	Low Bias	Low Bias	Low Bias	Low Bias	Moderate Bias	Low Bias	Low Bias
Britton et al., 2008	Low Bias	Moderate Bias	Low Bias	Low Bias	Moderate Bias	Low Bias	Low Bias
Burke et al., 2001	Low Bias	Low Bias	Low Bias	Moderate Bias	Moderate Bias	Low Bias	Low Bias
Ford et al., 2000	Low Bias	Moderate Bias	Moderate Bias	Moderate Bias	Low Bias	Low Bias	Moderate Bias
Gu et al., 2009	Low Bias	Low Bias	Low Bias	Low Bias	Moderate Bias	Low Bias	Low Bias
Guralnik & Kaplan, 1989	Low Bias	Moderate Bias	Moderate Bias	Moderate Bias	Moderate Bias	Low Bias	Moderate Bias
Gureje et al., 2014	Low Bias	Moderate Bias	Low Bias	Low Bias	Moderate Bias	Low Bias	Low Bias
Hamer et al., 2013	Low Bias	Low Bias	Moderate Bias	Low Bias	Low Bias	Low Bias	Low Bias
Hodge, English et al., 2013	Low Bias	Moderate Bias	Low Bias	Low Bias	Low Bias	Low Bias	Low Bias
Hodge, O'Dea et al., 2014	Low Bias	High Bias	Low Bias	Low Bias	Low Bias	Low Bias	Moderate Bias
Kaplan et al., 2008	Low Bias	Moderate Bias	Low Bias	Moderate Bias	Low Bias	Low Bias	Low Bias
LaCroix et al., 2016	Low Bias	Moderate Bias	Low Bias	Low Bias	Moderate Bias	Low Bias	Low Bias
Li et al., 2001	Low Bias	High Bias	Low Bias	Low Bias	Moderate Bias	Low Bias	Moderate Bias
Liu & Su, 2016	Low Bias	Moderate Bias	Low Bias	Low Bias	Moderate Bias	Low Bias	Low Bias
Newman et al., 2003	Low Bias	Low Bias	Low Bias	Low Bias	Moderate Bias	Low Bias	Low Bias
Newson et al.,	Low Bias	Moderate Bias	Low Bias	Low Bias	Moderate Bias	Low Bias	Low Bias

For peer review only

2010							
Pruchno & Wilson-Genderson, 2015	Low Bias	Moderate Bias	Low Bias	Low Bias	Moderate Bias	Low Bias	Low Bias
Reed et al., 2011	Low Bias	Moderate Bias	Low Bias	Low Bias	Low Bias	Moderate Bias	Low Bias
Sabia et al., 2012	Low Bias	Low Bias	Low Bias	Moderate Bias	Low Bias	Low Bias	Low Bias
Sarnak et al., 2008	Low Bias	Moderate Bias	Moderate Bias	Low Bias	Moderate Bias	Low Bias	Moderate Bias
Shields & Martel, 2006	Low Bias	Moderate Bias	Low Bias	Low Bias	Low Bias	Low Bias	Low Bias
Sun et al., 2011	Low Bias	Low Bias	Low Bias	Low Bias	Low Bias	Low Bias	Low Bias
Tampubolon, 2016	Low Bias	Low Bias	Low Bias	Low Bias	Moderate Bias	Low Bias	Low Bias
Terry et al., 2005	Low Bias	Low Bias	Low Bias	Moderate Bias	Moderate Bias	Moderate Bias	Moderate Bias
Vaillant & Mukamal, 2001	High Bias	Moderate Bias	Low Bias	Moderate Bias	Moderate Bias	Low Bias	High Bias
Vaillant & Western, 2001	High Bias	Low Bias	Low Bias	Low Bias	Moderate Bias	Low Bias	Moderate Bias
Willcox et al., 2006	Moderate Bias	Moderate Bias	Low Bias	Low Bias	Moderate Bias	Low Bias	Moderate Bias

For peer review only

For peer review only

BMJ Open

Associations of Smoking and Alcohol Consumption with Healthy Ageing: A Systematic Review and Meta-analysis of longitudinal studies

Journal:	*BMJ Open*
Manuscript ID	bmjopen-2017-019540.R1
Article Type:	Research
Date Submitted by the Author:	27-Dec-2017
Complete List of Authors:	Daskalopoulou, Christina; King's College London, Health Service and Population Research Centre for Global Mental Health & Primary Care Research<br>Stubbs, B; King's College London, Health Service and Population Research Department; South London and Maudsley NHS Foundation Trust, Physiotherapy Department<br>Kralj, Carolina; King's College London, Health Service and Population Research Centre for Global Mental Health & Primary Care Research<br>Koukounari, Artemis; Liverpool School of Tropical Medicine, Department of Clinical Sciences<br>Prince, Martin; King's College London, Health Service and Population Research Centre for Global Mental Health & Primary Care Research<br>Prina, A.Matthew; King's College London, Health Service and Population Research Centre for Global Mental Health & Primary Care Research
**Primary Subject Heading**:	Public health
Secondary Subject Heading:	Global health, Health policy, Public health, Smoking and tobacco
Keywords:	healthy ageing, smoking, alcohol consumption, drinking, meta-analysis

SCHOLARONE™
Manuscripts

Associations of Smoking and Alcohol Consumption with Healthy Ageing: A Systematic Review and Meta-analysis of longitudinal studies

Christina Daskalopoulou, MSc[1], Brendon Stubbs, PhD[2,3], Carolina Kralj, MD[1], Artemis Koukounari, PhD[4], Martin Prince, MD, PhD[1], and Matthew A. Prina, PhD[2]

[*] Address for correspondence: Mrs C. Daskalopoulou, King's College London, Institute of Psychiatry, Psychology and Neuroscience, David Goldberg Centre, De Crespigny Park, London SE5 8AF, UK. (Email: christina.daskalopoulou@kcl.ac.uk.com)

[1] Health Service and Population Research Department, King's College London, Institute of Psychiatry, Psychology and Neuroscience, Centre for Global Mental Health, London, UK;

[2] Health Service and Population Research Department, King's College London, Institute of Psychiatry, Psychology and Neuroscience, London, UK;

[3] Physiotherapy Department, South London and Maudsley NHS Foundation Trust, London, UK;

[4] Department of Clinical Sciences, Liverpool School of Tropical Medicine, Liverpool, UK.

For peer review only - http://bmjopen.bmj.com/site/about/guidelines.xhtml

Abstract (298 words)

Objectives The number of older people is growing across the world, however quantitative synthesis of studies examining the impact of lifestyle factors on the ageing process are rare. We conducted a systematic review and meta-analysis of longitudinal studies to synthesise the associations of smoking and alcohol consumption with healthy ageing (HA).

Methods Major electronic databases were searched from inception to March 2017 (PROSPERO reg. CRD42016038130). Studies were assessed for methodological quality. Random-effect meta-analysis was performed to calculate pooled odds ratios (OR) and 95% confidence intervals (CI).

Results In total, we identified 28 studies (n=184,543); 27 studies reported results on smoking, 22 on alcohol consumption. 23 studies reported a significant positive association of never or former smoking with HA and 4 non-significant. 12 studies reported a significant positive association of alcohol consumption with HA, 9 no association, 1 negative. Meta-analysis revealed increased pooled OR of HA for never smokers compared to current smokers (2.36, 95%CI 2.03-2.75), never smokers compared to former smokers (1.32, 95%CI 1.23-1.41), former or never smokers compared to current smokers (1.72, 95%CI 1.20-2.47), never smokers compared to past or current smokers (1.29, 95%CI 1.16-1.43); drinkers compared to non-drinkers (1.28, 95%CI 1.08-1.52), light drinkers compared to non-drinkers (1.12, 95%CI 1.03-1.22), moderate drinkers compared to non-drinkers (1.35, 95%CI 0.93-1.97) and high drinkers compared to non-drinkers (1.25, 95%CI 1.09-1.44). There was considerable heterogeneity in the definition and measurement of HA and alcohol consumption.

Conclusions There is consistent evidence from longitudinal studies that smoking is negatively associated with HA. The associations of alcohol consumption with HA are

For peer review only - http://bmjopen.bmj.com/site/about/guidelines.xhtml

equivocal. Future research should focus on the implementation of a single metric of HA, on the use of consistent drinking assessment among studies and on a full-range of confounding adjustment. Our research also highlighted the limited research on ageing in low-and-middle income countries.

Key words: healthy ageing; smoking; alcohol consumption; drinking; meta-analysis.

For peer review only

For peer review only - http://bmjopen.bmj.com/site/about/guidelines.xhtml

Strengths and limitations of this study

- This is the first systematic review and meta-analysis trying to quantify the associations of smoking and alcohol consumption with healthy ageing; our findings were also adjusted for publication bias.
- Independent double screening, searching of previous systematic reviews in the field and the reference lists of the eligible studies as well as not excluding non-English studies guarantee that limited number of studies may have not been considered.
- Heterogeneity in the definition and measurement of healthy ageing as well as different confounding adjustment in the initial studies may have influenced our meta-analytical results.
- The number of studies included in the meta-analysis regarding the associations of alcohol consumption was small due to the great heterogeneity in the measurement of it.
- Our findings may not be generalisable due to the limited research in low and middle income countries.

For peer review only

Words: 3,975

Introduction

Smoking has emerged as the leading contributing factor of many respiratory diseases and one of the most important risk factors for cardiovascular diseases, cancers of several organs and many other pathological conditions.[1] In systematic reviews focusing on older people, smoking is associated with premature mortality[2] and an increased incidence of Alzheimer's disease.[3] Smoking is also linked with a considerable societal cost as smoking-related absenteeism and premature deaths create an additional burden to the healthcare system and to the economy in general.[4] The healthcare cost of tobacco-related diseases accounted for almost 6% of the global health expenditures in 2012.[5]

Tobacco use and harmful use of alcohol are recognised by the United Nations Political Declaration on Non-Communicable Diseases (NCDs) among the most common risk factors.[6] Harmful use of alcohol results approximately in 3.3 million deaths annually (5.9% of all deaths) and alcohol-attributable deaths are the highest for middle-aged people.[7] Older people's sensitivity to the effects of alcohol increases whereas tolerance decreases,[8] meaning that even a low amount of alcohol can create problems in an older person. In addition, older people who drink harmfully often do not present to services due to stigma associated with harmful drinking, and when they do, their symptoms can be easily confused with physical and mental issues associated with ageing.[9] Nevertheless, studies showed that light to moderate alcohol consumption is associated with a decreased risk in dementia,[10] as well as with a reduced risk for cardiovascular disease mortality, incident coronary heart disease, incident stroke, stroke mortality and all-cause mortality.[11]

Drinking and smoking are correlated in the general population[12] with smokers being more likely to drink and vice versa.[13] People who are smokers and heavy drinkers exhibit the highest mortality rate as indicated in a study of middle-aged male participants;[14] another

For peer review only - http://bmjopen.bmj.com/site/about/guidelines.xhtml

study also showed that men who smoked and drank more than 15 units per week of alcohol had almost three times higher all-cause mortality rate compared to non-smokers and non-drinkers.[15] As populations are ageing rapidly, with one in eight people currently aged 60 and over and with these estimates predicted to increase,[16] it is critical to understand the factors that will enable people to live longer and age in a healthy way.

To address the need for more accurate estimates of the relationship between smoking and healthy ageing and alcohol consumption and healthy ageing we conducted a systematic review and meta-analysis of longitudinal studies which examined the associations of these factors with the ageing process.

Methods

Search strategy and selection criteria

This systematic review has been written following the Preferred Reporting Items for a Systematic Review and Meta-Analysis (PRISMA) and the guidelines for Meta-Analyses Of Observational Studies in Epidemiology (MOOSE) to ensure accuracy and comprehensiveness.[17,18] (Appendix 1). A review protocol was prospectively registered in the international database of prospectively registered systematic reviews (PROSPERO) under registration number CRD42016038130 (Appendix 2). As part of a larger body of work considering modifiable lifestyle factors and healthy ageing, we originally planned to carry out a review focusing on the following: physical activity, smoking and alcohol consumption. The current systematic review specifically focuses on the findings related to smoking, alcohol consumption and healthy ageing outcomes since a sufficient amount of literature was identified on this topic alone. Our findings regarding healthy ageing and physical activity have been presented elsewhere.[19]

Two authors (C.D, C.K) searched MEDLINE (PubMed/PubMed Central interface), EMBASE (OVID interface), Psychinfo (OVID interface) and CENTRAL (Cochrane Central

For peer review only - http://bmjopen.bmj.com/site/about/guidelines.xhtml

Register of Controlled Trials) from inception up to April 2016. Searching methodology included any related term or synonym to healthy ageing and text word related to physical activity, smoking and alcohol consumption (Appendix 3). Other relevant systematic reviews and reference lists of the eligible studies were also searched. Finally, a second search was performed on 15th of March 2017 to include studies that were recently published.

Eligible studies had to meet the following criteria: i. being published in an electronic journal article; ii. constitute an original peer-reviewed longitudinal study; and iii. report any kind of longitudinal association between smoking and/or alcohol consumption and healthy ageing. Data on smoking and alcohol, through self-report and/or specific questionnaires, referred either to the current status and/or across the life span. The primary outcome of this review was health status measured by healthy ageing, and any other term related to it (e.g. successful ageing, active ageing, healthy survival etc.). Studies whose primary goal was the examination of a different determinant but included the aforementioned factors as covariates were also included. Due to the heterogeneity of the healthy ageing definition, studies reporting the latter as multiple outcomes or based solely on self-report were excluded. Studies that included cohorts of individuals who were institutionalised or hospitalised and animal studies were also excluded. No language restriction was applied.

In order to store the studies that were retrieved by the electronic search we used an EndNote (ENDNOTE X7, Thomson Reuters) library. After removal of duplicates, the two independent reviewers screened titles and abstracts of all potentially eligible papers. At the end of this procedure, any disagreement was solved by discussion. In case that an agreement could not be achieved, eligibility of the study was judged by discussion with a third senior researcher (A.M.P.). In case that full text could not be retrieved, the corresponding author of the paper was contacted via e-mail.

For peer review only - http://bmjopen.bmj.com/site/about/guidelines.xhtml

Data extraction and quality assessment

C.D. and C.K. independently extracted data from each study and a random sample of them was cross-checked by A.M.P. Setting/country of the study, data collection period, follow-up year, sample size, population, and baseline age information was recorded for each study. Definition and measurement of the healthy ageing outcome, of smoking and alcohol consumption were also recorded, as well as the odds ratios (OR) (or any other related statistic) and the 95% confidence intervals (CI). Reported statistics were extracted for the crude/unadjusted model and the most adjusted one reported in the study.

The methodological quality of the included studies was assessed by using the Quality in Prognosis Studies (QUIPS) tool. QUIPS evaluates six potential components of bias: inclusion, attrition, prognostic factor measurement, confounders, outcome measurement, and analysis and reporting.[20] During the application of the QUIPS tool smoking and/or alcohol consumption were considered as the only prognostic factors and all other variables, used as explanatory variables of the model, were considered as confounders. Since we included only longitudinal studies, the existence of attrition was expected. In the scenario that the attrition rate was high, authors' explanations were required in order to evaluate the study as bearing low risk of bias. Finally, the reliability of statistical models was evaluated according to the data presented; for instance, the reliability of studies that included results only for the statistically significant factors was judged with caution.

Data analysis

We performed meta-analyses in order to produce a pooled effect size estimate for the relationship between smoking and healthy ageing and alcohol consumption and healthy ageing. Separate meta-analyses were performed for the following categories:

For peer review only - http://bmjopen.bmj.com/site/about/guidelines.xhtml

Smoking: i) past and never smokers compared to current smokers; ii) former or never smokers compared to current smokers; iii) never smokers compared to former or current smokers.

Alcohol: For studies that provided alcohol quantities, we converted grams to drinks by assuming that 12 grams of alcohol are approximately one drink so as to have an alcohol consumption variable comparable among the studies.[21] In studies that provided alcohol consumption in an interval we took the middle of the interval as the most likely value. We created the following categories: light (< 1 drink/day), moderate (1–2 drinks/day), and high (> 2–4 drinks/day) consumption that were compared to never drinkers. Studies that did not report specific alcohol quantity but characterised the participants as drinkers or non-drinkers were examined separately.

We produced a pooled effect estimate of the aforementioned categories for the most adjusted models. In order to obtain results, at least three studies had to be included in the analysis. Due to the expected heterogeneity, a random effects meta-analysis using the DerSimonian-Laird model was performed.[22] If a study reported different results for men and women, both results were included, except in cases where a result for the mixed population was also provided; the same rationale was applied to cases that reported statistics per different sub-groups in the cohort. Heterogeneity was assessed with I^2 statistic for each analysis,[23] publication bias[24] was assessed with Egger bias test.[25] Finally, a trim-and-fill adjusted analysis was conducted to adjust for potential publication bias.[26] All analyses were performed using STATA 14 IC statistical software.

Results

The search identified 6,706 papers from the databases and 30 additional papers were obtained from other sources. After removing of duplicates and of papers that were conference papers, cross-sectional studies or animal studies, 73 papers were considered for full text-review. We

For peer review only - http://bmjopen.bmj.com/site/about/guidelines.xhtml

excluded 42 papers based on specific reasons after the full text-review and finally 28 studies are included in this report.[27-54] PRISMA flow diagram depicts the exact selection process **Figure 1**. Across the 28 eligible studies, there were 184,543 participants (almost 31% men), with sample size ranging from 456 to 68,153. Studies were published from 1989 to 2016 and half of them (14 out of 28) took place in USA. Studies were also conducted in England (four), in Australia (three), in China (two), in Canada (two), in Nigeria (one), in the Netherlands (one) and in Taiwan (one). Baseline age ranged from 14 to 109 years old and follow-up time from two years until death (>70 years old). Five studies examined men only and two studies examined women only; the majority of studies reported results for mixed populations (**Table 1**).

Healthy ageing, and any other term used as a synonym, was defined by including various domains of information in each study. These were grouped in the following categories: survival to a specific age or during follow-up, health status (either self-reported or measured by specific questionnaires), physical performance (including information regarding mobility, disabilities and/or difficulties in activities of daily living (ADL) and instrumental activities of daily living (iADL)), diseases (including chronic diseases and cancer), mental health and cognition status, subjective measurements of the participants (life satisfaction, happiness, and pain) and other (anthropometric measurements, personal assistance, social support) (Figure 2). Most of the studies (23 out of 28) included physical performance in their definition of healthy ageing and more than half of them included information regarding diseases and mental health (18 and 16 out of 28 respectively). Survival to a specific age was also an area often found in the definition of healthy ageing, whereas health status and subjective measurements were not so often included. (In the supplemental table: 'Table A1'the areas of information included in the definition of healthy ageing are provided per study).

In **Table 2** the analytical results of this systematic review are presented. Relevant statistics per study are provided for every group of smoking and alcohol consumption variable and for the most and least adjusted models; confounders used for the final adjustment are also provided.

Quality Assessment

Of the 28 studies, one was evaluated as having high risk of bias, nine as moderate and 18 as having low. In aggregate, the quality of the included studies was above moderate. As it was expected, attrition and confounding were those issues that primarily contributed to moderate and high bias. Specifically, 18 out of 28 studies reported moderate or high risk of bias regarding the fact that the population lost to follow-up may be associated with key characteristics that could influence the observed relationship between the outcome and the factors. Similar results were also observed for the confounders' domain, where 18 out of 23 studies were characterised as having moderate risk of bias, meaning that important confounders may have not been appropriately accounted in the final model. Supplemental table: 'Table A2'.

Meta-analysis

Smoking and Healthy Ageing

From the 27 studies providing results for smoking we did a meta-analysis with 18 studies. We excluded studies that provided results using: i) healthy/successful years;[30,42] ii) Risk Ratio;[40] iii) coefficients from linear mixed models or generalised estimation equation models;[41,50] and iv) cigarette-packs.[45,52,53] All studies included in the meta-analysis were of moderate and low risk of bias.

Never smokers compared to current smokers had more than double increased odds of ageing healthily (OR 2.36, 95%CI 2.03-2.75, I^2 43.30%, 7 studies) even after adjusting for

For peer review only - http://bmjopen.bmj.com/site/about/guidelines.xhtml

publication bias with the trim-and-fill algorithm (OR 2.11, 95%CI 1.82-2.45, 10 studies). Never smokers had increased odds ratios when compared to former smokers as well (OR 1.32, 95%CI 1.23-1.41, I^2 32.80%, 5 studies); no change of the combined effect estimate was observed by the trim-and-fill method. Never smokers were more likely to experience healthy ageing than current or former smokers (OR 1.29, 95%CI 1.16-1.43, I^2 0.00%, 5 studies) and this finding marginally altered when we adjusted for publication bias (OR 1.27, 95%CI 1.14-1.40, 9 studies). Never or former smokers were more likely to age in a healthy way compared to current smokers (OR 1.72, 95%CI 1.20-2.47, I^2 87.20%, 6 studies) however, when we adjusted for publication bias this finding was no longer significant (OR 1.20, 95%CI 0.83-1.73, 9 studies).

There were also two studies that reported hazard ratios (HR);[47,48] the pooled HR of never smokers compared to current smokers was 1.55 (95%CI 1.18-2.02); while the pooled HR of former smokers compared to never smokers was 1.30 (95%CI 1.07-1.59). Egger test was not significant in any analysis that we performed (p-value>0.05), hence no evidence for publication bias was provided.

Alcohol Consumption and Healthy Ageing

From the 21 studies that provided statistics for the association of alcohol consumption with healthy ageing we performed meta-analyses using nine studies. We excluded studies for the following reasons: i) the standardisation of alcohol consumption was not possible;[28,38,40,44,46,48,52-54] ii) they provided healthy years[30] or β coefficients.[41,50]

Drinkers compared to non-drinkers had increased odds of ageing healthily (OR 1.28, 95%CI 1.08-1.52, I^2 72.10%, 5 studies); the same beneficial association of alcohol was also observed when we pooled results among studies that reported findings of light consumption (<1 drinker per day) and moderate consumption (1-2 drinks per day) compared to non-drinkers: (OR 1.12, 95%CI 1.03-1.22, I^2 0.00%, 3 studies); (OR 1.35, 95%CI 0.93-1.97, I^2 71.40%, 4

For peer review only - http://bmjopen.bmj.com/site/about/guidelines.xhtml

studies). However, in the latter comparison (i.e. moderate consumption compared to non-drinkers) results were not statistically significant (p-value 0.112). We also pooled results of the studies reporting high (>2-4 drinks per day) consumption compared to non-drinkers; OR 1.25, (95%CI 1.09-1.44, I^2 0.00%, 3 studies). Results changed when we used the trim-and-fill algorithm only to the case of moderate to non-drinkers (OR 1.10, 95%CI 0.77-1.57, 6 studies). Egger test of bias was significant only in the analysis of light drinkers (p-value 0.043). Analytical results are presented in **Table 3**. The synthesis of the results is provided in **Figure 3** and **Figure 4**.

Discussion

Based on our knowledge, this systematic review is the first to examine the association of smoking and alcohol consumption with healthy ageing, which included a meta-analysis to produce a pooled effect estimate and also examined eligible studies for publication bias. 23 studies out of 27 reported a positive association between never or former smoking and healthy ageing and four reported a non-significant relationship. With regards to alcohol consumption, results were mixed but a positive association of drinking with healthy ageing was reported in 12 out of 22 studies. Nine studies did not find an association and one study reported a negative one.

The current systematic review adds to the plethora of evidence that smoking is associated with worse outcomes in older age. In fact, the only study that reported a positive association between current smoking and healthy ageing was that of Li et al. (2001)[40] where smokers exhibit increased risk ratios of healthy survival (RR 1.11, 95%CI 1.01-1.21); however authors recommend to not trusting this finding as the population attributable percentage (PARP) is very low after the five years of the follow-up (2.55%). In aggregate, being a non-smoker or a former smoker increases considerably the odds of ageing healthily. From our meta-analysis we found that never smokers have more than double the odds of experiencing healthy ageing

For peer review only - http://bmjopen.bmj.com/site/about/guidelines.xhtml

(OR 2.36, 95%CI 2.03-2.75) compared to current smokers. Furthermore, never smokers are also more likely to age in a healthy way by more than 30% (OR 1.32, 95%CI 1.23-1.41) compared to former smokers. These findings indicate that smoking cessation is always beneficial and come in accordance with a 50 years study on doctors' mortality in relation with smoking. In this study, it was revealed that smoking cessation at age 60, 50, 40, or 30 years could increase life expectancy by 3, 6, 9, or 10 years respectively.[55] Our findings also confirm previous research examining the beneficial effect of non-smoking on successful ageing.[56] In addition, we found that never/former smokers compared to current smokers have an increased likelihood of healthy ageing (OR 1.72, 95%CI 1.20-2.47), which becomes non-significant when we adjust for publication bias (OR 1.20, 95%CI 0.83-1.73). This comes in agreement with the finding of a study examining the transitions within smoking categories and successful ageing, where former compared to current smokers cannot predict successful ageing.[57]

Positive associations between drinking and healthy ageing refer to limited alcohol consumptions; for example 1-40 gr per day,[37] 1-14 drinks per week,[38] 1-14 units per week for women and 1-21 units per week for men[46]. The one study reporting a negative association is that of Willcox et al. (2006);[54] however, this finding should be interpreted with caution as drinkers with more than 3 drinks per day are compared to non-drinkers only and there is no comparison among light or moderate drinkers and non-drinkers. From our meta-analysis, even if it included limited number of studies, we concluded that compared to never drinkers reasonable alcohol consumption is beneficial to healthy ageing; pooled OR for drinkers 1.28 (95%CI 1.08-1.52), light drinkers 1.12 (95%CI 1.03-1.22), moderate drinkers 1.35 (95%CI 0.93-1.97) and high drinkers 1.25 (95%CI 1.09-1.44). Nevertheless, associations are marginally statistically significant, while for the moderate category the pooled effect estimate is non-significant, so extra caution is needed before making a final conclusion.

For peer review only - http://bmjopen.bmj.com/site/about/guidelines.xhtml

Several studies of the associations of alcohol consumption with healthy ageing were excluded from our meta-analyses. However, among those there were seven studies that reported positive association between alcohol consumption and healthy ageing,[38,40,41,44,46,48,50] four studies reported non-significant association,[28,30,52,53] and one study reported a negative association.[54] As a consequence, we expect that including these studies in our meta-analyses, the positive effect of light to moderate alcohol drinking would be reinforced. This finding comes in accordance with the fact that light to moderate alcohol consumption (≤1 drink per day) is also associated with a reduced risk of multiple cardiovascular outcomes,[11] better cognition and well-being,[58] and a reduced risk of substantial functional health decline.[59]

However, relating moderate alcohol consumption with health benefits should not come without question since there are many other issues to consider. Our results could be biased by confounders that have not been taken into account, such as health status or former and occasional drinkers to be counted as non-drinkers.[60] For instance, in the sub-group of drinkers the positive effect comes mainly from the study of LaCroix et al. (2016);[39] however, in that study former drinkers were also assumed as non-drinkers.

In addition, since typical drink sizes vary per country as well as the recommended daily or weekly amounts,[61] we cannot be sure that participants have been assigned to the correct drinking category. Our results should also be questioned regarding the fact that alcohol consumption was only assessed at baseline (exception to this is the study of Sun et al. (2011))[49]; during the baseline assessment some cohorts could have had middle-aged participants drinking up to four drinks per day and that could be considered a moderate consumption. However, older people do not have the same tolerance to alcohol[62] and for them moderate consumption should consist of up to two drinks per day. Likewise, it should have been considered that older adults tend to consume less alcohol than middle-aged groups[63] and hence baseline assessments perhaps are not representative of alcohol consumption in an older

For peer review only - http://bmjopen.bmj.com/site/about/guidelines.xhtml

age. Nevertheless, more research is required in order to establish or reject the beneficial effects of limited alcohol consumption. In an extended systematic review and meta-analysis of alcohol impact on mortality risk, the benefits of low-volume drinkers largely disappeared once studies were controlled for several design and methodological issues.[64] Mendelian randomisation studies, helping to examine causal relationships in observational studies by limiting confounding and reverse causation risk of bias,[65] may also contribute to elucidating if this beneficial effect of light alcohol consumption with healthy ageing is a true biological effect or not. For instance, a recent study of this type found no causal association between alcohol consumption and Alzheimer's disease.[66]

Strengths and Limitations

The fact that this review was independently double screened, taking into account previous systematic reviews in the field and the reference lists of included papers, allows a good amount of confidence that all relevant studies were included. Regarding the quality assessment of the studies, limited disagreement among the six different domains per study was reached between the two reviewers, who independently assessed them, concluding that the quality assessment tool was reliable and did not allow great amount of misjudgement. Attrition rate and not allowing important confounders to be included in the final models were important factors contributing to the quality of the considered studies. Hence future research should consider these domains more thoroughly.

However, the following limitations have to be taken into account. Smoking and alcohol consumption were not measured or/and defined in a consistent way among the different cohorts; hence it was not possible to create a pooled effect size estimate across all eligible studies. In addition, healthy ageing definition was a source of heterogeneity since each study provided its own definition. However, it has to be highlighted that majority of studies had definitions that included areas of physical performance, in agreement with the Depp and

For peer review only - http://bmjopen.bmj.com/site/about/guidelines.xhtml

Jeste, (2006) review on successful ageing.[56] In addition, most of the studies used a categorical definition of smoking making impossible the assessment of abstinence time or pack-years on healthy ageing. Alcohol measurement and drinking categorisation were also another part of the underlying heterogeneity. Similar comments have also been reported in other systematic reviews examining alcohol consumption.[64,67] Furthermore, self-reported questionnaires and the fact that each study was adjusted by using a different set of covariates, different follow-up time and attrition rate, and the different definition of healthy ageing increases the heterogeneity and the likelihood of bias. Especially for alcohol, research has shown that self-reported consumption is mostly underestimated.[68]

Due to the limited number of studies included in our meta-analyses we did not assess publication bias by funnel plots; instead we used Egger test and even though it was statistically non-significant in the majority of cases we applied the trim-and-fill method to examine the robustness of our findings. We used random effect meta-analysis to consider the heterogeneity of the studies, even though I^2 was not considerable in all analyses (**Table 3**). We did not assess heterogeneity with the Cochran's Q statistic due to the quite limited number of studies; performing meta-regression analysis and assessing the confounding effect of covariates was not possible either. However, we did evaluate the robustness of our findings by also implementing the Paule and Mandel (PM) estimator to assess between study variability and estimate our pooled results, as simulations have shown that the PM estimator is less biased and more efficient than other alternatives.[69] We did not observe any considerable fluctuations in our results (Supplemental table: 'Table A3').

From our review, it is evident that the majority of the studies have been implemented in high-income countries (24 out of 28 studies). Thus, in accordance with a previous study,[70] our findings have highlighted the limited research on ageing in low and middle income countries (LMIC), even though by 2050 80% of the people aged 60 years and over will live there.[71] In

For peer review only - http://bmjopen.bmj.com/site/about/guidelines.xhtml

addition, the economic burden of smoking in those countries is the heaviest as it is estimated that by 2030 four out of five smoking-related deaths will occur there.[72] Nowadays, 80% of the more than one billion smokers live in LMIC.[73]

Conclusions

In conclusion, smoking abstinence and smoking cessation are positively associated with healthy ageing. As it takes more than 20 years for most smoking-related diseases to develop, the best practice would be to enforce smoking prevention policies, such as marketing bans and high taxation, and reduce smoking uptake among younger cohorts.[1] A positive relationship between limited consumptions of alcohol and healthy ageing could also be argued but more research should be done to reinforce this result. From our research it becomes evident that there is a need to make studies comparable in order to conclude with more confidence the generalisability of our findings. This could be achieved by adopting similar measurements of smoking and alcohol behaviour and by implementing a unanimous definition and metric of healthy ageing. Finally, further research should focus on the examination of ageing in the older population of low-and-middle income countries.

Supplementary Material

Appendix1.docx (MOOSE checklist)

Appendix2.pdf (Prospero Protocol)

Appendix3.docx (Searching Methodology)

Table A1.docx (Healthy Ageing Definition)

Table A2.docx (Quality Assessment)

Table A3. docx (Sensitivity analysis of meta-analytic results)

Contributors

For peer review only - http://bmjopen.bmj.com/site/about/guidelines.xhtml

For peer review only

CD designed the study, carried out the literature review, data extraction, statistical analysis, data interpretation, article preparation, article review and correspondence. BS contributed to the data interpretation and article review. CK contributed to the literature review and data extraction. AMP contributed to the study design, data interpretation, article preparation and article review. All authors contributed to the final report and approved the final version.

Acknowledgements

We thank Chih-Cheng Chang for his contribution to the translation of the non-English study.

We thank ATHLOS (Ageing Trajectories of Health: Longitudinal Opportunities and Synergies) project, funded by the European Union's Horizon 2020 Research and Innovation Programme under grant agreement number 635316.

Funding

This project falls under the ATHLOS (Ageing Trajectories of Health: Longitudinal Opportunities and Synergies) project, funded by the European Union's Horizon 2020 Research and Innovation Programme under grant agreement number 635316. The sponsor had no participation in the study design, data extraction, data interpretation, or writing of this study.

A. M. P. was supported by the MRC MR/K021907/1.

A. K. was supported by a grant funded by the National Institute for Health Research (NIHR) Biomedical Research Centre at South London and Maudsley National Health Service (NHS) Foundation Trust and Kings' College London where most of this work was conducted. The views expressed are those of the authors and not necessarily those of NIHR, the NHS, or the UK Government Department of Health. A .K. is now formally based at the Liverpool School of Tropical Medicine.

Declaration of interests

For peer review only - http://bmjopen.bmj.com/site/about/guidelines.xhtml

All authors declare no conflicts of interest.

Data sharing statement

Statistical code is available upon request to the corresponding author; no other additional data are available.

References

1. Gibson G, Loddenkemper R, Sibille Y, et al. The European Lung White Book. 1st ed: European Respiratory Society 2013.
2. Peters R, Poulter R, Warner J, et al. Smoking, dementia and cognitive decline in the elderly, a systematic review. *BMC Geriatr* 2008;8:36. doi: 10.1186/1471-2318-8-36
3. Gellert C, Schottker B, Brenner H. Smoking and all-cause mortality in older people: systematic review and meta-analysis. *Arch Intern Med* 2012;172(11):837-44. doi: 10.1001/archinternmed.2012.1397
4. GHK. *A study on liability & the health costs of smoking DG SANCO (2008/C6/046) updated final report* http://ec.europa.eu/health//sites/health/files/tobacco/docs/tobacco_liability_final_en.pdf (accessed December 13, 2016).
5. Goodchild M, Nargis N, Tursan d'Espaignet E. Global economic cost of smoking-attributable diseases. *Tobacco Control* 2017 doi: 10.1136/tobaccocontrol-2016-053305
6. World Health Organization. *Global status report on alcohol and health 2014.* 2014. http://apps.who.int/iris/bitstream/10665/112736/1/9789240692763_eng.pdf.
7. Organization WH. Alcohol, Fact Sheet: World Health Organization.
8. Chapter 2 - Alcohol. Center for Substance Abuse Treatment Substance Abuse Among Older Adults Treatment Improvement Protocol (TIP) Series, No 26. Rockville (MD): Substance Abuse and Mental Health Services Administration (US) 1998. Available from: https://www.ncbi.nlm.nih.gov/books/NBK64419/.
9. The Royal College of Psychiatrists. *Alcohol and Older People* http://www.rcpsych.ac.uk/healthadvice/problemsdisorders/alcoholandolderpeople.aspx (accessed February 7, 2017).
10. Mukamal KJ, Kuller LH, Fitzpatrick AL, et al. Prospective study of alcohol consumption and risk of dementia in older adults. *Jama* 2003;289(11):1405-13.
11. Ronksley PE, Brien SE, Turner BJ, et al. Association of alcohol consumption with selected cardiovascular disease outcomes: a systematic review and meta-analysis. *BMJ* 2011;342 doi: 10.1136/bmj.d671
12. De Leon J, Rendon DM, Baca-Garcia E, et al. Association between smoking and alcohol use in the general population: stable and unstable odds ratios across two years in two different countries. *Alcohol Alcohol* 2007;42(3):252-7. doi: 10.1093/alcalc/agm029
13. Stotts AL, Schmitz JM, Grabowski J. Concurrent treatment for alcohol and tobacco dependence: are patients ready to quit both? *Drug Alcohol Depend* 2003;69(1):1-7.
14. Xu WH, Zhang XL, Gao YT, et al. Joint effect of cigarette smoking and alcohol consumption on mortality. *Prev Med* 2007;45(4):313-9. doi: 10.1016/j.ypmed.2007.05.015
15. Hart CL, Davey Smith G, Gruer L, et al. The combined effect of smoking tobacco and drinking alcohol on cause-specific mortality: a 30 year cohort study. *BMC Public Health* 2010;10:789. doi: 10.1186/1471-2458-10-789

For peer review only

For peer review only - http://bmjopen.bmj.com/site/about/guidelines.xhtml

16. United Nations. *World Population Ageing 2015.* 2015. http://www.un.org/en/development/desa/population/publications/pdf/ageing/WPA2015_Report.pdf.
17. Shamseer L, Moher D, Clarke M, et al. Preferred reporting items for systematic review and meta-analysis protocols (PRISMA-P) 2015: elaboration and explanation. *BMJ* 2015;349:g7647. doi: 10.1136/bmj.g7647
18. Stroup DF, Berlin JA, Morton SC, et al. Meta-analysis of observational studies in epidemiology: A proposal for reporting. *JAMA* 2000;283(15):2008-12. doi: 10.1001/jama.283.15.2008
19. Daskalopoulou C, Stubbs B, Kralj C, et al. Physical activity and healthy ageing: A systematic review and meta-analysis of longitudinal cohort studies. *Ageing Res Rev* 2017;38:6-17. doi: 10.1016/j.arr.2017.06.003
20. Hayden JA, van der Windt DA, Cartwright JL, et al. Assessing bias in studies of prognostic factors. *Ann Intern Med* 2013;158(4):280-6. doi: 10.7326/0003-4819-158-4-201302190-00009
21. Larsson SC, Wallin A, Wolk A, et al. Differing association of alcohol consumption with different stroke types: a systematic review and meta-analysis. *BMC Med* 2016;14(1):178. doi: 10.1186/s12916-016-0721-4
22. DerSimonian R, Laird N. Meta-analysis in clinical trials. *Control Clin Trials* 1986;7(3):177-88.
23. Higgins JP, Thompson SG, Deeks JJ, et al. Measuring inconsistency in meta-analyses. *Bmj* 2003;327(7414):557-60. doi: 10.1136/bmj.327.7414.557
24. Sterne JA, Egger M, Smith GD. Systematic reviews in health care: Investigating and dealing with publication and other biases in meta-analysis. *BMJ* 2001;323(7304):101-5.
25. Egger M, Smith GD, Schneider M, et al. Bias in meta-analysis detected by a simple, graphical test. *BMJ* 1997;315(7109):629-34. doi: 10.1136/bmj.315.7109.629
26. Duval S, Tweedie R. Trim and fill: A simple funnel-plot-based method of testing and adjusting for publication bias in meta-analysis. *Biometrics* 2000;56(2):455-63.
27. Andrews G, Clark M, Luszcz M. Successful Aging in the Australian Longitudinal Study of Aging: Applying the MacArthur Model Cross–Nationally. *Journal of Social Issues* 2002;58(4):749-65. doi: 10.1111/1540-4560.00288
28. Bell CL, Chen R, Masaki K, et al. Late-life factors associated with healthy aging in older men. *J Am Geriatr Soc* 2014;62(5):880-8. doi: 10.1111/jgs.12796
29. Britton A, Shipley M, Singh-Manoux A, et al. Successful aging: the contribution of early-life and midlife risk factors. *J Am Geriatr Soc* 2008;56(6):1098-105. doi: 10.1111/j.1532-5415.2008.01740.x
30. Burke GL, Arnold AM, Bild DE, et al. Factors associated with healthy aging: the cardiovascular health study. *J Am Geriatr Soc* 2001;49(3):254-62.
31. Ford AB, Haug MR, Stange KC, et al. Sustained personal autonomy: a measure of successful aging. *J Aging Health* 2000;12(4):470-89. doi: 10.1177/089826430001200402
32. Gu D, Zhang Z, Zeng Y. Access to healthcare services makes a difference in healthy longevity among older Chinese adults. *Soc Sci Med* 2009;68(2):210-9. doi: 10.1016/j.socscimed.2008.10.025
33. Guralnik JM, Kaplan GA. Predictors of healthy aging: prospective evidence from the Alameda County study. *Am J Public Health* 1989;79(6):703-8.
34. Gureje O, Oladeji BD, Abiona T, et al. Profile and determinants of successful aging in the Ibadan Study of Ageing. *J Am Geriatr Soc* 2014;62(5):836-42. doi: 10.1111/jgs.12802
35. Hamer M, Lavoie KL, Bacon SL. Taking up physical activity in later life and healthy ageing: the English longitudinal study of ageing. *Br J Sports Med* 2014;48(3):239-43. doi: 10.1136/bjsports-2013-092993
36. Hodge AM, English DR, Giles GG, et al. Social connectedness and predictors of successful ageing. *Maturitas* 2013;75(4):361-6. doi: 10.1016/j.maturitas.2013.05.002
37. Hodge AM, O'Dea K, English DR, et al. Dietary patterns as predictors of successful ageing. *J Nutr Health Aging* 2014;18(3):221-7. doi: 10.1007/s12603-013-0405-0

For peer review only - http://bmjopen.bmj.com/site/about/guidelines.xhtml

38. Kaplan MS, Huguet N, Orpana H, et al. Prevalence and Factors Associated With Thriving in Older Adulthood: A 10-Year Population-Based Study. *J Gerontol A Biol Sci Med Sci* 2008;63(10):1097-104.
39. LaCroix AZ, Rillamas-Sun E, Woods NF, et al. Aging Well Among Women Veterans Compared With Non-Veterans in the Women's Health Initiative. *Gerontologist* 2016;56 Suppl 1:S14-26. doi: 10.1093/geront/gnv124
40. Li C, Zhang M, He Y, et al. Impact of health behavior on successful aging: A 5-year follow-up study among community elderly. *Chinese Mental Health Journal* 2001(15):324-26 (in Chinese).
41. Liu LF, Su PF. What factors influence healthy aging? A person-centered approach among older adults in Taiwan. *Geriatr Gerontol Int* 2017;17(5):697-707. doi: 10.1111/ggi.12774
42. Newman AB, Arnold AM, Naydeck BL, et al. "Successful aging": effect of subclinical cardiovascular disease. *Arch Intern Med* 2003;163(19):2315-22. doi: 10.1001/archinte.163.19.2315
43. Newson RS, Witteman JC, Franco OH, et al. Predicting survival and morbidity-free survival to very old age. *Age (Dordr)* 2010;32(4):521-34. doi: 10.1007/s11357-010-9154-8
44. Pruchno RA, Wilson-Genderson M. A Longitudinal Examination of the Effects of Early Influences and Midlife Characteristics on Successful Aging. *J Gerontol B Psychol Sci Soc Sci* 2015;70(6):850-9. doi: 10.1093/geronb/gbu046
45. Reed DM, Foley DJ, White LR, et al. Predictors of healthy aging in men with high life expectancies. *Am J Public Health* 1998;88(10):1463-8.
46. Sabia S, Singh-Manoux A, Hagger-Johnson G, et al. Influence of individual and combined healthy behaviours on successful aging. *CMAJ* 2012;184(18):1985-92. doi: 10.1503/cmaj.121080
47. Sarnak MJ, Katz R, Fried LF, et al. Cystatin C and aging success. *Arch Intern Med* 2008;168(2):147-53. doi: 10.1001/archinternmed.2007.40
48. Shields M, Martel L. Healthy living among seniors. *Health Rep* 2006;16 Suppl:7-20.
49. Sun Q, Townsend MK, Okereke OI, et al. Alcohol Consumption at Midlife and Successful Ageing in Women: A Prospective Cohort Analysis in the Nurses' Health Study. *PLOS Medicine* 2011;8(9):e1001090. doi: 10.1371/journal.pmed.1001090
50. Tampubolon G. Trajectories of the healthy ageing phenotype among middle-aged and older Britons, 2004–2013. *Maturitas* 2016;88:9-15. doi: 10.1016/j.maturitas.2016.03.002
51. Terry DF, Pencina MJ, Vasan RS, et al. Cardiovascular risk factors predictive for survival and morbidity-free survival in the oldest-old Framingham Heart Study participants. *J Am Geriatr Soc* 2005;53(11):1944-50. doi: 10.1111/j.1532-5415.2005.00465.x
52. Vaillant GE, Mukamal K. Successful aging. *Am J Psychiatry* 2001;158(6):839-47. doi: 10.1176/appi.ajp.158.6.839
53. Vaillant GE, Western RJ. Healthy aging among inner-city men. *Int Psychogeriatr* 2001;13(4):425-37.
54. Willcox BJ, He Q, Chen R, et al. Midlife risk factors and healthy survival in men. *Jama* 2006;296(19):2343-50. doi: 10.1001/jama.296.19.2343
55. Doll R, Peto R, Boreham J, et al. Mortality in relation to smoking: 50 years' observations on male British doctors. *BMJ* 2004;328(7455):1519. doi: 10.1136/bmj.38142.554479.AE
56. Depp CA, Jeste DV. Definitions and predictors of successful aging: a comprehensive review of larger quantitative studies. *Am J Geriatr Psychiatry* 2006;14(1):6-20. doi: 10.1097/01.JGP.0000192501.03069.bc
57. Pruchno R, Hahn S, Wilson-Genderson M. Cigarette smokers, never-smokers, and transitions: implications for successful aging. *Int J Aging Hum Dev* 2012;74(3):193-209. doi: 10.2190/AG.74.3.b
58. Lang I, Wallace RB, Huppert FA, et al. Moderate alcohol consumption in older adults is associated with better cognition and well-being than abstinence. *Age Ageing* 2007;36(3):256-61. doi: 10.1093/ageing/afm001

For peer review only - http://bmjopen.bmj.com/site/about/guidelines.xhtml

59. Chen LY, Hardy CL. Alcohol consumption and health status in older adults: a longitudinal analysis. *J Aging Health* 2009;21(6):824-47. doi: 10.1177/0898264309340688
60. Stockwell T, Greer A, Fillmore K, et al. How good is the science? *Bmj* 2012;344:e2276; author reply e94. doi: 10.1136/bmj.e2276.
61. International Alliance for Responsible Drinking. *Drinking Guidelines - Data by Country - Worldwide* 2016. http://www.iard.org/policy-tables/drinking-guidelines-general-population.
62. Institute of Alcohol Studies. *Older people and alcohol Factsheet* http://www.ias.org.uk/uploads/pdf/Factsheets/Alcohol%20and%20older%20people%20FS%20May%202013.pdf (accessed December 14, 2016).
63. NHS Health. *Alcohol and Ageing. Is alcohol a major threat to healthy ageing for the baby boomers?* http://lx.iriss.org.uk/sites/default/files/resources/Alcohol%20and%20ageing.pdf (accessed December 15, 2016).
64. Stockwell T, Zhao J, Panwar S, et al. Do "Moderate" Drinkers Have Reduced Mortality Risk? A Systematic Review and Meta-Analysis of Alcohol Consumption and All-Cause Mortality. *J Stud Alcohol Drugs* 2016;77(2):185-98. doi: 10.15288/jsad.2016.77.185
65. Smith GD, Ebrahim S. Mendelian randomization: prospects, potentials, and limitations. *International Journal of Epidemiology* 2004;33(1):30-42. doi: 10.1093/ije/dyh132
66. Andrews SJ, Anstey KJ. Association between alcohol consumption and Alzheimer's disease: A Mendelian Randomization Study. *bioRxiv* 2017 doi: 10.1101/190165
67. Reid MC, Boutros NN, O'Connor PG, et al. The health-related effects of alcohol use in older persons: a systematic review. *Subst Abus* 2002;23(3):149-64. doi: 10.1080/08897070209511485
68. Stockwell T, Zhao J, Macdonald S. Who under-reports their alcohol consumption in telephone surveys and by how much? An application of the 'yesterday method' in a national Canadian substance use survey. *Addiction* 2014;109(10):1657-66. doi: 10.1111/add.12609
69. Veroniki AA, Jackson D, Viechtbauer W, et al. Methods to estimate the between-study variance and its uncertainty in meta-analysis. *Research synthesis methods* 2016;7(1):55-79. doi: 10.1002/jrsm.1164
70. Chatterji S, Byles J, Cutler D, et al. Health, functioning, and disability in older adults—present status and future implications. *The Lancet* 2015;385(9967):563-75. doi: 10.1016/s0140-6736(14)61462-8
71. WHO.World Health Organization. *Ageing and health Factsheet* http://www.who.int/mediacentre/factsheets/fs404/en/ (accessed November 8, 2016).
72. World Health Organization. *WHO report on the global tobacco epidemic, 2008 : the MPOWER package.* 2008. http://apps.who.int/iris/bitstream/10665/43818/1/9789241596282_eng.pdf.
73. World Health Organization. *Tobacco Factsheet* http://www.who.int/mediacentre/factsheets/fs339/en (accessed December 9, 2016).

For peer review only - http://bmjopen.bmj.com/site/about/guidelines.xhtml

Table 1: Baseline characteristics of the eligible studies

Studies/Authors	Country	Panel	Data collection period	Follow-up*	Sample Size	Gender	Baseline Age
Andrews et al., 2002[27]	Australia	Australian Longitudinal Study of Aging (ALSA)	1992	8	1,403	55% men	> 70
Bell et al., 2014[28]	USA	Honolulu Heart Program (HHP)	1991 - 1993	up to 21 years	1,292	100% men	71-82
Britton et al., 2008[29]	England	Whitehall II study	1985 - 1988	17	5,823	71% men	35 - 55
Burke et al., 2001[30]	USA	Cardiovascular Health Study (CHS)	1989 - 1990, 1992 - 1993	6.5 and 3.5	3,342	39% men	> 65
Ford et al., 2000[31]	USA	N/A	1993	2	602	33% men	> 70
Gu et al., 2009[32]	China	Chinese Longitudinal Healthy Longevity Survey (CLHLS)	2002	3	15,972	45% men	65 - 109
Guralnik & Kaplan, 1989[33]	USA	Alameda County Study	1965	19	841	N/A	46 - 70
Gureje et al., 2014[34]	Nigeria	Ibadan Study of Ageing (ISA)	Aug 2003 - Nov 2004	5.3	930	61% men	> 65
Hamer et al., 2013[35]	England	English Longitudinal Study of Ageing (ELSA)	2002 - 2003	8	3,454	42% men	63.7 (M)
Hodge, English et al., 2013[36]	Australia	Melbourne Collaborative Cohort Study	1990 - 1994	11.7	5,512	37% men	63 (M)
Hodge, O'Dea et al., 2014[37]	Australia	Melbourne Collaborative Cohort Study	1990 - 1994	11.1 (WM)	6,309	39% men	64.1 (WM)
Kaplan et al., 2008[38]	Canada	Canadian National Population Health Survey (NPHS)	1994 - 1995	10	2,432	44% men	65 - 85
LaCroix et al., 2016[39]	USA	Women's Health Initiative (WHI)	1993 - 1998	16	68,153	100% women	68.9 (WM)
Li et al., 2001[40]	China	Shanghai Mental Health Center	1987	5	3,024	43% men	67.3 (M)
Liu & Su, 2016[41]	Taiwan	Taiwan Longitudinal Study on Aging (TLSA)	1993	14	3,155	56% men	71.7 (M)

For peer review only - http://bmjopen.bmj.com/site/about/guidelines.xhtml

For peer review only

Newman et al., 2003[42]	USA	Cardiovascular Health Study (CHS)	1989 - 1990, 1992 - 1993	8	2,932	39% men	71.9 (WM)
Newson et al., 2010[43]	Netherlands	Rotterdam Study	1990 - 1993	7.9	2,008	34% men	75.8 (M)
Pruchno & Wilson-Genderson, 2015[44]	USA	ORANJ BOWL: Ongoing Research on Aging in New Jersey: Bettering Opportunities for Wellness in Life	2006 -2008	4	2,614	37% men	60.5 (WM)
Reed et al., 1998[45]	USA	Honolulu Heart Program (HHP)	1965 - 1968	28	8,006	100% men	45 - 68
Sabia et al., 2012[46]	England	Whitehall II study	1991 - 1994	>16.3(Med)	5,100	70% men	51.3 (M)
Sarnak et al., 2008[47]	USA	Cardiovascular Health Study (CHS)	1989-1990 and 1992-1993 (African-American cohort)	4.3	2,140	38% men	74
Shields & Martel, 2006[48]	Canada	National Population Health Survey (NPHS)	1994-1995	8	1,309	N/A	> 65
Sun et al., 2011[49]	USA	Nurses' Health Study (NHS)	1980-1986	20 and 16	13,894	100% women	58 (Med)
Tampubolon, 2016[50]	England	English Longitudinal Study of Ageing (ELSA)	2004	9	14,765	46% men	50 - 89
Terry et al., 2005[51]	USA	Framingham Heart Study (FHS)	1948-1971	45	2,531	44% men	40 - 50
Vaillant & Mukamal, 2001[52]	USA	Study of Adult Development at Harvard University	circa 1940	until 60 or death	724	100% men	born mainly in the 1920s
Vaillant & Western, 2001[53]	USA	Study of Adult Development	1940 - 1945	60 y. (until 70 years or death)	456	100% men	14
Willcox et al., 2006[54]	USA	Honolulu Heart Program/Honolulu Asia Aging Study (HHP/HAAS)	1965-1968	40	5,820	100% men	54 (WM)

*: mean years, unless otherwise specified; M:mean; WM: Weighted Mean; Med: Median

For peer review only - http://bmjopen.bmj.com/site/about/guidelines.xhtml

Table 2: Aggregated Results of Smoking and Alcohol Consumption to Healthy Ageing

Authors	Smoking Variable	Statistics and 95%CI	Alcohol Variable	Statistics and 95%CI	Confounders
Andrews et al., 2002[27]	Past smoker (Yes or No)	no statistically significant (N/A)	-	-	Sociodemographic, Economic
Bell et al., 2014[28]	Past, Current, Never	Unhealthy vs Healthy Survival; OR, 95%CI<br>Past: 1.03, (0.78-1.35)<br>Current: 1.99, (1.06-3.75)<br>Never: Ref	Never (0 ounces/month);<br>Moderate to Heavy (>15 ounces/month, >1 drink per day);<br>Mild (>0–15 ounces/month, ≤1 drink per day)	Unhealthy vs Healthy Survival; OR, 95%CI<br>Never: 1.10, (0.81-1.50)<br>Moderate to Heavy: 1.04, (0.73-1.47)<br>Mild: Ref	Sociodemographic
Britton et al., 2008[29]	Never, Ex-smoker, Current	OR, 95%CI, Men:<br>Never: 2.7, (1.8-4.1), Not SEP adj: 3.2, (2.1-4.7)<br>Ex-smoker: 2.5, (1.6-3.7), Not SEP adj: 2.6, (1.8-4.0)<br>Current smoker: Ref<br>Women:<br>Never: 2.2, (1.3-3.7), Not SEP adj: 2.4, (1.5-3.9)<br>Ex-smoker: 2.2, (1.3-3.7), Not SEP adj: 2.6, (1.5-4.5)<br>Current smoker: Ref	Alcohol consumption in the previous week (1 Unit = 8 g ethanol).<br>0 unit/week, 1-14 units/week, 15 units/week	OR, 95%CI, Men:<br>0: 1.2, (0.9-1.8), Not SEP adj: 1.0, (0.7-1.4)<br>1-14: 1.0, (0.8-1.2), Not SEP adj: 1.0, (0.8-1.2)<br>15: Ref<br>Women<br>0: 0.5, (0.3-0.9), Not SEP adj: 0.3, (0.2-0.5)<br>1-14: 1.0, (0.7-1.5), Not SEP adj: 0.8, (0.5-1.1)<br>15: Ref	Sociodemographic, Economic, Model Characteristics

For peer review only - http://bmjopen.bmj.com/site/about/guidelines.xhtml

Burke et al., 2001[30]	Former, Current, Never	Proportion of HY, 95%CI:<br>Model with Behavioural Factors only<br>Former: 0.75, N/A<br>Current: 0.56, N/A<br>Never: Ref<br>Model with Behavioural Factors & Subclinical Disease Factors<br>Former: 0.78, (0.69, 0.88)<br>Current: 0.66, (0.56, 0.78)<br>Never: Ref	Wine Drink: Yes, No	Proportion of HY, 95%CI:<br>Model with Behavioural Factors only<br>Wine Drink: 1.11, (0.99, 1.24)<br>Model with Behavioural Factors & Subclinical Disease Factors<br>Wine Drink: 1.04, (0.93, 1.17)	Sociodemographic, Economic, Health Behaviour, Diseases and Physical Measurements
Ford et al., 2000[31]	Current Smoker: Yes, No	OR, 95%CI:<br>No: 2.14, (1.02-4.48)<br>Yes: Ref	Current drinker (wine, beer, or liquor): Yes, No	OR, 95%CI:<br>No: 1.00, (0.59-1.71)	Sociodemographic, Economic, Health Behaviour, Diseases and Physical Measurements, Attitude and Social Environment
Gu et al., 2009[32]	Current Smoker: Yes, No	OR, 95% CI of access to healthcare at present & in childhood on healthy survival<br>Model I: 1.05, (0.91-1.21)<br>Model II: 1.00, (0.86-1.16)<br>Model III: 1.00, (0.86-1.16)<br>No: Ref	Current Drinker: Yes, No	OR, 95% CI of access to healthcare at present & in childhood on healthy survival<br>Model I: 1.21, (1.05-1.39)<br>Model II: 1.11, (0.96-1.28)<br>Model III: 1.10, (0.96-1.27)<br>No: Ref	Model I: Sociodemographic, Economic<br>Model II: Sociodemographic, Economic, Attitude and Social Environment<br>Model III: Sociodemographic, Economic, Attitude and Social Environment, Model Characteristics

For peer review only - http://bmjopen.bmj.com/site/about/guidelines.xhtml

Guralnik & Kaplan, 1989[33]	Past-Never, Current	OR, 95%CI<br>High function vs all others (Dead or Lower/Moderate)<br>Health Practices Model: Past-Never: 2.8, (1.6-4.8), Current: Ref<br>Combined Model: Past-Never: 3.0, (1.8-5.1), Current: Ref<br><br>High function vs Deceased<br>Past-Never: 6.1, (3.3-8.3), Current: Ref<br>High function vs Low/Moderate<br>Past-Never: 2.2, (1.3, 3.8), Current: Ref	None, 1-60, >60 (drinks/month)	OR, 95%CI<br>High function vs all others (Dead or Lower/Moderate)<br>Health Practices Model: 1-60 vs None: 2.4, (1.2-4.8)<br>1-60 vs >60: 1.7, (0.6-5.0)<br>Combined Model: 1-60 vs None: 2.1, (1.1-4.1), 1-60 vs >60: 1.7, (0.6-5.1)<br><br>High function vs Deceased<br>1-60 vs None: 3.1, (1.5-6.5),<br>1-60 vs >60: 2.5, (0.7-8.2)<br>High function vs Low/Moderate<br>1-60 vs None: 2.2, (1.1-4.4)<br>1-60 vs >60: 1.3, (0.4-4.0)	Health Practices Model: Health Behaviour, Physical Measurement<br>Combined Model: Sociodemographic, Economic, Diseases, Physical Measurement, Health Behaviour
Gureje et al., 2014[34]	Ever smoking: Yes, No	OR, 95%CI<br>Total: No: 1.6, (0.71-3.46), Yes: Ref<br>Male: No: 4.7, (1.61-14.52), Yes: Ref<br>Female: No: 0.4, (0.22, 1.13), Yes: Ref	Ever having alcohol: Yes, No	OR, 95%CI<br>Total: No: 1.0, (0.51, 2.10), Yes: Ref<br>Male: No: 0.5, (0.10, 2.73), Yes: Ref<br>Female: No: 0.3, (0.47, 2.18), Yes: Ref	Sociodemographic, Economic, Health Behaviour, Diseases and Physical Measurements, Attitude and Social Environment
Hamer et al., 2013[35]	Current, Ex/Non-smoker	OR, 95%CI<br>Current: 0.66, (0.48-0.89)<br>Ex/Non-smokers: Ref	Daily, at least Weekly, Rarely, Never	-	Sociodemographic, Economic, Health Behaviour

For peer review only - http://bmjopen.bmj.com/site/about/guidelines.xhtml

Hodge, English et al., 2013[36]	Former, Current, Never	OR, 95% CI<br>Former: 0.72, (0.61, 0.84)<br>Current: 0.45, (0.32, 0.65)<br>Never: Ref	Alcohol intake (g/day)	OR, 95%CI<br>1–20: 1.06, (0.90-1.24)<br>21–40: 1.16, (0.93-1.46)<br>41–60: 1.02, (0.72-1.45)<br>60+: 1.25, (0.79-1.97)<br>None: Ref	Sociodemographic, Economic, Health Behaviour, Diseases and Physical Measurements, Attitude and Social Environment
Hodge, O'Dea et al., 2014[37]	Former, Current, Never	OR, 95%CI<br>Model without BMI and WHR<br>Former: 0.67, (0.59-0.77)<br>Current: 0.39, (0.30-0.50)<br>Never: Ref<br><br>Model with BMI and WHR<br>Former: 0.69, (0.60-0.79)<br>Current: 0.38, (0.29-0.48)<br>Never: Ref	Alcohol intake (g/day)	OR, 95%CI<br>Model without BMI and WHR<br>1-20: 1.19, (1.03-1.36)<br>21-40: 1.37, (1.11-1.67)<br>41-60: 1.27, (0.94-1.74)<br>60+: 1.17, (0.78-1.76)<br>None: Ref<br><br>Model with BMI and WHR<br>1-20: 1.17, (1.02-1.34)<br>21-40: 1.33, (1.09-1.63)<br>41-60: 1.29, (0.95-1.76)<br>60: 1.20, (0.80-1.80)<br>None: Ref	Sociodemographic, Economic, Health Behaviour, Diseases and Physical Measurements, Model Characteristics
Kaplan et al., 2008[38]	Smoker, Never smoker	OR, 95%CI<br>Thrivers vs NonThrivers: Smoker: 1.89, (1.10-3.23), Never smoker: Ref<br>Thrivers vs Deceased: Smoker: 4.35, (2.44-7.69), Never smoker: Ref	Moderate (1-14 drinks/week), None or Heavy	OR, 95%CI<br>Thrivers vs NonThrivers, Moderate: 1.78, (1.07-2.95), None-Heavy: Ref<br>Thrivers vs Deceased, Moderate: 2.22, (1.25-3.95), None-Heavy: Ref	Sociodemographic, Economic, Attitude and Social Environment, Health Behaviour, Diseases and Physical Measurements

For peer review only - http://bmjopen.bmj.com/site/about/guidelines.xhtml

LaCroix et al., 2016[39]	Current, Past, Never	OR, 95%<br>Crude Model, Veterans<br>Current: 0.35, (0.22-0.57), Past: 0.85, (0.71-1.02), Never: Ref<br>Adjusted Model, Veterans<br>Current: 0.31, (0.19-0.52), Past: 0.80, (0.66-0.97), Never: Ref<br>Crude Model, Non-Veterans<br>Current: 0.49, (0.45-0.54), Past: 0.83, (0.80-0.86), Never: Ref<br>Adjusted Model, Non-Veterans<br>Current: 0.50, (0.46-0.55), Past: 0.77, (0.74-0.80), Never: Ref<br><br>Model for Veterans Only:<br>Crude: Current: 0.35, (0.22-0.57), Past: 0.85, (0.71-1.02), Never: Ref<br>Adjusted: Current: 0.31, (0.18-0.52), Past: 0.79, (0.65-0.95), Never: Ref	Drinker, Non-Drinker	OR, 95%<br>Crude Model, Veterans<br>Non-drinker: 0.59, (0.48-0.73), Drinker: Ref<br>Adj. Model, Veterans<br>Non-drinker: 0.64, (0.51-0.81), Drinker: Ref<br>Crude Model, Non-Veterans<br>Non-drinker: 0.67, (0.64-0.69), Drinker: Ref<br>Adj. Model, Non-Veterans<br>Non-drinker: 0.71, (0.68-0.74), Drinker: Ref<br><br>Model for Veterans Only:<br>Crude: Non-drinker: 0.59, (0.48-0.73), Drinker: Ref<br>Adj.: Non-drinker: 0.62, (0.49-0.78), Drinker: Ref	Crude: Age<br>Adj.: Sociodemographic, Economic, Model Characteristics, Health Behaviour, Diseases and Physical Measurements
Li et al., 2001[40]	Current, Former, Never	RR, 95%CI<br>Current: 1.11, (1.01-1.21)<br>Former: 0.87, (0.74-1.03)<br>Never: 0.96, (0.88-1.05)	Current, Former, Non-Drinker	RR, 95%CI<br>Current: 1.16, (1.07-1.27)<br>Former: 0.87, (0.68-1.12)<br>Never: 0.89, (0.81-0.96)	Sociodemographic, Health Behaviour
Liu & Su, 2016[41]	Smoking (Yes/ No)	β= 0.042, SE=0.03, p-value: 0.163<br>No: Ref	Alcohol (Yes/ No)	β=0.201, SE: 0.03, p-value < 0.001<br>No: Ref	Sociodemographic, Health Behaviour, Attitude & Social Environment

For peer review only - http://bmjopen.bmj.com/site/about/guidelines.xhtml

Newman et al., 2003[42]	Self-Reported Pack-years:<br>None<br>=<10<br>>10-20<br>>20-40<br>>40	Proportion of SY, 95% CI<br>Current Smoker: 0.77, (0.65-0.90)<br>(multiplication factor)<br>=<10 : 0.84 (0.73-0.98)<br>>10-20 : 0.90 (0.76-1.07)<br>>20-40 : 0.99 (0.85-1.15)<br>>40 : 0.82 (0.71-0.95)<br>None: Ref	-	-	Sociodemographic, Economic, Health Behaviour, Diseases and Physical Measurements
Newson et al., 2010[43]	Never, Former or Current	Survival Morbidity free vs non-survival, OR, 95% CI:<br>Age and sex-adjusted model: 1.38, (1.07, 1.77)<br>Full model: 1.26, (0.97, 1.65)	average consumption(gr)/day	not statistically significant	Sociodemographic, Economic, Health Behaviour, Diseases and Physical Measurements, Attitude & Social Environment
Pruchno & Wilson-Genderson, 2015[44]	Current Smoker (Yes, No)	Successful: Ref<br>Unsuccessful: b=0.55, SD=0.26, 95% CI=(1.03, 2.93), exp(b)=1.74<br>Subjective only: b=0.32, SD=0.25, 95% CI=(0.83, 2.27), exp(b)=1.38<br>Objective only: b=0.24, SD=0.23, 95% CI=(0.81, 1.99), exp(b)=1.27	Days per week where have at least one drink of alcohol (0-7)	Successful: Ref<br>Unsuccessful: b=-0.09, SD=0.08, 95% CI=(0.83, 1.00), exp(b)=0.91<br>Subjective only: b=-0.02, SD=0.04, 95% CI=(0.90, 1.06), exp(b)=0.98<br>Objective only: b=0.01, SD=0.03, 95% CI=(0.94, 1.08), exp(b)=1.01	Sociodemographic, Economic, Health Behaviour, Attitude & Social Environment
Reed et al., 1998[45]	Cigarette pack-years = (usual number of cigarettes/day) * (number of years)	OR, 95% CI<br>Healthy vs Illness With Impairment: 0.52, (0.39-0.70)<br>Healthy vs Illness Without Impairment: 0.46, (0.33-0.64)<br>Healthy vs Impairment Without Illness: -<br><br>Survival Ratio, 95%CI: 0.72, (0.65-0.79)	monthly intake of beer, wine, and liquor (ml of ethanol/day).	-	Sociodemographic, Health Behaviour, Diseases and Physical Measurement, Model Characteristics
Sabia et al., 2012[46]	Current or Former smoker, Never	OR, 95% CI<br>Never Smoker: 1.29, (1.11, 1.49)<br>Former or Current Smoker: Ref	Abstinence: no alcohol in the last week<br>Moderate: 1–14 units/wk women; 1–21 units/wk men	OR, 95% CI<br>Moderate : 1.31, (1.12, 1.53)<br>No Moderate: Ref	Sociodemographic, Economic, Health Behaviour

For peer review only - http://bmjopen.bmj.com/site/about/guidelines.xhtml

			Heavy: ≥15 units/wk women; ≥21 units/wk men.		
Sarnak et al., 2008[47]	Current, Former, Never	HR, 95% CI<br>Current: 1.73, (0.79, 1.80)<br>Former: 1.21, (0.92, 1.58)<br>Never: Ref	-	-	Sociodemographic, Diseases and Physical Measurements
Shields & Martel, 2006[48]	Current daily smokers, had quit daily smoking within the past 15 years, had quit at least 15 years ago or had never smoked every day	HR, 95% CI<br>Current: 0.7, (0.5, 1.0)<br>Quit during past 15 yrs: 0.7, (0.5, 0.9)<br>Never smoked/ Quit for 15+ years: Ref	Weekly/ Occasional drinker, Non-drinker	HR, 95% CI<br>Weekly/ Occasional drinker: 1.4, (1.1, 1.8)<br>Non-drinker: Ref	Sociodemographic, Economic, Health Behaviour, Attitude & Social Environment
Sun et al., 2011[49]	-	-	Alcohol Intake Levels (g/d)<br>i. 0<br>ii. ≤5.0<br>iii. 5.1–15.0<br>iv. 15.1–30.0<br>v. 30.1–45.0	Fully adj. OR, 95% CI<br>i. 0 : Ref<br>ii. ≤5.0 : 1.11 (0.96–1.29)<br>iii. 5.1–15.0 : 1.19 (1.01–1.40)<br>iv. 15.1–30.0: 1.28 (1.03–1.58)<br>v. 30.1–45.0 : 1.24 (0.87–1.76)<br><br>Age adj. OR, 95% CI<br>i. 0 : Ref<br>ii. ≤5.0 : 1.12 (0.97–1.29)<br>iii. 5.1–15.0 : 1.22 (1.05–1.43)<br>iv. 15.1–30.0: 1.26 (1.03–1.53)<br>v. 30.1–45.0 : 1.05 (0.75–1.46)	Sociodemographic, Economic, Health Behaviour, Diseases and Physical Measurements

For peer review only

For peer review only - http://bmjopen.bmj.com/site/about/guidelines.xhtml

Tampubolon, 2016[50]	Current, No current	Baseline Model, Annual Rate of Phenotypic Decline: b: -0.679, 95%CI: (-0.852, -0.507)<br>Gender Interaction Model, Annual Rate of Phenotypic Decline: b: -0.685, 95%CI: (−0.858,−0.513)	Drink daily<br>Less than daily: Ref	Baseline Model, Annual Rate of Phenotypic Decline: b=0.414, 95%CI=(0.309, 0.519)<br>Gender Interaction Model, Annual Rate of Phenotypic Decline: b=0.412, 95%CI=(0.307, 0.517)	Baseline Model: Sociodemographic, Economic, Health Behaviour, Diseases and Physical Measurements
Terry et al., 2005[51]	Present (smoked within the year before any baseline examination), Absent	OR, 95%CI: 0.51, (0.41, 0.63)	-	-	Sociodemographic, Economic, Health Behaviour, Diseases and Physical Measurement
Vaillant & Mukamal, 2001[52]	Smoking < 30 pack-years (from age 15-50)<br>Smoking ≥ 30 pack-years (from age 15-50)	OR, 95%CI<br>Happy-Well Men vs Sad-Sick or Prematurely Dead<br>Smoking <30 pack-years: College Men at Age 75–80, 4.81, (0.84, 27.7)<br>Smoking <30 pack-years: Core-City Men at Age 65–70, 4.56, (2.29, 9.11)<br>Smoking in ≥30 pack-years: Ref	Alcohol Abuse Absent (Yes, No)	OR, 95%CI<br>Happy-Well Men vs Sad-Sick or Prematurely Dead<br>No alcohol abuse: College Men at Age 75–80, -<br>No alcohol abuse: Core-City Men at Age 65–70, 1.11, (0.53–2.35)	Sociodemographic, Diseases and Physical Measurement, Health Behaviour, Attitude and Social Environment
Vaillant & Western, 2001[53]	0-30 pack-years, ≥ 30 pack-years	OR, 95% CI<br>Happy-Well vs Sad-Sick and Prematurely Dead<br>Univariate Model: Smoking < 30 pack-years: 5.08 (2.85, 9.0)<br>Multivariate Model: Smoking < 30 pack-years: 5.00 (2.38, 10.5)	Alcohol Abuse Absent (Yes, No)	OR, 95%CI<br>Happy-Well vs Sad-Sick and Prematurely Dead<br>Univariate Model: No Alcohol abuse: 2.64 (1.45, 4.81)<br>Multivariate Model: No Alcohol abuse: 0.94 (0.72, 2.05).	Univariate: Diseases and Physical Measurements<br>Multivariate: Sociodemographic, Economic, Health Behaviour, Diseases and Physical Measurement, Attitude and Social Environment

For peer review only - http://bmjopen.bmj.com/site/about/guidelines.xhtml

Willcox et al., 2006[54]	Ever smoker, Never smoker	OR, 95%CI<br>Usual Survival vs Exceptional Survival<br>Age-adj. : 1.27 (1.06-1.53),<br>Fully-adj. : 1.23 (1.01-1.50)<br>Never Smokers: Ref	3 or more drinks/day, less than 3 drinks/day	OR, 95%CI<br>Usual Survival vs Exceptional Survival<br>Age-adj. : 1.84 (1.29-2.62) ,<br>Fully-adj.: 1.61 (1.11-2.34)<br>Less than 3 drinks/d: Ref	Sociodemographic

For peer review only

Table 3: Results of meta-analysis-Healthy Ageing compared to Smoking and Alcohol Consumption

Analysis	Number of Studies	ORs and 95%CI	p-value	I^2	Egger Bias; p-value	Trim-and-fill ORs and 95%CI	Number of Filled Studies
Never vs Current Smokers	7*	2.36 (2.03-2.75)	<.001	43.3%	1.42; .060	2.11 (1.82-2.45)	10
Never vs Former Smokers	5†	1.32 (1.23-1.41)	<.001	32.8%	-0.09; .935	unchanged	-
Past/Never vs Current	6‡	1.72 (1.20-2.47)	.003	87.2%	3.55; .142	1.20 (0.83-1.73)	9
Never vs Past/Current	5§	1.29 (1.16-1.43)	<.001	0.0%	1.08; .157	1.27 (1.14-1.40)	9
Drinkers vs Non-Drinkers	5‖	1.28 (1.08-1.52)	.004	72.1%	-1.25; .350	unchanged	-
Light vs Non-Drinkers	3¶	1.12 (1.03-1.22)	.010	0.0%	-8.17; .043	unchanged	-
Moderate vs Non- Drinkers	4**	1.35 (0.93-1.97)	.112	71.4%	1.77; .549	1.10 (0.77-1.57)	6
High to Non-Drinkers	3††	1.25 (1.09-1.44)	.002	0.0%	-0.63; .828	unchanged	-

OR: Odds Ratio; CI: Confidence Interval

* Bell et al., 2014[28]; Britton et al., 2008 (Males)[29]; Britton et al., 2008 (Females)[29]; Hodge, English et al., 2013[36]; Hodge, O'Dea et al., 2014[37]; LaCroix et al., 2016 (veterans)[39]; LaCroix et al., 2016 (non-veterans)[39]

† Bell et al., 2014[28]; Hodge, English et al., 2013[36]; Hodge, O'Dea et al., 2014[37]; LaCroix et al., 2016 (veterans)[39]; LaCroix et al., 2016 (non-veterans)[39]

‡ Ford et al., 2000[31]; Gu et al., 2009[32]; Guralnik & Kaplan, 1989[33]; Hamer et al., 2013[35]; Pruchno & Wilson-Genderson, 2015[44]; Terry et al., 2005[51]

§ Kaplan et al., 2008[38]; Newson et al., 2010[43]; Gureje et al., 2014[34]; Sabia et al., 2012[46]; Willcox et al., 2006[54]

|| Ford et al., 2000[31]; Gu et al, 2009[32]; Gureje et al., 2014[34]; LaCroix et al., 2016 (veterans)[39]; LaCroix et al., 2016 (non-veterans)[39]

¶ Hodge, English et al., 2013[36]; Hodge, O'Dea et al., 2014[37]; Sun et al., 2011[49]

** Britton et al., 2008 (Males)[29]; Britton et al., 2008 (Females)[29]; Guralnik & Kaplan, 1989[33]; Sun et al., 2011[49]

†† Hodge, English et al., 2013[36]; Hodge, O'Dea et al., 2014[37]; Sun et al., 2011[49]

For peer review only

For peer review only - http://bmjopen.bmj.com/site/about/guidelines.xhtml

List of figure legends

Figure 1: Selection Process Revised

Figure 2: Areas of information included in the definition of healthy ageing

Figure 3: Smoking and Healthy Ageing Forest Plot

Figure 4: Alcohol and Healthy Ageing Forest Plot

For peer review only

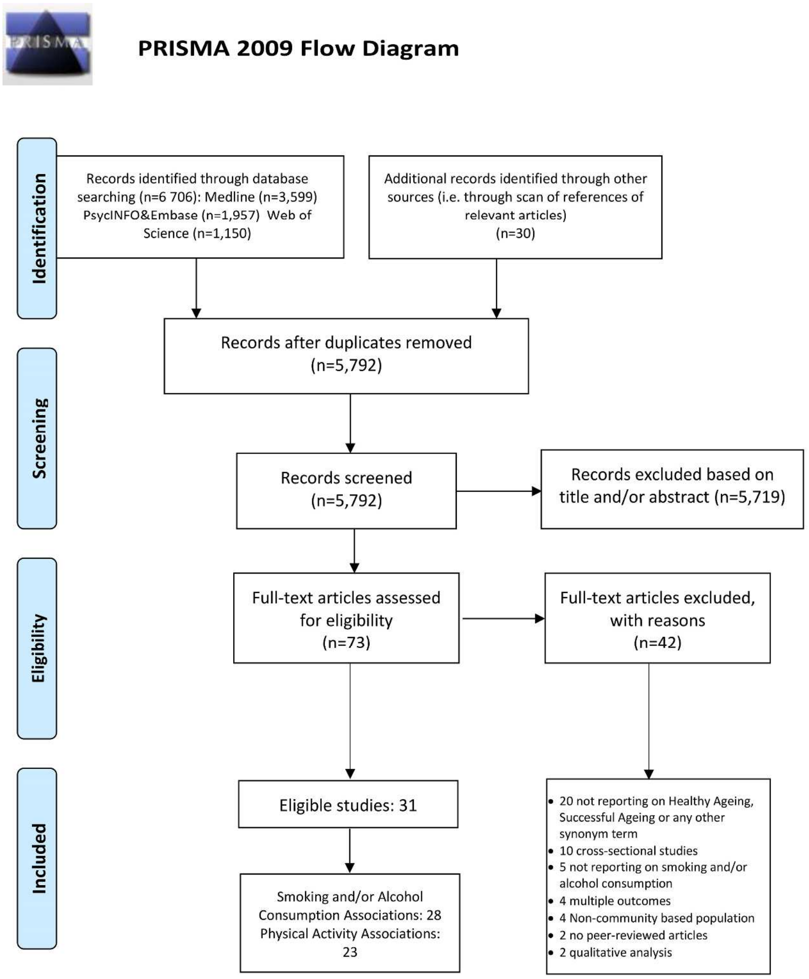

From: Moher D, Liberati A, Tetzlaff J, Altman DG, The PRISMA Group (2009). *P*referred *R*eporting *I*tems for *S*ystematic Reviews and *M*eta-*A*nalyses: The PRISMA Statement. PLoS Med 6(7): e1000097. doi:10.1371/journal.pmed1000097

For more information, visit www.prisma-statement.org.

Figure 1: Selection process-PRISMA flow diagram

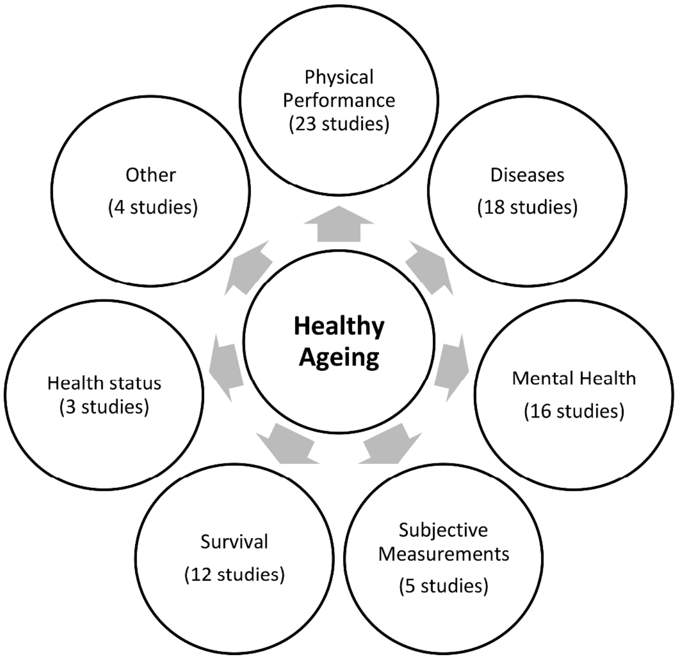

Figure 2: Areas of information included in the definition of healthy ageing

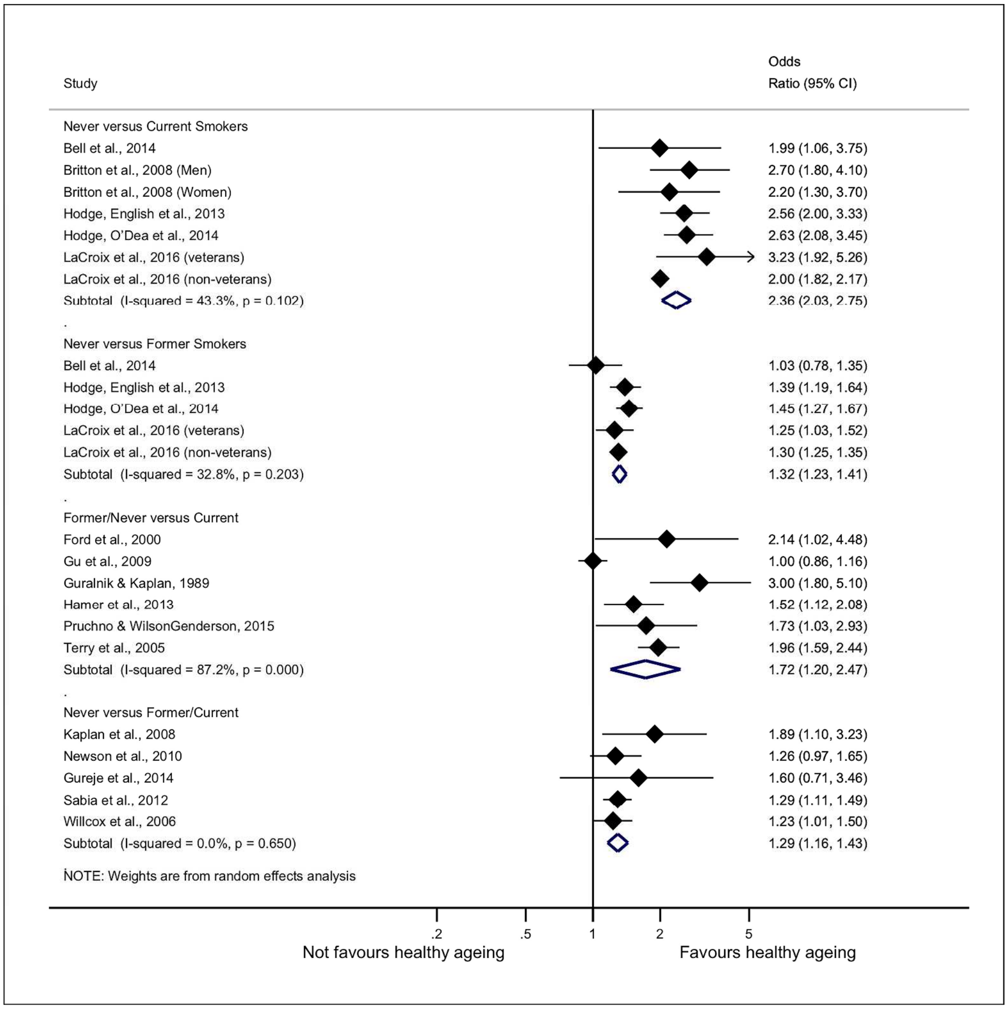

Figure 3: Smoking and healthy ageing forest plot

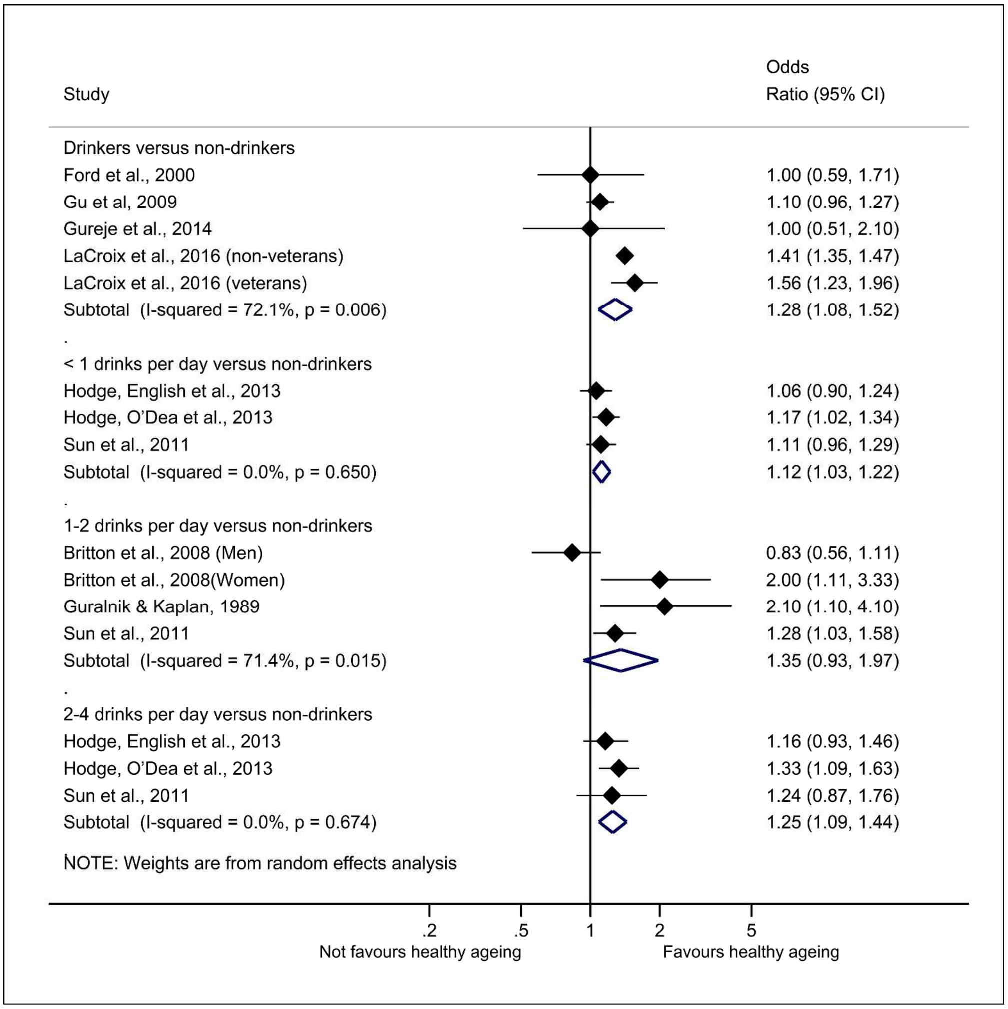

Figure 4: Alcohol and healthy ageing forest plot

MOOSE Checklist for Meta-analyses of Observational Studies

Item No	Recommendation		Reported on Page No
Reporting of background should include			
1	Problem definition	To address the need for more accurate estimates of the relationship between smoking and healthy ageing and alcohol consumption and healthy ageing (p. 6)	
2	Hypothesis statement	In systematic reviews focusing on elderly people, smoking is associated with premature mortality and an increased incidence of Alzheimer's disease. Studies showed that light to moderate alcohol consumption is associated with a decrease in dementia and cognitive incidence, as well as with a reduced risk for cardiovascular disease mortality, incident coronary heart disease, incident stroke, stroke mortality and all-cause mortality(p. 5)	
3	Description of study outcome(s)	The primary outcome of this review was health status measured by healthy ageing, and any other term related to it (e.g. successful ageing, active ageing, healthy survival etc.) (p. 7)	
4	Type of exposure or intervention used	Eligible studies had to meet the following criteria: i. being published in an electronic journal article; ii. constitute an original peer-reviewed longitudinal study; and iii. report any kind of longitudinal association between smoking and/or alcohol consumption and healthy ageing (p. 7)	
5	Type of study designs used	longitudinal study (p. 7)	
6	Study population	Studies that included cohorts of individuals who were institutionalised or hospitalised and animal studies were also excluded (p. 7)	
Reporting of search strategy should include			
7	Qualifications of searchers (eg, librarians and investigators)	Credentials are indicated in the title page	
8	Search strategy, including time period included in the synthesis and key words	Search strategy and selection criteria (p.6-7) and Appendix 3.docx	
9	Effort to include all available studies, including contact with authors	In case that full text could not be retrieved, the corresponding author of the paper was contacted (p. 7).<br>Other relevant systematic reviews of healthy ageing and reference lists of the eligible studies were also searched (p.7)	
10	Databases and registries searched	MEDLINE (PubMed interface), EMBASE (OVID interface), Psychinfo (OVID interface) and CENTRAL (Cochrane Central Register of Controlled Trials) (p. 6-7)	
11	Search software used, name and version, including special features used (eg, explosion)	EndNote (ENDNOTE X7, Thomson Reuters), (p. 7)	
12	Use of hand searching (eg, reference lists of obtained articles)	Other relevant systematic reviews of healthy ageing and reference lists of the eligible studies have also been searched (p. 7)	
13	List of citations located and those excluded, including justification	Details of the literature search are outlined in the PRISMA flow-chart: Figure 1. The citation list is available upon request.	
14	Method of addressing articles published in languages other than English	Contributors (p. 18)	
15	Method of handling abstracts and unpublished studies	exclusion of papers that were abstracts, conference papers, cross-sectional studies (p. 9-10)	
16	Description of any contact with authors	we planned to contact authors for any missing data; however, this was not needed.	

For peer review only - http://bmjopen.bmj.com/site/about/guidelines.xhtml

Reporting of methods should include		
17	Description of relevance or appropriateness of studies assembled for assessing the hypothesis to be tested	Search strategy and selection criteria paragraph (p. 6-7)
18	Rationale for the selection and coding of data (eg, sound clinical principles or convenience)	Data extraction and quality assessment paragraph (p. 8) and Table 1 and Table 2. We extracted data from each study that were relevant to the population, the exposure, the outcome and any confounder.
19	Documentation of how data were classified and coded (eg, multiple raters, blinding and interrater reliability)	Data extraction and quality assessment paragraph (p. 8).
20	Assessment of confounding (eg, comparability of cases and controls in studies where appropriate)	We extracted information regarding the different confounders that were used per study (Table 2)
21	Assessment of study quality, including blinding of quality assessors, stratification or regression on possible predictors of study results	Two investigators assessed study quality by using the QUIPS tool (Quality Assessment paragraph, p.11 and Table A2)
22	Assessment of heterogeneity	Heterogeneity was assessed by the I^2 (Data Analysis paragraph, p. 9)
23	Description of statistical methods (eg, complete description of fixed or random effects models, justification of whether the chosen models account for predictors of study results, dose-response models, or cumulative meta-analysis) in sufficient detail to be replicated	random effects meta-analysis was used (p. 9)
24	Provision of appropriate tables and graphics	We provided the PRISMA flow-chart, tables with the characteristics of studies, table with the results of meta-analysis and forest plots
Reporting of results should include		
25	Graphic summarizing individual study estimates and overall estimate	Figure 2 and Figure 3
26	Table giving descriptive information for each study included	Table 1 and Table 2
27	Results of sensitivity testing (eg, subgroup analysis)	Data did not allow to perform subgroup analysis
28	Indication of statistical uncertainty of findings	95% confidence intervals were presented with all estimators, I^2 values, and results of the trim-and-fill algorithm. Table 3.

Item No	Recommendation	Reported on Page No
Reporting of discussion should include		
29	Quantitative assessment of bias (eg, publication bias)	Publication bias was assessed Egger test and the trim-and-fill algorithm (Table 3)
30	Justification for exclusion (eg, exclusion of non-English language citations)	We did not exclude any study due to language. We excluded studies for the meta-analysis because they did not report a relevant statistic to OR or because alcohol conversion to comparable metrics was not possible
31	Assessment of quality of included studies	Data extraction and quality assessment paragraph (p. 8) and Table A2.
Reporting of conclusions should include		
32	Consideration of alternative explanations for observed results	Limitations are discussed in parag. Strengths and Limitations (p. 14). The most important are: adjustment by different use of covariates, different follow-up time and attrition rate, self-reported questionnaires and inconsistency in the way smoking/alcohol have been measured among the different cohorts.
33	Generalization of the conclusions (ie, appropriate for the data presented and within the domain of the literature review)	The positive impact of smoking abstinence and smoking cessation on healthy ageing is identified in both the majority of our primary studies and in our pooled effect estimate as well (even when we adjusted for publication bias). A positive relationship between limited

For peer review only - http://bmjopen.bmj.com/site/about/guidelines.xhtml

		consumption of drinking could also be argued but more research is needed.
34	Guidelines for future research	Conclusion parag. (p. 17)
35	Disclosure of funding source	Funding parag. (p.18)

From: Stroup DF, Berlin JA, Morton SC, et al, for the Meta-analysis Of Observational Studies in Epidemiology (MOOSE) Group. Meta-analysis of Observational Studies in Epidemiology. A Proposal for Reporting. *JAMA*. 2000;283(15):2008-2012. doi: 10.1001/jama.283.15.2008.
Transcribed from the original paper within the NEUROSURGERY® Editorial Office, Atlanta, GA, United Sates. August 2012.

For peer review only

University of York
Centre for Reviews and Dissemination

NHS
National Institute for Health Research

PROSPERO International prospective register of systematic reviews

ATHLOS: impact of physical activity, smoking and alcohol on healthy ageing throughout the lifespan: protocol for a systematic review

Christina Daskalopoulou, Matthew Prina, Martin Prince, Artemis Koukounari

Citation

Christina Daskalopoulou, Matthew Prina, Martin Prince, Artemis Koukounari. ATHLOS: impact of physical activity, smoking and alcohol on healthy ageing throughout the lifespan: protocol for a systematic review. PROSPERO 2016:CRD42016038130 Available from http://www.crd.york.ac.uk/PROSPERO_REBRANDING/display_record.asp?ID=CRD42016038130

Review question(s)

What is the impact of physical activity, smoking and alcohol consumption across the life span on healthy ageing?

Quantification and summary of the relationship between the aforementioned lifestyle factors and healthy ageing by using a meta-analytical approach.

Searches

The literature search will include any related term or synonym of healthy ageing and text words related to physical activity, smoking and alcohol. Where possible, the latter will be developed using medical subject headings (MeSH). We will search MEDLINE (PubMed interface), EMBASE (OVID interface), PsycINFO (OVID interface), Web of Science and CENTRAL (Cochrane Central Register of Controlled Trials).

In addition, scanning of the reference lists of the included studies will take place so as to identify any relevant studies that were not included by the initial research. Finally, authors of relevant studies will be contacted in case that supplementary information is required.

Types of study to be included

This review will be restricted to original studies (peer-reviewed).

Opinion pieces, books, letters, editorials, abstracts, dissertations/thesis and/or conference proceedings will not be considered.

Condition or domain being studied

Health status measured by healthy ageing.

Participants/ population

Eligible studies will include people, who are community-based, across the whole life span. Hospitalised or institutionalised populations will not been considered. Participants being disabled or diagnosed with dementia, frailty or any chronic disease will be included in the systematic review as long as they are community-based. There will be no other restrictions, for instance gender, age, education level, occupation and/or ethnicity.

Intervention(s), exposure(s)

Associations of either physical activity, smoking and/or alcohol consumption to one of the outcomes of healthy ageing, irrespectively of the way the latter is being measured/defined.

Comparator(s)/ control

Not applicable.

Context

Community-based populations and not clinical ones.

For peer review only - http://bmjopen.bmj.com/site/about/guidelines.xhtml

Outcome(s)

Primary outcomes

Health status measured by healthy ageing.

Secondary outcomes

Not applicable.

Data extraction, (selection and coding)

An EndNote (ENDNOTE X7, Thomson Reuters) library will be created so as to store all the studies retrieved by the electronic databases. Using EndNote's auto-deduplication function, duplicate citations will be removed. Since auto-deduplication is thought to be only partially successful (Qi et al., 2013), the remaining duplicates will be identified by hand-searching techniques. To do this, references will be alphabetically ordered according to the first authors' names and thereafter according to their titles.

Studies from the electronic database searches will be initially determined as relevant or not by title and abstract screening. References will be characterised as "Not Eligible" and "Potentially Eligible" by screening the title and the abstract of the study.

In order to include a study in the "Potential Eligible" folder the following criteria will be applied:

Study characteristics: "potentially eligible studies" have to be published in an electronic journal article and constitute an original peer-reviewed longitudinal study. We will include studies that examined healthy ageing as an outcome and that reported on the following determinants: physical activity, smoking and/or alcohol consumption.

Study participants: to be "potentially eligible", the title or abstract of the study must clearly state that the study population is an observational study/cohort study and that people are not hospitalised or belong to a specific clinical population. Participants being disabled or diagnosed with dementia, frailty or any chronic disease will be included in the systematic review as long as they are community based. There will be no other restrictions.

Interventions: To be "potentially eligible", the title or abstract must clearly state that the study reports the associations of either physical activity, smoking and/or alcohol consumption to one of the outcomes of healthy ageing, irrespectively of the way the latter is being measured/defined.

The final inclusion will be based on full text screening. Full text studies of "potentially eligible" references will be retrieved and assessed for their final inclusion by the first reviewer and a second researcher. A random sample of those, accounting for the 5% of the total studies, will be checked by a senior epidemiologist. Once the screening process is completed, the creation of a PRISMA flow diagram will be generated so as to illustrate the study selection process.

Risk of bias (quality) assessment

The risk of bias in primary studies will be assessed by using the Quality in Prognosis Studies (QUIPS) tool (Hayden et al., 2013). Validity and bias should be assessed by considering the following 6 areas: participation, attrition, prognostic factor measurement, confounding measurement and account, outcome measurement and analysis and reporting. During the first step, the 6 aforementioned potential sources of bias will be classified as either "yes", "partly", "no" or "unsure" by the first reviewer. Each study will then be characterised with an overall risk bias rating of "high", "moderate" or "low".

This characterisation will be defined as follows: "low" risk of bias will have these studies which fulfil all or the majority of the 6 QUIPS tool domains (no source of bias should be characterised as "yes" and at most one "partly"). "Moderate" risk of bias will have the studies that fullfil some of the aforementioned criteria; and "high" risk of bias will have those that meet few or none of the criteria.

During the next step, the first reviewer and the senior epidemiologist will judge if any of the "high" and "moderate" level studies present fatal flaws or considerable amount of bias and hence, in order to avoid reaching any misleading conclusion, it would be better to exclude these studies from the results (Sterne et al., 2001). The reasons based on which any studies have been excluded will be provided with full detail in a supplementary table.

For peer review only - http://bmjopen.bmj.com/site/about/guidelines.xhtml

For peer review only

UNIVERSITY of York
Centre for Reviews and Dissemination

National Institute for Health Research

Publication bias will be assessed for syntheses including five or more studies using a funnel plot and the Egger regression asymmetry test.

Strategy for data synthesis

The impact of physical activity, smoking and alcohol consumption on healthy ageing across the lifespan will be presented. In particular, the different levels of these factors and how these are related to healthy ageing will be extracted.

We will consider the different levels/measurements of these factors as statistically significant if i) the reported P-value is less than 0.05; ii) the author reported that there was a significant association; and iii) the 95% confidence interval around odds ratio or a similar statistic did not include 1.

Studies that refer no impact of the aforementioned determinants will also be presented. In addition, any concerns regarding the generalisability of the findings will be mentioned. For example, a small sample size, ethnicity/gender bias or important number of participants that were lost to follow-up will be referred.

Assuming non-substantial heterogeneity in study characteristics (e.g. determinant measurements, outcomes, etc.), we will carry out a meta-analysis to pool together effect estimates for each determinant. I-squared statistic will be used in order to assess the heterogeneity of the studies and based on this and the theoretical ground of the included studies, a fixed or random effect meta-analysis will be performed (Higgins et al., 2011). If a meta-analysis is not feasible due to amplified heterogeneity for the outcome and/or the lifestyle determinant measurement, a narrative synthesis for summarising the evidence will be performed.

In case of any discrepancies among the studies, study designs, populations, precision and publication bias will be assessed. Quality of our evidence will be considered as high when further research is very unlikely to change our confidence in the estimate of effect, moderate when further research is likely to have a considerable impact on our confidence and may change our estimate. The quality of evidence will be considered as low, in case that further research is very likely to have an important impact on our confidence in the estimate of effect, and is likely to change the estimate; and very low, when we are not certain about the estimate effect.

Quality of our findings will be assessed by performing a sensitivity analysis, should the selected studies permit it. For example, we will repeat the meta-analysis by excluding studies with low quality and see what will be the impact of it to our conclusions.

Analysis of subgroups or subsets

If the selected data permit, sub-group analyses will be carried out so as to stratify for different physical activity measurements (i.e subjective/self-report vs objective assessment of PA).

Dissemination plans

The findings of this review will be presented to relevant scientific conferences, for example ATHLOS consortium workshops, as well as to academic journals. Furthermore, conclusions will be disseminated through research and university networks.

Contact details for further information

Christina Daskalopoulou

King's College London, Institute of Psychiatry, Psychology & Neuroscience, Centre for Global Mental Health, Department of Health Services and Population Research, David Goldberg Centre, De Crespigny Park, Denmark Hill, London SE5 8AF, UK.

christina.daskalopoulou@kcl.ac.uk

Organisational affiliation of the review

King's College London, Institute of Psychiatry, Psychology & Neuroscience, Centre for Global Mental Health, Department of Health Services and Population Research

For peer review only - http://bmjopen.bmj.com/site/about/guidelines.xhtml

http://www.kcl.ac.uk/ioppn/depts/hspr/index.aspx

Review team

Ms Christina Daskalopoulou, King's College London
Dr Matthew Prina, King's College London
Professor Martin Prince, King's College London
Dr Artemis Koukounari, King's College London

Collaborators

Dr Carolina Kralj, King's College London

Anticipated or actual start date

14 March 2016

Anticipated completion date

31 December 2016

Funding sources/sponsors

Department of Health Services and Population Research, Institute of Psychology, Psychiatry and Neuroscience at King's College London is the Sponsor, meaning that it has overall control of the data. This project falls under the ATHLOS (Ageing Trajectories of Health: Longitudinal Opportunities and Synergies) project, funded by the European Union's Horizon 2020 Research and Innovation Programme under grant agreement number 63531.

Conflicts of interest

None known

Language

English

Country

England

Subject index terms status

Subject indexing assigned by CRD

Subject index terms

Age Factors; Aging; Alcohol Drinking; Exercise; Health; Health Status; Humans; Motor Activity; Risk Factors; Smoking

Reference and/or URL for protocol

http://www.crd.york.ac.uk/PROSPEROFILES/38130_PROTOCOL_20160326.pdf

Stage of review

Ongoing

Date of registration in PROSPERO

26 April 2016

Date of publication of this revision

26 April 2016

Stage of review at time of this submission	Started	Completed
Preliminary searches	Yes	No
Piloting of the study selection process	Yes	No

For peer review only

UNIVERSITY of York
Centre for Reviews and Dissemination

NHS
National Institute for Health Research

Formal screening of search results against eligibility criteria	No	No
Data extraction	No	No
Risk of bias (quality) assessment	No	No
Data analysis	No	No

PROSPERO
International prospective register of systematic reviews
The information in this record has been provided by the named contact for this review. CRD has accepted this information in good faith and registered the review in PROSPERO. CRD bears no responsibility or liability for the content of this registration record, any associated files or external websites.

For peer review only

For peer review only - http://bmjopen.bmj.com/site/about/guidelines.xhtml

Systematic Review Protocol: ATHLOS: Impact of Physical Activity, Smoking and Alcohol on Healthy Ageing throughout the Life Span: protocol for a systematic review.

Embase, Psychinfo (OVID interface) and Cochrane Library

(health* ageing) OR (health* aging) OR (successful ageing) OR (successful aging) OR (positive ageing) OR (positive aging) OR (productive ageing) OR (productive aging) OR (ageing well) OR (aging well) OR (optim* ageing) OR (optim* aging) OR (unimpaired ageing) OR (unimpaired aging) OR (effective ageing) OR (effective aging) OR (robust ageing) OR (robust aging) OR (exceptional survival)

AND ((smoking) OR (tobacco) OR (cigarette*) OR (physical activity) OR (physical inactivity) OR (exercise*) OR (alcohol) OR (Alcohol Drinking))

MedLine search (PubMed/PMC)

(("health* ageing") OR ("health* aging") OR ("successful ageing") OR ("successful aging") OR ("positive ageing") OR ("positive aging") OR ("productive ageing") OR ("productive aging") OR ("ageing well") OR ("aging well") OR ("optim* ageing") OR ("optim* aging") OR ("unimpaired ageing") OR ("unimpaired aging") OR ("effective ageing") OR ("effective aging") OR ("robust ageing") OR ("robust aging") OR ("exceptional survival")) AND (("motor activity"[MeSH Terms]) OR ("smoking"[MeSH Terms]) OR ("Alcohol Drinking"[MesH Terms]))

Table A1: Areas of information included in the definition of healthy ageing

Authors	Survival	Health Status	Physical Performance	Diseases	Mental Health	Subjective Measurements	Others
Andrews et al., 2002			x		x		
Bell et al., 2014	x		x	x	x		
Britton et al., 2008		x	x	x	x		
Burke et al., 2001				x			
Ford et al., 2000							x
Gu et al., 2009	x		x				
Guralnik & Kaplan, 1989			x				
Gureje et al., 2014			x	x		x	
Hamer et al., 2013			x	x	x		
Hodge, English et al., 2013	x		x	x	x		
Hodge, O'Dea et al., 2014	x		x	x	x		
Kaplan et al., 2008		x	x				
LaCroix et al., 2016	x		x	x			
Li et al., 2001			x		x	x	
Liu & Su, 2016			x	x	x		
Newman et al., 2003			x	x	x		
Newson et al., 2010	x			x			
Pruchno & Wilson-Genderson, 2015			x	x		x	
Reed et al., 1998	x		x	x	x		
Sabia et al., 2012			x	x	x		
Sarnak et al., 2008			x	x	x		
Shields & Martel, 2006		x	x		x		
Sun et al., 2011	x		x	x	x		
Tampubolon, 2016							x
Terry et al., 2005	x			x	x		
Vaillant & Mukamal, 2001	x		x			x	x
Vaillant & Western, 2001	x		x			x	x
Willcox et al., 2006	x		x	x	x		
**Areas of information included in the definition of Healthy Ageing**	**12**	**3**	**23**	**18**	**16**	**5**	**4**

For peer review only

Table A2: Quality assessment of the studies – aggregated results of QUIPS tool

Author, Year of Publication	The study sample represents the population of interest on key characteristics, sufficient to limit potential bias of the observed relationship between PF and outcome.	Loss to follow-up is not associated with key characteristics sufficient to limit potential bias to the observed relationship between PF and outcome.	PF is adequately measured in study participants to sufficiently limit potential bias.	Outcome of interest is adequately measured in study participants to sufficiently limit potential bias.	Important potential confounders are appropriately accounted for, limiting potential bias with respect to the relationship between PF and outcome.	The statistical analysis is appropriate for the design of the study, limiting potential for presentation of invalid or spurious results.	Total
Andrews et al., 2002	Low Bias	Moderate Bias	Moderate Bias	Low Bias	Moderate Bias	Low Bias	Moderate Bias
Bell et al., 2014	Low Bias	Low Bias	Low Bias	Low Bias	Moderate Bias	Low Bias	Low Bias
Britton et al., 2008	Low Bias	Moderate Bias	Low Bias	Low Bias	Moderate Bias	Low Bias	Low Bias
Burke et al., 2001	Low Bias	Low Bias	Low Bias	Moderate Bias	Moderate Bias	Low Bias	Low Bias
Ford et al., 2000	Low Bias	Moderate Bias	Moderate Bias	Moderate Bias	Low Bias	Low Bias	Moderate Bias
Gu et al., 2009	Low Bias	Low Bias	Low Bias	Low Bias	Moderate Bias	Low Bias	Low Bias
Guralnik & Kaplan, 1989	Low Bias	Moderate Bias	Moderate Bias	Moderate Bias	Moderate Bias	Low Bias	Moderate Bias
Gureje et al., 2014	Low Bias	Moderate Bias	Low Bias	Low Bias	Moderate Bias	Low Bias	Low Bias
Hamer et al., 2013	Low Bias	Low Bias	Moderate Bias	Low Bias	Low Bias	Low Bias	Low Bias
Hodge, English et al., 2013	Low Bias	Moderate Bias	Low Bias	Low Bias	Low Bias	Low Bias	Low Bias
Hodge, O'Dea et al., 2014	Low Bias	High Bias	Low Bias	Low Bias	Low Bias	Low Bias	Moderate Bias
Kaplan et al., 2008	Low Bias	Moderate Bias	Low Bias	Moderate Bias	Low Bias	Low Bias	Low Bias
LaCroix et al., 2016	Low Bias	Moderate Bias	Low Bias	Low Bias	Moderate Bias	Low Bias	Low Bias
Li et al., 2001	Low Bias	High Bias	Low Bias	Low Bias	Moderate Bias	Low Bias	Moderate Bias
Liu & Su, 2016	Low Bias	Moderate Bias	Low Bias	Low Bias	Moderate Bias	Low Bias	Low Bias
Newman et al., 2003	Low Bias	Low Bias	Low Bias	Low Bias	Moderate Bias	Low Bias	Low Bias
Newson et al.,	Low Bias	Moderate Bias	Low Bias	Low Bias	Moderate Bias	Low Bias	Low Bias

For peer review only

2010							
Pruchno & Wilson-Genderson, 2015	Low Bias	Moderate Bias	Low Bias	Low Bias	Moderate Bias	Low Bias	Low Bias
Reed et al., 2011	Low Bias	Moderate Bias	Low Bias	Low Bias	Low Bias	Moderate Bias	Low Bias
Sabia et al., 2012	Low Bias	Low Bias	Low Bias	Moderate Bias	Low Bias	Low Bias	Low Bias
Sarnak et al., 2008	Low Bias	Moderate Bias	Moderate Bias	Low Bias	Moderate Bias	Low Bias	Moderate Bias
Shields & Martel, 2006	Low Bias	Moderate Bias	Low Bias	Low Bias	Low Bias	Low Bias	Low Bias
Sun et al., 2011	Low Bias	Low Bias	Low Bias	Low Bias	Low Bias	Low Bias	Low Bias
Tampubolon, 2016	Low Bias	Low Bias	Low Bias	Low Bias	Moderate Bias	Low Bias	Low Bias
Terry et al., 2005	Low Bias	Low Bias	Low Bias	Moderate Bias	Moderate Bias	Moderate Bias	Moderate Bias
Vaillant & Mukamal, 2001	High Bias	Moderate Bias	Low Bias	Moderate Bias	Moderate Bias	Low Bias	High Bias
Vaillant & Western, 2001	High Bias	Low Bias	Low Bias	Low Bias	Moderate Bias	Low Bias	Moderate Bias
Willcox et al., 2006	Moderate Bias	Moderate Bias	Low Bias	Low Bias	Moderate Bias	Low Bias	Moderate Bias

For peer review only

Table A3: Smoking and alcohol consumption with healthy ageing meta-analyses results

Analysis	Number of Studies	DerSimonian-Laird ORs and 95%CI	p-value	I^2	Paule-Mandel ORs and 95%CI	p-value	I^2
Never vs Current Smokers	7*	2.36 (2.03-2.75)	<.001	43,3%	2.29 (2.03-2.59)	<.001	23,6%
Never vs Former Smokers	5†	1.32 (1.23-1.41)	<.001	32,8%	1.31 (1.21-1.43)	<.001	50,0%
Past/Never vs Current	6‡	1.72 (1.20-2.47)	.003	87,2%	1.69 (1.25-2.29)	<.001	81,3%
Never vs Past/Current	5§	1.29 (1.16-1.43)	<.001	0,0%	1.29 (1.16-1.43)	<.001	0,0%
Drinkers vs Non-Drinkers	5‖	1.28 (1.08-1.52)	.004	72,1%	1.29 (1.10-1.50)	0,002	68,2%
Light vs Non-Drinkers	3¶	1.12 (1.03-1.22)	.010	0,0%	1.12 (1.03-1.22)	0,010	0,0%
Moderate vs Non- Drinkers	4**	1.35 (0.93-1.97)	.112	71,4%	1.37 (0.90-2.08)	0,138	77,0%
High to Non-Drinkers	3††	1.25 (1.09-1.44)	.002	0,0%	1.25 (1.09-1.44)	0,002	0,0%

OR: Odds Ratio; CI: Confidence Interval

* Bell et al., 2014; Britton et al., 2008 (Males); Britton et al., 2008 (Females); Hodge, English et al., 2013; Hodge, O'Dea et al., 2014; LaCroix et al., 2016 (veterans); LaCroix et al., 2016 (non-veterans)

† Bell et al., 2014; Hodge, English et al., 2013; Hodge, O'Dea et al., 2014; LaCroix et al., 2016 (veterans); LaCroix et al., 2016 (non-veterans)

‡ Ford et al., 2000; Gu et al., 2009; Guralnik & Kaplan, 1989; Hamer et al., 2013; Pruchno & Wilson-Genderson, 2015; Terry et al., 2005

§ Kaplan et al., 2008; Newson et al., 2010; Gureje et al., 2014; Sabia et al., 2012; Willcox et al., 2006

|| Ford et al., 2000; Gu et al, 2009; Gureje et al., 2014; LaCroix et al., 2016 (veterans); LaCroix et al., 2016 (non-veterans)

¶ Hodge, English et al., 2013; Hodge, O'Dea et al., 2014; Sun et al., 2011

** Britton et al., 2008 (Males); Britton et al., 2008 (Females); Guralnik & Kaplan, 1989; Sun et al., 2011

†† Hodge, English et al., 2013; Hodge, O'Dea et al., 2014; Sun et al., 2011

For peer review only

BMJ Open

Associations of Smoking and Alcohol Consumption with Healthy Ageing: A Systematic Review and Meta-analysis of longitudinal studies

Journal:	*BMJ Open*
Manuscript ID	bmjopen-2017-019540.R2
Article Type:	Research
Date Submitted by the Author:	22-Feb-2018
Complete List of Authors:	Daskalopoulou, Christina; King's College London, Health Service and Population Research Centre for Global Mental Health & Primary Care Research<br>Stubbs, B; King's College London, Health Service and Population Research Department; South London and Maudsley NHS Foundation Trust, Physiotherapy Department<br>Kralj, Carolina; King's College London, Health Service and Population Research Centre for Global Mental Health & Primary Care Research<br>Koukounari, Artemis; Liverpool School of Tropical Medicine, Department of Clinical Sciences<br>Prince, Martin; King's College London, Health Service and Population Research Centre for Global Mental Health & Primary Care Research<br>Prina, A.Matthew; King's College London, Health Service and Population Research Centre for Global Mental Health & Primary Care Research
**Primary Subject Heading**:	Public health
Secondary Subject Heading:	Global health, Health policy, Public health, Smoking and tobacco
Keywords:	healthy ageing, smoking, alcohol consumption, drinking, meta-analysis

SCHOLARONE™
Manuscripts

Associations of Smoking and Alcohol Consumption with Healthy Ageing: A Systematic Review and Meta-analysis of longitudinal studies

Christina Daskalopoulou, MSc[1], Brendon Stubbs, PhD[2,3], Carolina Kralj, MD[1], Artemis Koukounari, PhD[4], Martin Prince, MD, PhD[1], and A. Matthew Prina, PhD[2]

[*] Address for correspondence: Mrs C. Daskalopoulou, King's College London, Institute of Psychiatry, Psychology and Neuroscience, David Goldberg Centre, De Crespigny Park, London SE5 8AF, UK. (Email: christina.daskalopoulou@kcl.ac.uk.com)

[1] Health Service and Population Research Department, King's College London, Institute of Psychiatry, Psychology and Neuroscience, Centre for Global Mental Health, London, UK;

[2] Health Service and Population Research Department, King's College London, Institute of Psychiatry, Psychology and Neuroscience, London, UK;

[3] Physiotherapy Department, South London and Maudsley NHS Foundation Trust, London, UK;

[4] Department of Clinical Sciences, Liverpool School of Tropical Medicine, Liverpool, UK.

For peer review only - http://bmjopen.bmj.com/site/about/guidelines.xhtml

Abstract (298 words)

Objectives The number of older people is growing across the world, however quantitative synthesis of studies examining the impact of lifestyle factors on the ageing process are rare. We conducted a systematic review and meta-analysis of longitudinal studies to synthesise the associations of smoking and alcohol consumption with healthy ageing (HA).

Methods Major electronic databases were searched from inception to March 2017 (PROSPERO reg. CRD42016038130). Studies were assessed for methodological quality. Random-effect meta-analysis was performed to calculate pooled odds ratios (OR) and 95% confidence intervals (CI).

Results In total, we identified 28 studies (n=184,543); 27 studies reported results on smoking, 22 on alcohol consumption. 23 studies reported a significant positive association of never or former smoking with HA and 4 non-significant. 12 studies reported a significant positive association of alcohol consumption with HA, 9 no association, 1 negative. Meta-analysis revealed increased pooled OR of HA for never smokers compared to current smokers (2.36, 95%CI 2.03-2.75), never smokers compared to former smokers (1.32, 95%CI 1.23-1.41), former or never smokers compared to current smokers (1.72, 95%CI 1.20-2.47), never smokers compared to past or current smokers (1.29, 95%CI 1.16-1.43); drinkers compared to non-drinkers (1.28, 95%CI 1.08-1.52), light drinkers compared to non-drinkers (1.12, 95%CI 1.03-1.22), moderate drinkers compared to non-drinkers (1.35, 95%CI 0.93-1.97) and high drinkers compared to non-drinkers (1.25, 95%CI 1.09-1.44). There was considerable heterogeneity in the definition and measurement of HA and alcohol consumption.

Conclusions There is consistent evidence from longitudinal studies that smoking is negatively associated with HA. The associations of alcohol consumption with HA are

For peer review only - http://bmjopen.bmj.com/site/about/guidelines.xhtml

equivocal. Future research should focus on the implementation of a single metric of HA, on the use of consistent drinking assessment among studies and on a full-range of confounding adjustment. Our research also highlighted the limited research on ageing in low-and-middle income countries.

Key words: healthy ageing; smoking; alcohol consumption; drinking; meta-analysis.

For peer review only

For peer review only - http://bmjopen.bmj.com/site/about/guidelines.xhtml

Strengths and limitations of this study

- This is the first systematic review and meta-analysis trying to quantify the associations of smoking and alcohol consumption with healthy ageing; our findings were also adjusted for publication bias.
- Independent double screening, searching of previous systematic reviews in the field and the reference lists of the eligible studies as well as not excluding non-English studies guarantee that limited number of studies may have not been considered.
- Heterogeneity in the definition and measurement of healthy ageing as well as different confounding adjustment in the initial studies may have influenced our meta-analytical results.
- The number of studies included in the meta-analysis regarding the associations of alcohol consumption was small due to the great heterogeneity in the measurement of it.
- Our findings may not be generalisable due to the limited research in low-and-middle income countries.

For peer review only - http://bmjopen.bmj.com/site/about/guidelines.xhtml

Introduction

Smoking has emerged as the leading contributing factor of many respiratory diseases and one of the most important risk factors for cardiovascular diseases, cancers of several organs and many other pathological conditions.[1] In systematic reviews focusing on older people, smoking is associated with premature mortality[2] and an increased incidence of Alzheimer's disease.[3] Smoking is also linked with a considerable societal cost as smoking-related absenteeism and premature deaths create an additional burden to the healthcare system and to the economy in general.[4] The healthcare cost of tobacco-related diseases accounted for almost 6% of the global health expenditures in 2012.[5]

Tobacco use and harmful use of alcohol are recognised by the United Nations Political Declaration on Non-Communicable Diseases (NCDs) among the most common risk factors.[6] Harmful use of alcohol results approximately in 3.3 million deaths annually (5.9% of all deaths) and alcohol-attributable deaths are the highest for middle-aged people.[7] Older people's sensitivity to the effects of alcohol increases whereas tolerance decreases,[8] meaning that even a low amount of alcohol can create problems in an older person. In addition, older people who drink harmfully often do not present to services due to stigma associated with harmful drinking, and when they do, their symptoms can be easily confused with physical and mental issues associated with ageing.[9] Nevertheless, studies showed that light to moderate alcohol consumption is associated with a decreased risk in dementia,[10] as well as with a reduced risk for cardiovascular disease mortality, incident coronary heart disease, incident stroke, stroke mortality and all-cause mortality.[11]

Drinking and smoking are correlated in the general population[12] with smokers being more likely to drink and vice versa.[13] People who are smokers and heavy drinkers exhibit the highest mortality rate as indicated in a study of middle-aged male participants;[14] another study also showed that men who smoked and drank more than 15 units per week of alcohol

For peer review only - http://bmjopen.bmj.com/site/about/guidelines.xhtml

had almost three times higher all-cause mortality rate compared to non-smokers and non-drinkers.[15] As populations are ageing rapidly, with one in eight people currently aged 60 and over and with these estimates predicted to increase,[16] it is critical to understand the factors that will enable people to live longer and age in a healthy way.

To address the need for more accurate estimates of the relationship between smoking and healthy ageing and alcohol consumption and healthy ageing we conducted a systematic review and meta-analysis of longitudinal studies which examined the associations of these factors with the ageing process.

Methods

Search strategy and selection criteria

This systematic review has been written following the Preferred Reporting Items for a Systematic Review and Meta-Analysis (PRISMA) and the guidelines for Meta-Analyses Of Observational Studies in Epidemiology (MOOSE) to ensure accuracy and comprehensiveness.[17,18] (Appendix 1). A review protocol was prospectively registered in the international database of prospectively registered systematic reviews (PROSPERO) under registration number CRD42016038130 (Appendix 2). As part of a larger body of work considering modifiable lifestyle factors and healthy ageing, we originally planned to carry out a review focusing on the following: physical activity, smoking and alcohol consumption. The current systematic review specifically focuses on the findings related to smoking, alcohol consumption and healthy ageing outcomes since a sufficient amount of literature was identified on this topic alone. Our findings regarding healthy ageing and physical activity have been presented elsewhere.[19]

Two authors (C.D, C.K) searched MEDLINE (PubMed/PubMed Central interface), EMBASE (OVID interface), Psychinfo (OVID interface) and CENTRAL (Cochrane Central Register of Controlled Trials) from inception up to April 2016. Searching methodology

For peer review only - http://bmjopen.bmj.com/site/about/guidelines.xhtml

included any related term or synonym to healthy ageing and text word related to physical activity, smoking and alcohol consumption (Appendix 3). Other relevant systematic reviews and reference lists of the eligible studies were also searched. Finally, a second search was performed on 15th of March 2017 to include studies that were recently published.

Eligible studies had to meet the following criteria: i. being published in an electronic journal article; ii. constitute an original peer-reviewed longitudinal study; and iii. report any kind of longitudinal association between smoking and/or alcohol consumption and healthy ageing. Data on smoking and alcohol, through self-report and/or specific questionnaires, referred either to the current status and/or across the life span. The primary outcome of this review was health status measured by healthy ageing, and any other term related to it (e.g. successful ageing, active ageing, healthy survival etc.). Studies whose primary goal was the examination of a different determinant but included the aforementioned factors as covariates were also included. Due to the heterogeneity of the healthy ageing definition, studies reporting the latter as multiple outcomes or based solely on self-report were excluded. Studies that included cohorts of individuals who were institutionalised or hospitalised and animal studies were also excluded. No language restriction was applied.

In order to store the studies that were retrieved by the electronic search we used an EndNote (ENDNOTE X7, Thomson Reuters) library. After removal of duplicates, the two independent reviewers screened titles and abstracts of all potentially eligible papers. At the end of this procedure, any disagreement was solved by discussion. In case that an agreement could not be achieved, eligibility of the study was judged by discussion with a third senior researcher (A.M.P.). In case that full text could not be retrieved, the corresponding author of the paper was contacted via e-mail.

For peer review only - http://bmjopen.bmj.com/site/about/guidelines.xhtml

Data extraction and quality assessment

C.D. and C.K. independently extracted data from each study and a random sample of them was cross-checked by A.M.P. Setting/country of the study, data collection period, follow-up year, sample size, population, and baseline age information was recorded for each study. Definition and measurement of the healthy ageing outcome, of smoking and alcohol consumption were also recorded, as well as the odds ratios (OR) (or any other related statistic) and the 95% confidence intervals (CI). Reported statistics were extracted for the least and most adjusted models reported in the study.

The methodological quality of the included studies was assessed by using the Quality in Prognosis Studies (QUIPS) tool. QUIPS evaluates six potential components of bias: inclusion, attrition, prognostic factor measurement, confounders, outcome measurement, and analysis and reporting.[20] During the application of the QUIPS tool smoking and/or alcohol consumption were considered as the only prognostic factors and all other variables, used as explanatory variables of the model, were considered as confounders. Since we included only longitudinal studies, the existence of attrition was expected. In the scenario that the attrition rate was high, authors' explanations were required to evaluate the study as bearing low risk of bias. Finally, the reliability of statistical models was evaluated according to the data presented; for instance, the reliability of studies that included results only for the statistically significant factors was judged with caution.

Patient and public involvement

No patients were involved in this study. We used data from published papers only.

Data analysis

For peer review only - http://bmjopen.bmj.com/site/about/guidelines.xhtml

We performed meta-analyses in order to produce a pooled effect size estimate for the relationship between smoking and healthy ageing and alcohol consumption and healthy ageing. Separate meta-analyses were performed for the following categories:

Smoking: i) past and never smokers compared to current smokers; ii) former or never smokers compared to current smokers; iii) never smokers compared to former or current smokers.

Alcohol: For studies that provided alcohol quantities, we converted grams to drinks by assuming that 12 grams of alcohol are approximately one drink to have an alcohol consumption variable comparable among studies.[21] In studies that provided alcohol consumption in an interval we took the middle of the interval as the most likely value. We created the following categories: light (< 1 drink/day), moderate (1–2 drinks/day), and high (> 2–4 drinks/day) consumption that were compared to never drinkers. Studies that did not report specific alcohol quantity but characterised the participants as drinkers or non-drinkers were examined separately.

We produced pooled effect estimates of the aforementioned categories for the most adjusted models. Due to the expected heterogeneity, a random effects meta-analysis using the DerSimonian-Laird model was performed.[22] If a study reported different results for men and women, both results were included, except in cases where a result for the mixed population was also provided; the same rationale was applied to cases that reported statistics per different subgroups in the cohort. We also investigated via subgroup analyses, the effect of baseline age (i.e. studies with baseline mean age more than 65 years old and studies with baseline mean age less or equal to 65 years old) and follow-up time (i.e. studies with follow-up time more than 10 years and studies with follow-up time less or equal to 10 years) to the pooled effect estimates. Heterogeneity was assessed with I^2 statistic for each analysis,[23] publication bias[24] was assessed with Egger bias test.[25] Finally, a trim-and-fill adjusted

For peer review only - http://bmjopen.bmj.com/site/about/guidelines.xhtml

analysis was conducted to adjust for potential publication bias.[26] Analyses were performed in STATA 14 IC.

Results

The search identified 6,706 papers from the databases and 30 additional papers were obtained from other sources. After removing of duplicates and of papers that were conference papers, cross-sectional studies or animal studies, 73 papers were considered for full text-review. We excluded 42 papers based on specific reasons after full text-review and finally 28 studies are included in this report.[27-54] PRISMA flow diagram depicts the exact selection process **Figure 1**. Across the 28 eligible studies, there were 184,543 participants (almost 31% men), with sample size ranging from 456 to 68,153. Studies were published from 1989 to 2016 and half of them (14 out of 28) took place in USA. Studies were also conducted in England (four), in Australia (three), in China (two), in Canada (two), in Nigeria (one), in the Netherlands (one) and in Taiwan (one). Baseline age ranged from 14 to 109 years old and follow-up time from two years until death (>70 years old). Five studies examined men only and two studies examined women only; the majority of studies reported results for mixed populations (**Table 1**).

Healthy ageing, and any other term used as a synonym, was defined by including various domains of information in each study. These were grouped in the following categories: survival to a specific age or during follow-up, health status (either self-reported or measured by specific questionnaires), physical performance (including information regarding mobility, disabilities and/or difficulties in activities of daily living (ADL) and instrumental activities of daily living (iADL)), diseases (including chronic diseases and cancer), mental health and cognition status, subjective measurements of the participants (life satisfaction, happiness, and pain) and other (anthropometric measurements, personal assistance, social support) (**Figure 2**). Most of the studies (23 out of 28) included physical performance in their definition of

For peer review only - http://bmjopen.bmj.com/site/about/guidelines.xhtml

healthy ageing and more than half of them included information regarding diseases and mental health (18 and 16 out of 28 respectively). Survival to a specific age was also an area often found in the definition of healthy ageing, whereas health status and subjective measurements were not so often included. (Supplemental Table A1 presents the areas of information included in the definition of healthy ageing per study).

In **Table 2** the analytical results of this systematic review are presented. Relevant statistics per study are provided for every group of smoking and alcohol consumption variable and for the most and least adjusted models; confounders used for the final adjustment are also provided.

Quality Assessment

Of the 28 studies, one was evaluated as having high risk of bias, nine as moderate and 18 as having low. In aggregate, the quality of the included studies was above moderate. As it was expected, attrition and confounding were the issues that primarily contributed to moderate and high bias. Specifically, 18 out of 28 studies reported moderate or high risk of bias regarding the fact that the population lost to follow-up may be associated with key characteristics that could influence the observed relationship between outcome and factors. Similar results were also observed for the confounders' domain, where 18 out of 23 studies were characterised as having moderate risk of bias, meaning that important confounders may have not been appropriately accounted in the final model. (Supplemental Table A2).

Meta-analysis

Smoking and Healthy Ageing

From the 27 studies providing results for smoking we did a meta-analysis with 18 studies. We excluded studies that provided results using: i) healthy/successful years;[30,42] ii) Risk Ratio;[40] iii) coefficients from linear mixed models or generalised estimation equation models;[41,50] and

iv) cigarette-packs.[45,52,53] All studies included in the meta-analysis were of moderate and low risk of bias.

Never smokers compared to current smokers had more than double increased odds of ageing healthily (OR 2.36, 95%CI 2.03-2.75, I^2 43.30%, 7 studies) even after adjusting for publication bias with the trim-and-fill algorithm (OR 2.11, 95%CI 1.82-2.45, 10 studies). Never smokers had increased odds ratios when compared to former smokers as well (OR 1.32, 95%CI 1.23-1.41, I^2 32.80%, 5 studies); no change of the combined effect estimate was observed by the trim-and-fill method. Never smokers were more likely to experience healthy ageing than current or former smokers (OR 1.29, 95%CI 1.16-1.43, I^2 0.00%, 5 studies) and this finding marginally altered when we adjusted for publication bias (OR 1.27, 95%CI 1.14-1.40, 9 studies). Never or former smokers were more likely to age in a healthy way compared to current smokers (OR 1.72, 95%CI 1.20-2.47, I^2 87.20%, 6 studies) however, when we adjusted for publication bias this finding was no longer significant (OR 1.20, 95%CI 0.83-1.73, 9 studies).

There were also two studies that reported hazard ratios (HR);[47,48] the pooled HR of never smokers compared to current smokers was 1.55 (95%CI 1.18-2.02); while the pooled HR of former smokers compared to never smokers was 1.30 (95%CI 1.07-1.59). Egger test was not significant in any analysis that we performed (p-value>0.05), hence no evidence for publication bias was provided (**Table 3**). The synthesis of the results is provided in **Figure 3**.

Subgroup Analysis

Baseline age: The association of smoking with healthy ageing did not change direction or strength in the subgroup analyses. However, the beneficial association of non-smoking was higher in studies with baseline mean age less than 65 years old. The only association that differed was that in the analysis of former or never compared to current smokers. Studies

For peer review only - http://bmjopen.bmj.com/site/about/guidelines.xhtml

with relatively older participants showed a non-significant association (OR 1.34, 95%CI 0.65-2.76) whereas studies with relatively younger participants showed a statistically significant association (OR 1.90, 95%CI 1.51-2.40) (Supplemental Figure A1).

Follow-up time: We performed subgroup analyses for former or never versus current smokers and never versus former or current smokers; all other groups had follow-up time more than 10 years. In the group of former or never smokers compared to current smokers the association was not significant in studies with less than 10 years follow-up (OR 1.41, 95%CI 0.99-2.01). No considerable differences were observed in the group of never compared to former or current smoking with healthy ageing (Supplemental Figure A2).

Alcohol Consumption and Healthy Ageing

From the 21 studies that provided statistics for the association of alcohol consumption with healthy ageing we performed meta-analyses using nine studies. We excluded studies for the following reasons: i) the standardisation of alcohol consumption was not possible;[28,38,40,44,46,48,52-54] ii) they provided healthy years[30] or β coefficients.[41,50]

Drinkers compared to non-drinkers had increased odds of ageing healthily (OR 1.28, 95%CI 1.08-1.52, I^2 72.10%, 5 studies); the same beneficial association of alcohol was also observed when we pooled results among studies that reported findings of light consumption (<1 drinker per day) and moderate consumption (1-2 drinks per day) compared to non-drinkers: (OR 1.12, 95%CI 1.03-1.22, I^2 0.00%, 3 studies); (OR 1.35, 95%CI 0.93-1.97, I^2 71.40%, 4 studies). However, in the latter comparison (i.e. moderate consumption compared to non-drinkers) results were not statistically significant (p-value 0.112). We also pooled results of the studies reporting high (>2-4 drinks per day) consumption compared to non-drinkers; OR 1.25, (95%CI 1.09-1.44, I^2 0.00%, 3 studies). Results changed when we used the trim-and-fill algorithm only to the case of moderate to non-drinkers (OR 1.10, 95%CI 0.77-1.57, 6

For peer review only - http://bmjopen.bmj.com/site/about/guidelines.xhtml

studies). Egger test of bias was significant only in the analysis of light drinkers (p-value 0.043) (**Table 3**). The synthesis of the results is provided in **Figure 4**.

Subgroup Analysis

Baseline age: We could not perform subgroup analyses for the associations of alcohol consumption with healthy ageing as studies were homogeneous.

Follow-up time: We performed subgroup analysis for drinkers versus non-drinkers; all other groups were homogeneous (i.e. more than 10 years). The association of alcohol consumption with healthy ageing was significantly positive in studies with follow-up time more than 10 years (OR 1.41, 95%CI 1.36-1.48) whereas studies with follow-up time less than 10 years showed a non-significant beneficial association (OR 1.09, 95%CI 0.95-1.24) (Supplemental Figure A3).

Discussion

Based on our knowledge, this systematic review is the first to examine the association of smoking and alcohol consumption with healthy ageing, which included a meta-analysis to produce a pooled effect estimate and also examined eligible studies for publication bias. 23 studies out of 27 reported a positive association between never or former smoking and healthy ageing and four reported a non-significant relationship. With regards to alcohol consumption, results were mixed but a positive association of drinking with healthy ageing was reported in 12 out of 22 studies. Nine studies did not find an association and one study reported a negative one.

The current systematic review adds to the plethora of evidence that smoking is associated with worse health outcomes in older age. In fact, the only study that reported a positive association between current smoking and healthy ageing was that of Li et al. (2001)[40] where smokers exhibit increased risk ratios of healthy survival (RR 1.11, 95%CI 1.01-1.21);

For peer review only - http://bmjopen.bmj.com/site/about/guidelines.xhtml

however authors recommend to treat this finding with caution as the population attributable percentage (PARP) is very low after five years of follow-up (2.55%). In aggregate, being a non-smoker or a former smoker increases considerably the odds of ageing healthily. From our meta-analysis we found that never smokers have more than double the odds of experiencing healthy ageing (OR 2.36, 95%CI 2.03-2.75) compared to current smokers. Furthermore, never smokers are also more likely to age in a healthy way by more than 30% (OR 1.32, 95%CI 1.23-1.41) compared to former smokers. These findings indicate that smoking cessation is always beneficial and come in accordance with a 50 years study on doctors' mortality in relation with smoking. In this study, it was revealed that smoking cessation at age 60, 50, 40, or 30 years could increase life expectancy by 3, 6, 9, or 10 years, respectively.[55] Our findings also confirm previous research examining the beneficial effect of non-smoking on successful ageing.[56] In addition, we found that never/former smokers compared to current smokers have an increased likelihood of healthy ageing (OR 1.72, 95%CI 1.20-2.47), which becomes non-significant when we adjust for publication bias (OR 1.20, 95%CI 0.83-1.73). This comes in agreement with the finding of a study examining the transitions within smoking categories and successful ageing, where former compared to current smokers cannot predict successful ageing.[57]

Positive associations between drinking and healthy ageing refer to limited alcohol consumptions; for example 1-40 gr per day,[37] 1-14 drinks per week,[38] 1-14 units per week for women and 1-21 units per week for men[46]. The one study reporting a negative association is that of Willcox et al. (2006);[54] however, this finding should be interpreted with caution as drinkers with more than 3 drinks per day are compared to non-drinkers only and there is no comparison among light or moderate drinkers and non-drinkers. From our meta-analysis, we concluded that compared to never drinkers reasonable alcohol consumption is beneficial to healthy ageing; pooled OR for drinkers 1.28 (95%CI 1.08-1.52), light drinkers 1.12 (95%CI

For peer review only - http://bmjopen.bmj.com/site/about/guidelines.xhtml

1.03-1.22), moderate drinkers 1.35 (95%CI 0.93-1.97) and high drinkers 1.25 (95%CI 1.09-1.44). Nevertheless, associations are marginally statistically significant, while for the moderate category the pooled effect estimate is non-significant, so extra caution is needed before making a final conclusion.

Several studies of the associations of alcohol consumption with healthy ageing were excluded from our meta-analyses; among those there were seven studies that reported positive association between alcohol consumption and healthy ageing,[38,40,41,44,46,48,50] four studies reported non-significant association,[28,30,52,53] and one study reported a negative association.[54] As a consequence, we expect the positive effect of light to moderate alcohol drinking would be reinforced, if we included these studies in our meta-analyses. This finding comes in accordance with the fact that light to moderate alcohol consumption (≤1 drink per day) is also associated with a reduced risk of multiple cardiovascular outcomes,[11] better cognition and well-being,[58] and a reduced risk of substantial functional health decline.[59]

However, relating moderate alcohol consumption with health benefits should not come without question since there are many other issues to consider. Our results could be biased by confounders that have not been taken into account, such as health status or former and occasional drinkers to be counted as non-drinkers.[60] For instance, in the sub-group of drinkers the positive effect comes mainly from the study of LaCroix et al. (2016);[39] however, in that study former drinkers were also assumed as non-drinkers.

In addition, since typical drink sizes vary per country as well as the recommended daily or weekly amounts,[61] we cannot be sure that participants have been assigned to the correct drinking category. Our results should also be questioned regarding the fact that alcohol consumption was only assessed at baseline (exception to this is the study of Sun et al. (2011))[49]; during the baseline assessment some cohorts could have had middle-aged participants drinking up to four drinks per day and that could be considered a moderate

For peer review only - http://bmjopen.bmj.com/site/about/guidelines.xhtml

consumption. However, older people do not have the same tolerance to alcohol[62] and for them moderate consumption should consist of up to two drinks per day. Likewise, it should have been considered that older adults tend to consume less alcohol than middle-aged groups[63] and hence baseline assessments perhaps are not representative of alcohol consumption in an older age. Nevertheless, more research is required to establish or reject the beneficial effects of limited alcohol consumption. In an extended systematic review and meta-analysis of alcohol impact on mortality risk, the benefits of low-volume drinkers largely disappeared once studies were controlled for several design and methodological issues.[64] Mendelian randomisation studies, helping to examine causal relationships in observational studies by limiting confounding and reverse causation risk of bias,[65] may also contribute to elucidating if this beneficial effect of light alcohol consumption with healthy ageing is a true biological effect or not. For instance, a recent study of this type found no causal association between alcohol consumption and Alzheimer's disease.[66]

Strengths and Limitations

The fact that this review was independently double screened, taking into account previous systematic reviews in the field and the reference lists of included papers, allows a good amount of confidence that all relevant studies were included. Regarding the quality assessment of the studies, limited disagreement among the six different domains per study was reached between the two reviewers, who independently assessed them, concluding that the quality assessment tool was reliable and did not allow great amount of misjudgement. Attrition rate and not allowing important confounders to be included in the final models were important factors contributing to the quality of the considered studies. Hence future research should consider these domains more thoroughly.

However, the following limitations have to be taken into account. Smoking and alcohol consumption were not measured or/and defined in a consistent way among the different

For peer review only - http://bmjopen.bmj.com/site/about/guidelines.xhtml

cohorts; hence it was not possible to create a pooled effect size estimate across all eligible studies. In addition, healthy ageing definition was a source of heterogeneity since each study provided its own definition. Nevertheless, it is noteworthy that all studies assumed survival in the follow-up, even if the latter was not explicitly mentioned in the healthy ageing definition, and that many had definitions that included areas of physical performance, in agreement with the Depp and Jeste, (2006) review on successful ageing.[56] In addition, most of the studies used a categorical definition of smoking making impossible the assessment of abstinence time or pack-years on healthy ageing. Alcohol measurement and drinking categorisation were also another part of the underlying heterogeneity. Similar comments have been reported in other systematic reviews examining alcohol consumption.[64,67] Furthermore, self-reported questionnaires and the fact that each study was adjusted by using a different set of covariates, different follow-up time and attrition rate, and the different definition of healthy ageing increases the heterogeneity and the likelihood of bias. Especially for alcohol, research has shown that self-reported consumption is mostly underestimated.[68]

Due to the limited number of studies included in our meta-analyses we did not assess publication bias by funnel plots; instead we used Egger test and even though it was statistically non-significant in the majority of cases we applied the trim-and-fill method to examine the robustness of our findings. We used random effect meta-analysis to consider the heterogeneity of the studies, even though I^2 was not considerable in all analyses (**Table 3**). We did not assess heterogeneity with the Cochran's Q statistic due to the quite limited number of studies; performing meta-regression analysis and assessing the confounding effect of covariates (i.e age, gender, follow-up time) was not possible either. However, we did evaluate the robustness of our findings by also implementing the Paule and Mandel (PM) estimator to assess between study variability and estimate our pooled results, as simulations

For peer review only - http://bmjopen.bmj.com/site/about/guidelines.xhtml

have shown that the PM estimator is less biased and more efficient than other alternatives.[69] We did not observe any considerable fluctuations in our results (Supplemental Table A3).

We assessed the effect of different baseline age and follow-up time to the pooled estimates by performing subgroup analyses; pooled effect estimates were not severely affected in direction or in strength. The more positive effect of non-smoking to studies with relatively younger cohorts comes in accordance with other studies examining smoking and adverse health outcomes in old populations.[70,71] When studies incorporate an older sample this is biased in favour of people who smoke and survive compared to smokers who die (survival bias). Subgroup analyses showed that in studies with more than 65 years old participants or follow-up time less than 10 years, the beneficial association of former/never compared to current smokers with healthy ageing is not statistically significant; result which was also indicated by the trim-and-fill analysis. Follow-up subgroup analysis confirmed that the beneficial effect of drinking compared to non-drinkers comes from the study of LaCroix et al. (2016) emphasising that this result should be interpreted with caution.

From our review, it is evident that the majority of the studies have been implemented in high-income countries (24 out of 28 studies). Thus, in accordance with a previous study,[72] our findings revealed the limited research on ageing in low-and-middle income countries (LMIC), even though by 2050 80% of people aged 60 years and over will live there.[73] This is of high importance considering that alcohol use increases in LMICs[74] and that nowadays, 80% of the more than one billion smokers live there.[75] Estimations also indicate that by 2030 four out of five smoking-related deaths will occur there highlighting even more the heavy economic burden of smoking in these countries.[76]

Conclusions

In conclusion, smoking abstinence and smoking cessation are positively associated with healthy ageing. As it takes more than 20 years for most smoking-related diseases to develop,

For peer review only - http://bmjopen.bmj.com/site/about/guidelines.xhtml

the best practice would be to enforce smoking prevention policies, such as marketing bans and high taxation, and reduce smoking uptake among younger cohorts.[1] A positive relationship between limited alcohol consumption and healthy ageing could also be argued but more research is needed. From our research it becomes evident that study designs should be comparable to conclude with more confidence the generalisability of our findings. This could be achieved by adopting similar measurements of smoking and alcohol behaviour and by implementing a unanimous metric of healthy ageing. Finally, further research should focus on the examination of ageing in LMICs.

Supplementary Material

Appendix1.pdf (MOOSE checklist)

Appendix2.pdf (Prospero Protocol)

Appendix3. pdf (Searching Methodology)

Table A1. pdf (Healthy Ageing Definition)

Table A2. pdf (Quality Assessment)

Table A3. pdf (Sensitivity analysis of meta-analytic results)

Figure A1. pdf (Smoking and healthy ageing-age stratified)

Figure A2. pdf (Smoking and healthy ageing-follow-up time stratified)

Figure A3. pdf (Alcohol and healthy ageing-follow-up stratified)

Contributors

CD designed the study, carried out the literature review, data extraction, statistical analysis, data interpretation, article preparation, article review and correspondence. BS contributed to the data interpretation and article review. CK contributed to the literature review and data extraction. AK contributed to article preparation and article review. MP contributed to the study design, article preparation and article review. AMP contributed to the study design,

For peer review only - http://bmjopen.bmj.com/site/about/guidelines.xhtml

data interpretation, article preparation and article review. All authors contributed to the final report and approved the final version.

Acknowledgements

We thank Chih-Cheng Chang for his contribution to the translation of the non-English study. We thank ATHLOS (Ageing Trajectories of Health: Longitudinal Opportunities and Synergies) project, funded by the European Union's Horizon 2020 Research and Innovation Programme under grant agreement number 635316.

Funding

This project falls under the ATHLOS (Ageing Trajectories of Health: Longitudinal Opportunities and Synergies) project, funded by the European Union's Horizon 2020 Research and Innovation Programme under grant agreement number 635316. The sponsor had no participation in the study design, data extraction, data interpretation, or writing of this study.

A. M. P. was supported by the MRC MR/K021907/1.

A. K. was supported by a grant funded by the National Institute for Health Research (NIHR) Biomedical Research Centre at South London and Maudsley National Health Service (NHS) Foundation Trust and Kings' College London where most of this work was conducted. The views expressed are those of the authors and not necessarily those of NIHR, the NHS, or the UK Government Department of Health. A .K. is now formally based at the Liverpool School of Tropical Medicine.

Declaration of interests

All authors declare no conflicts of interest.

Data sharing statement

For peer review only - http://bmjopen.bmj.com/site/about/guidelines.xhtml

Statistical code is available upon request to the corresponding author; no other additional data are available.

References

1. Gibson G, Loddenkemper R, Sibille Y, et al. The European Lung White Book. 1st ed: European Respiratory Society 2013.
2. Peters R, Poulter R, Warner J, et al. Smoking, dementia and cognitive decline in the elderly, a systematic review. *BMC Geriatr* 2008;8:36. doi: 10.1186/1471-2318-8-36
3. Gellert C, Schottker B, Brenner H. Smoking and all-cause mortality in older people: systematic review and meta-analysis. *Arch Intern Med* 2012;172(11):837-44. doi: 10.1001/archinternmed.2012.1397
4. GHK. *A study on liability & the health costs of smoking DG SANCO (2008/C6/046) updated final report* http://ec.europa.eu/health//sites/health/files/tobacco/docs/tobacco_liability_final_en.pdf (accessed December 13, 2016).
5. Goodchild M, Nargis N, Tursan d'Espaignet E. Global economic cost of smoking-attributable diseases. *Tobacco Control* 2017 doi: 10.1136/tobaccocontrol-2016-053305
6. World Health Organization. *Global status report on alcohol and health 2014.* 2014. http://apps.who.int/iris/bitstream/10665/112736/1/9789240692763_eng.pdf.
7. Organization WH. Alcohol, Fact Sheet: World Health Organization.
8. Chapter 2 - Alcohol. Center for Substance Abuse Treatment Substance Abuse Among Older Adults Treatment Improvement Protocol (TIP) Series, No 26. Rockville (MD): Substance Abuse and Mental Health Services Administration (US) 1998. Available from: https://www.ncbi.nlm.nih.gov/books/NBK64419/.
9. The Royal College of Psychiatrists. *Alcohol and Older People* http://www.rcpsych.ac.uk/healthadvice/problemsdisorders/alcoholandolderpeople.aspx (accessed February 7, 2017).
10. Mukamal KJ, Kuller LH, Fitzpatrick AL, et al. Prospective study of alcohol consumption and risk of dementia in older adults. *Jama* 2003;289(11):1405-13.
11. Ronksley PE, Brien SE, Turner BJ, et al. Association of alcohol consumption with selected cardiovascular disease outcomes: a systematic review and meta-analysis. *BMJ* 2011;342 doi: 10.1136/bmj.d671
12. De Leon J, Rendon DM, Baca-Garcia E, et al. Association between smoking and alcohol use in the general population: stable and unstable odds ratios across two years in two different countries. *Alcohol Alcohol* 2007;42(3):252-7. doi: 10.1093/alcalc/agm029
13. Stotts AL, Schmitz JM, Grabowski J. Concurrent treatment for alcohol and tobacco dependence: are patients ready to quit both? *Drug Alcohol Depend* 2003;69(1):1-7.
14. Xu WH, Zhang XL, Gao YT, et al. Joint effect of cigarette smoking and alcohol consumption on mortality. *Prev Med* 2007;45(4):313-9. doi: 10.1016/j.ypmed.2007.05.015
15. Hart CL, Davey Smith G, Gruer L, et al. The combined effect of smoking tobacco and drinking alcohol on cause-specific mortality: a 30 year cohort study. *BMC Public Health* 2010;10:789. doi: 10.1186/1471-2458-10-789
16. United Nations. *World Population Ageing 2015.* 2015. http://www.un.org/en/development/desa/population/publications/pdf/ageing/WPA2015_Report.pdf.
17. Shamseer L, Moher D, Clarke M, et al. Preferred reporting items for systematic review and meta-analysis protocols (PRISMA-P) 2015: elaboration and explanation. *BMJ* 2015;349:g7647. doi: 10.1136/bmj.g7647

For peer review only - http://bmjopen.bmj.com/site/about/guidelines.xhtml

18. Stroup DF, Berlin JA, Morton SC, et al. Meta-analysis of observational studies in epidemiology: A proposal for reporting. *JAMA* 2000;283(15):2008-12. doi: 10.1001/jama.283.15.2008
19. Daskalopoulou C, Stubbs B, Kralj C, et al. Physical activity and healthy ageing: A systematic review and meta-analysis of longitudinal cohort studies. *Ageing Res Rev* 2017;38:6-17. doi: 10.1016/j.arr.2017.06.003
20. Hayden JA, van der Windt DA, Cartwright JL, et al. Assessing bias in studies of prognostic factors. *Ann Intern Med* 2013;158(4):280-6. doi: 10.7326/0003-4819-158-4-201302190-00009
21. Larsson SC, Wallin A, Wolk A, et al. Differing association of alcohol consumption with different stroke types: a systematic review and meta-analysis. *BMC Med* 2016;14(1):178. doi: 10.1186/s12916-016-0721-4
22. DerSimonian R, Laird N. Meta-analysis in clinical trials. *Control Clin Trials* 1986;7(3):177-88.
23. Higgins JP, Thompson SG, Deeks JJ, et al. Measuring inconsistency in meta-analyses. *Bmj* 2003;327(7414):557-60. doi: 10.1136/bmj.327.7414.557
24. Sterne JA, Egger M, Smith GD. Systematic reviews in health care: Investigating and dealing with publication and other biases in meta-analysis. *BMJ* 2001;323(7304):101-5.
25. Egger M, Smith GD, Schneider M, et al. Bias in meta-analysis detected by a simple, graphical test. *BMJ* 1997;315(7109):629-34. doi: 10.1136/bmj.315.7109.629
26. Duval S, Tweedie R. Trim and fill: A simple funnel-plot-based method of testing and adjusting for publication bias in meta-analysis. *Biometrics* 2000;56(2):455-63.
27. Andrews G, Clark M, Luszcz M. Successful Aging in the Australian Longitudinal Study of Aging: Applying the MacArthur Model Cross–Nationally. *Journal of Social Issues* 2002;58(4):749-65. doi: 10.1111/1540-4560.00288
28. Bell CL, Chen R, Masaki K, et al. Late-life factors associated with healthy aging in older men. *J Am Geriatr Soc* 2014;62(5):880-8. doi: 10.1111/jgs.12796
29. Britton A, Shipley M, Singh-Manoux A, et al. Successful aging: the contribution of early-life and midlife risk factors. *J Am Geriatr Soc* 2008;56(6):1098-105. doi: 10.1111/j.1532-5415.2008.01740.x
30. Burke GL, Arnold AM, Bild DE, et al. Factors associated with healthy aging: the cardiovascular health study. *J Am Geriatr Soc* 2001;49(3):254-62.
31. Ford AB, Haug MR, Stange KC, et al. Sustained personal autonomy: a measure of successful aging. *J Aging Health* 2000;12(4):470-89. doi: 10.1177/089826430001200402
32. Gu D, Zhang Z, Zeng Y. Access to healthcare services makes a difference in healthy longevity among older Chinese adults. *Soc Sci Med* 2009;68(2):210-9. doi: 10.1016/j.socscimed.2008.10.025
33. Guralnik JM, Kaplan GA. Predictors of healthy aging: prospective evidence from the Alameda County study. *Am J Public Health* 1989;79(6):703-8.
34. Gureje O, Oladeji BD, Abiona T, et al. Profile and determinants of successful aging in the Ibadan Study of Ageing. *J Am Geriatr Soc* 2014;62(5):836-42. doi: 10.1111/jgs.12802
35. Hamer M, Lavoie KL, Bacon SL. Taking up physical activity in later life and healthy ageing: the English longitudinal study of ageing. *Br J Sports Med* 2014;48(3):239-43. doi: 10.1136/bjsports-2013-092993
36. Hodge AM, English DR, Giles GG, et al. Social connectedness and predictors of successful ageing. *Maturitas* 2013;75(4):361-6. doi: 10.1016/j.maturitas.2013.05.002
37. Hodge AM, O'Dea K, English DR, et al. Dietary patterns as predictors of successful ageing. *J Nutr Health Aging* 2014;18(3):221-7. doi: 10.1007/s12603-013-0405-0
38. Kaplan MS, Huguet N, Orpana H, et al. Prevalence and Factors Associated With Thriving in Older Adulthood: A 10-Year Population-Based Study. *J Gerontol A Biol Sci Med Sci* 2008;63(10):1097-104.
39. LaCroix AZ, Rillamas-Sun E, Woods NF, et al. Aging Well Among Women Veterans Compared With Non-Veterans in the Women's Health Initiative. *Gerontologist* 2016;56 Suppl 1:S14-26. doi: 10.1093/geront/gnv124

For peer review only - http://bmjopen.bmj.com/site/about/guidelines.xhtml

40. Li C, Zhang M, He Y, et al. Impact of health behavior on successful aging: A 5-year follow-up study among community elderly. *Chinese Mental Health Journal* 2001(15):324-26 (in Chinese).
41. Liu LF, Su PF. What factors influence healthy aging? A person-centered approach among older adults in Taiwan. *Geriatr Gerontol Int* 2017;17(5):697-707. doi: 10.1111/ggi.12774
42. Newman AB, Arnold AM, Naydeck BL, et al. "Successful aging": effect of subclinical cardiovascular disease. *Arch Intern Med* 2003;163(19):2315-22. doi: 10.1001/archinte.163.19.2315
43. Newson RS, Witteman JC, Franco OH, et al. Predicting survival and morbidity-free survival to very old age. *Age (Dordr)* 2010;32(4):521-34. doi: 10.1007/s11357-010-9154-8
44. Pruchno RA, Wilson-Genderson M. A Longitudinal Examination of the Effects of Early Influences and Midlife Characteristics on Successful Aging. *J Gerontol B Psychol Sci Soc Sci* 2015;70(6):850-9. doi: 10.1093/geronb/gbu046
45. Reed DM, Foley DJ, White LR, et al. Predictors of healthy aging in men with high life expectancies. *Am J Public Health* 1998;88(10):1463-8.
46. Sabia S, Singh-Manoux A, Hagger-Johnson G, et al. Influence of individual and combined healthy behaviours on successful aging. *CMAJ* 2012;184(18):1985-92. doi: 10.1503/cmaj.121080
47. Sarnak MJ, Katz R, Fried LF, et al. Cystatin C and aging success. *Arch Intern Med* 2008;168(2):147-53. doi: 10.1001/archinternmed.2007.40
48. Shields M, Martel L. Healthy living among seniors. *Health Rep* 2006;16 Suppl:7-20.
49. Sun Q, Townsend MK, Okereke OI, et al. Alcohol Consumption at Midlife and Successful Ageing in Women: A Prospective Cohort Analysis in the Nurses' Health Study. *PLOS Medicine* 2011;8(9):e1001090. doi: 10.1371/journal.pmed.1001090
50. Tampubolon G. Trajectories of the healthy ageing phenotype among middle-aged and older Britons, 2004–2013. *Maturitas* 2016;88:9-15. doi: 10.1016/j.maturitas.2016.03.002
51. Terry DF, Pencina MJ, Vasan RS, et al. Cardiovascular risk factors predictive for survival and morbidity-free survival in the oldest-old Framingham Heart Study participants. *J Am Geriatr Soc* 2005;53(11):1944-50. doi: 10.1111/j.1532-5415.2005.00465.x
52. Vaillant GE, Mukamal K. Successful aging. *Am J Psychiatry* 2001;158(6):839-47. doi: 10.1176/appi.ajp.158.6.839
53. Vaillant GE, Western RJ. Healthy aging among inner-city men. *International psychogeriatrics* 2001;13(4):425-37.
54. Willcox BJ, He Q, Chen R, et al. Midlife risk factors and healthy survival in men. *Jama* 2006;296(19):2343-50. doi: 10.1001/jama.296.19.2343
55. Doll R, Peto R, Boreham J, et al. Mortality in relation to smoking: 50 years' observations on male British doctors. *BMJ* 2004;328(7455):1519. doi: 10.1136/bmj.38142.554479.AE
56. Depp CA, Jeste DV. Definitions and predictors of successful aging: a comprehensive review of larger quantitative studies. *Am J Geriatr Psychiatry* 2006;14(1):6-20. doi: 10.1097/01.JGP.0000192501.03069.bc
57. Pruchno R, Hahn S, Wilson-Genderson M. Cigarette smokers, never-smokers, and transitions: implications for successful aging. *Int J Aging Hum Dev* 2012;74(3):193-209. doi: 10.2190/AG.74.3.b
58. Lang I, Wallace RB, Huppert FA, et al. Moderate alcohol consumption in older adults is associated with better cognition and well-being than abstinence. *Age Ageing* 2007;36(3):256-61. doi: 10.1093/ageing/afm001
59. Chen LY, Hardy CL. Alcohol consumption and health status in older adults: a longitudinal analysis. *J Aging Health* 2009;21(6):824-47. doi: 10.1177/0898264309340688
60. Stockwell T, Greer A, Fillmore K, et al. How good is the science? *Bmj* 2012;344:e2276; author reply e94. doi: 10.1136/bmj.e2276.
61. International Alliance for Responsible Drinking. *Drinking Guidelines - Data by Country - Worldwide* 2016. http://www.iard.org/policy-tables/drinking-guidelines-general-population.

For peer review only - http://bmjopen.bmj.com/site/about/guidelines.xhtml

62. Institute of Alcohol Studies. *Older people and alcohol Factsheet* http://www.ias.org.uk/uploads/pdf/Factsheets/Alcohol%20and%20older%20people%20FS%20May%202013.pdf (accessed December 14, 2016).
63. NHS Health. *Alcohol and Ageing. Is alcohol a major threat to healthy ageing for the baby boomers?* http://lx.iriss.org.uk/sites/default/files/resources/Alcohol%20and%20ageing.pdf (accessed December 15, 2016).
64. Stockwell T, Zhao J, Panwar S, et al. Do "Moderate" Drinkers Have Reduced Mortality Risk? A Systematic Review and Meta-Analysis of Alcohol Consumption and All-Cause Mortality. *J Stud Alcohol Drugs* 2016;77(2):185-98. doi: 10.15288/jsad.2016.77.185
65. Smith GD, Ebrahim S. Mendelian randomization: prospects, potentials, and limitations. *International Journal of Epidemiology* 2004;33(1):30-42. doi: 10.1093/ije/dyh132
66. Andrews SJ, Anstey KJ. Association between alcohol consumption and Alzheimer's disease: A Mendelian Randomization Study. *bioRxiv* 2017 doi: 10.1101/190165
67. Reid MC, Boutros NN, O'Connor PG, et al. The health-related effects of alcohol use in older persons: a systematic review. *Subst Abus* 2002;23(3):149-64. doi: 10.1080/08897070209511485
68. Stockwell T, Zhao J, Macdonald S. Who under-reports their alcohol consumption in telephone surveys and by how much? An application of the 'yesterday method' in a national Canadian substance use survey. *Addiction* 2014;109(10):1657-66. doi: 10.1111/add.12609
69. Veroniki AA, Jackson D, Viechtbauer W, et al. Methods to estimate the between-study variance and its uncertainty in meta-analysis. *Research synthesis methods* 2016;7(1):55-79. doi: 10.1002/jrsm.1164
70. Chang C-CH, Zhao Y, Lee C-W, et al. Smoking, death, and Alzheimer's disease: A case of competing risks. *Alzheimer disease and associated disorders* 2012;26(4):300-06. doi: 10.1097/WAD.0b013e3182420b6e
71. Hernan MA, Alonso A, Logroscino G. Cigarette smoking and dementia: potential selection bias in the elderly. *Epidemiology (Cambridge, Mass)* 2008;19(3):448-50. doi: 10.1097/EDE.0b013e31816bbe14
72. Chatterji S, Byles J, Cutler D, et al. Health, functioning, and disability in older adults—present status and future implications. *The Lancet* 2015;385(9967):563-75. doi: 10.1016/s0140-6736(14)61462-8
73. WHO.World Health Organization. *Ageing and health Factsheet* http://www.who.int/mediacentre/factsheets/fs404/en/ (accessed November 8, 2016).
74. Connor JP, Hall W. Alcohol burden in low-income and middle-income countries. *The Lancet* 2015;386(10007):1922-24. doi: 10.1016/S0140-6736(15)00236-6
75. World Health Organization. *Tobacco Factsheet* http://www.who.int/mediacentre/factsheets/fs339/en (accessed December 9, 2016).
76. World Health Organization. *WHO report on the global tobacco epidemic, 2008 : the MPOWER package.* 2008. http://apps.who.int/iris/bitstream/10665/43818/1/9789241596282_eng.pdf.

For peer review only - http://bmjopen.bmj.com/site/about/guidelines.xhtml

Table 1: Baseline characteristics of the eligible studies

Studies/Authors	Country	Panel	Data collection period	Follow-up*	Sample Size	Gender	Baseline Age
Andrews et al., 2002[27]	Australia	Australian Longitudinal Study of Aging (ALSA)	1992	8	1,403	55% men	> 70
Bell et al., 2014[28]	USA	Honolulu Heart Program (HHP)	1991 - 1993	up to 21 years	1,292	100% men	71-82
Britton et al., 2008[29]	England	Whitehall II study	1985 - 1988	17	5,823	71% men	35 - 55
Burke et al., 2001[30]	USA	Cardiovascular Health Study (CHS)	1989 - 1990, 1992 - 1993	6.5 and 3.5	3,342	39% men	> 65
Ford et al., 2000[31]	USA	N/A	1993	2	602	33% men	> 70
Gu et al., 2009[32]	China	Chinese Longitudinal Healthy Longevity Survey (CLHLS)	2002	3	15,972	45% men	65 - 109
Guralnik & Kaplan, 1989[33]	USA	Alameda County Study	1965	19	841	N/A	46 - 70
Gureje et al., 2014[34]	Nigeria	Ibadan Study of Ageing (ISA)	Aug 2003 - Nov 2004	5.3	930	61% men	> 65
Hamer et al., 2013[35]	England	English Longitudinal Study of Ageing (ELSA)	2002 - 2003	8	3,454	42% men	63.7 (M)
Hodge, English et al., 2013[36]	Australia	Melbourne Collaborative Cohort Study	1990 - 1994	11.7	5,512	37% men	63 (M)
Hodge, O'Dea et al., 2014[37]	Australia	Melbourne Collaborative Cohort Study	1990 - 1994	11.1 (WM)	6,309	39% men	64.1 (WM)
Kaplan et al., 2008[38]	Canada	Canadian National Population Health Survey (NPHS)	1994 - 1995	10	2,432	44% men	65 - 85
LaCroix et al., 2016[39]	USA	Women's Health Initiative (WHI)	1993 - 1998	16	68,153	100% women	68.9 (WM)
Li et al., 2001[40]	China	Shanghai Mental Health Center	1987	5	3,024	43% men	67.3 (M)
Liu & Su, 2016[41]	Taiwan	Taiwan Longitudinal Study on Aging (TLSA)	1993	14	3,155	56% men	71.7 (M)

For peer review only

For peer review only - http://bmjopen.bmj.com/site/about/guidelines.xhtml

Newman et al., 2003[42]	USA	Cardiovascular Health Study (CHS)	1989 - 1990, 1992 - 1993	8	2,932	39% men	71.9 (WM)
Newson et al., 2010[43]	Netherlands	Rotterdam Study	1990 - 1993	7.9	2,008	34% men	75.8 (M)
Pruchno & Wilson-Genderson, 2015[44]	USA	ORANJ BOWL: Ongoing Research on Aging in New Jersey: Bettering Opportunities for Wellness in Life	2006 -2008	4	2,614	37% men	60.5 (WM)
Reed et al., 1998[45]	USA	Honolulu Heart Program (HHP)	1965 - 1968	28	8,006	100% men	45 - 68
Sabia et al., 2012[46]	England	Whitehall II study	1991 - 1994	>16.3(Med)	5,100	70% men	51.3 (M)
Sarnak et al., 2008[47]	USA	Cardiovascular Health Study (CHS)	1989-1990 and 1992-1993 (African-American cohort)	4.3	2,140	38% men	74
Shields & Martel, 2006[48]	Canada	National Population Health Survey (NPHS)	1994-1995	8	1,309	N/A	> 65
Sun et al., 2011[49]	USA	Nurses' Health Study (NHS)	1980-1986	20 and 16	13,894	100% women	58 (Med)
Tampubolon, 2016[50]	England	English Longitudinal Study of Ageing (ELSA)	2004	9	14,765	46% men	50 - 89
Terry et al., 2005[51]	USA	Framingham Heart Study (FHS)	1948-1971	45	2,531	44% men	40 - 50
Vaillant & Mukamal, 2001[52]	USA	Study of Adult Development at Harvard University	circa 1940	until 60 or death	724	100% men	born mainly in the 1920s
Vaillant & Western, 2001[53]	USA	Study of Adult Development	1940 - 1945	60 y. (until 70 years or death)	456	100% men	14
Willcox et al., 2006[54]	USA	Honolulu Heart Program/Honolulu Asia Aging Study (HHP/HAAS)	1965-1968	40	5,820	100% men	54 (WM)

*: mean years, unless otherwise specified; M:mean; WM: Weighted Mean; Med: Median

For peer review only - http://bmjopen.bmj.com/site/about/guidelines.xhtml

Table 2: Aggregated Results of Smoking and Alcohol Consumption to Healthy Ageing

Authors	Smoking Variable	Statistics and 95%CI	Alcohol Variable	Statistics and 95%CI	Confounders
Andrews et al., 2002[27]	Past smoker (Yes or No)	no statistically significant (N/A)	-	-	Sociodemographic, Economic
Bell et al., 2014[28]	Past, Current, Never	Unhealthy vs Healthy Survival; OR, 95%CI<br>Past: 1.03, (0.78-1.35)<br>Current: 1.99, (1.06-3.75)<br>Never: Ref	Never (0 ounces/month);<br>Moderate to Heavy (>15 ounces/month, >1 drink per day);<br>Mild (>0–15 ounces/month, ≤1 drink per day)	Unhealthy vs Healthy Survival; OR, 95%CI<br>Never: 1.10, (0.81-1.50)<br>Moderate to Heavy: 1.04, (0.73-1.47)<br>Mild: Ref	Sociodemographic
Britton et al., 2008[29]	Never, Ex-smoker, Current	OR, 95%CI, Men:<br>Never: 2.7, (1.8-4.1), Not SEP adj: 3.2, (2.1-4.7)<br>Ex-smoker: 2.5, (1.6-3.7), Not SEP adj: 2.6, (1.8-4.0)<br>Current smoker: Ref<br>Women:<br>Never: 2.2, (1.3-3.7), Not SEP adj: 2.4, (1.5-3.9)<br>Ex-smoker: 2.2, (1.3-3.7), Not SEP adj: 2.6, (1.5-4.5)<br>Current smoker: Ref	Alcohol consumption in the previous week (1 Unit = 8 g ethanol).<br>0 unit/week, 1-14 units/week, 15 units/week	OR, 95%CI, Men:<br>0: 1.2, (0.9-1.8), Not SEP adj: 1.0, (0.7-1.4)<br>1-14: 1.0, (0.8-1.2), Not SEP adj: 1.0, (0.8-1.2)<br>15: Ref<br>Women<br>0: 0.5, (0.3-0.9), Not SEP adj: 0.3, (0.2-0.5)<br>1-14: 1.0, (0.7-1.5), Not SEP adj: 0.8, (0.5-1.1)<br>15: Ref	Sociodemographic, Economic, Model Characteristics

For peer review only - http://bmjopen.bmj.com/site/about/guidelines.xhtml

Burke et al., 2001[30]	Former, Current, Never	Proportion of HY, 95%CI:<br>Model with Behavioural Factors only<br>Former: 0.75, N/A<br>Current: 0.56, N/A<br>Never: Ref<br>Model with Behavioural Factors & Subclinical Disease Factors<br>Former: 0.78, (0.69, 0.88)<br>Current: 0.66, (0.56, 0.78)<br>Never: Ref	Wine Drink: Yes, No	Proportion of HY, 95%CI:<br>Model with Behavioural Factors only<br>Wine Drink: 1.11, (0.99, 1.24)<br>Model with Behavioural Factors & Subclinical Disease Factors<br>Wine Drink: 1.04, (0.93, 1.17)	Sociodemographic, Economic, Health Behaviour, Diseases and Physical Measurements
Ford et al., 2000[31]	Current Smoker: Yes, No	OR, 95%CI:<br>No: 2.14, (1.02-4.48)<br>Yes: Ref	Current drinker (wine, beer, or liquor): Yes, No	OR, 95%CI:<br>No: 1.00, (0.59-1.71)	Sociodemographic, Economic, Health Behaviour, Diseases and Physical Measurements, Attitude and Social Environment
Gu et al., 2009[32]	Current Smoker: Yes, No	OR, 95% CI of access to healthcare at present & in childhood on healthy survival<br>Model I: 1.05, (0.91-1.21)<br>Model II: 1.00, (0.86-1.16)<br>Model III: 1.00, (0.86-1.16)<br>No: Ref	Current Drinker: Yes, No	OR, 95% CI of access to healthcare at present & in childhood on healthy survival<br>Model I: 1.21, (1.05-1.39)<br>Model II: 1.11, (0.96-1.28)<br>Model III: 1.10, (0.96-1.27)<br>No: Ref	Model I: Sociodemographic, Economic<br>Model II: Sociodemographic, Economic, Attitude and Social Environment<br>Model III: Sociodemographic, Economic, Attitude and Social Environment, Model Characteristics

For peer review only

For peer review only - http://bmjopen.bmj.com/site/about/guidelines.xhtml

Guralnik & Kaplan, 1989[33]	Past-Never, Current	OR, 95%CI<br>High function vs all others (Dead or Lower/Moderate)<br>Health Practices Model: Past-Never: 2.8, (1.6-4.8), Current: Ref<br>Combined Model: Past-Never: 3.0, (1.8-5.1), Current: Ref<br><br>High function vs Deceased<br>Past-Never: 6.1, (3.3-8.3), Current: Ref<br>High function vs Low/Moderate<br>Past-Never: 2.2, (1.3, 3.8), Current: Ref	None, 1-60, >60 (drinks/month)	OR, 95%CI<br>High function vs all others (Dead or Lower/Moderate)<br>Health Practices Model: 1-60 vs None: 2.4, (1.2-4.8)<br>1-60 vs >60: 1.7, (0.6-5.0)<br>Combined Model: 1-60 vs None: 2.1, (1.1-4.1), 1-60 vs >60: 1.7, (0.6-5.1)<br><br>High function vs Deceased<br>1-60 vs None: 3.1, (1.5-6.5), 1-60 vs >60: 2.5, (0.7-8.2)<br>High function vs Low/Moderate<br>1-60 vs None: 2.2, (1.1-4.4)<br>1-60 vs >60: 1.3, (0.4-4.0)	Health Practices Model: Health Behaviour, Physical Measurement<br>Combined Model: Sociodemographic, Economic, Diseases, Physical Measurement, Health Behaviour
Gureje et al., 2014[34]	Ever smoking: Yes, No	OR, 95%CI<br>Total: No: 1.6, (0.71-3.46), Yes: Ref<br>Male: No: 4.7, (1.61-14.52), Yes: Ref<br>Female: No: 0.4, (0.22, 1.13), Yes: Ref	Ever having alcohol: Yes, No	OR, 95%CI<br>Total: No: 1.0, (0.51, 2.10), Yes: Ref<br>Male: No: 0.5, (0.10, 2.73), Yes: Ref<br>Female: No: 0.3, (0.47, 2.18), Yes: Ref	Sociodemographic, Economic, Health Behaviour, Diseases and Physical Measurements, Attitude and Social Environment
Hamer et al., 2013[35]	Current, Ex/Non-smoker	OR, 95%CI<br>Current: 0.66, (0.48-0.89)<br>Ex/Non-smokers: Ref	Daily, at least Weekly, Rarely, Never	-	Sociodemographic, Economic, Health Behaviour

For peer review only - http://bmjopen.bmj.com/site/about/guidelines.xhtml

Hodge, English et al., 2013[36]	Former, Current, Never	OR, 95% CI<br>Former: 0.72, (0.61, 0.84)<br>Current: 0.45, (0.32, 0.65)<br>Never: Ref	Alcohol intake (g/day)	OR, 95%CI<br>1–20: 1.06, (0.90-1.24)<br>21–40: 1.16, (0.93-1.46)<br>41–60: 1.02, (0.72-1.45)<br>60+: 1.25, (0.79-1.97)<br>None: Ref	Sociodemographic, Economic, Health Behaviour, Diseases and Physical Measurements, Attitude and Social Environment
Hodge, O'Dea et al., 2014[37]	Former, Current, Never	OR, 95%CI<br>Model without BMI and WHR<br>Former: 0.67, (0.59-0.77)<br>Current: 0.39, (0.30-0.50)<br>Never: Ref<br><br>Model with BMI and WHR<br>Former: 0.69, (0.60-0.79)<br>Current: 0.38, (0.29-0.48)<br>Never: Ref	Alcohol intake (g/day)	OR, 95%CI<br>Model without BMI and WHR<br>1-20: 1.19, (1.03-1.36)<br>21-40: 1.37, (1.11-1.67)<br>41-60: 1.27, (0.94-1.74)<br>60+: 1.17, (0.78-1.76)<br>None: Ref<br><br>Model with BMI and WHR<br>1-20: 1.17, (1.02-1.34)<br>21-40: 1.33, (1.09-1.63)<br>41-60: 1.29, (0.95-1.76)<br>60: 1.20, (0.80-1.80)<br>None: Ref	Sociodemographic, Economic, Health Behaviour, Diseases and Physical Measurements, Model Characteristics
Kaplan et al., 2008[38]	Smoker, Never smoker	OR, 95%CI<br>Thrivers vs NonThrivers: Smoker: 1.89, (1.10-3.23), Never smoker: Ref<br>Thrivers vs Deceased: Smoker: 4.35, (2.44-7.69), Never smoker: Ref	Moderate (1-14 drinks/week), None or Heavy	OR, 95%CI<br>Thrivers vs NonThrivers, Moderate: 1.78, (1.07-2.95), None-Heavy: Ref<br>Thrivers vs Deceased, Moderate: 2.22, (1.25-3.95), None-Heavy: Ref	Sociodemographic, Economic, Attitude and Social Environment, Health Behaviour, Diseases and Physical Measurements

For peer review only

LaCroix et al., 2016[39]	Current, Past, Never	OR, 95%<br>Crude Model, Veterans<br>Current: 0.35, (0.22-0.57), Past: 0.85, (0.71-1.02), Never: Ref<br>Adjusted Model, Veterans<br>Current: 0.31, (0.19-0.52), Past: 0.80, (0.66-0.97), Never: Ref<br>Crude Model, Non-Veterans<br>Current: 0.49, (0.45-0.54), Past: 0.83, (0.80-0.86), Never: Ref<br>Adjusted Model, Non-Veterans<br>Current: 0.50, (0.46-0.55), Past: 0.77, (0.74-0.80), Never: Ref<br><br>Model for Veterans Only:<br>Crude: Current: 0.35, (0.22-0.57), Past: 0.85, (0.71-1.02), Never: Ref<br>Adjusted: Current: 0.31, (0.18-0.52), Past: 0.79, (0.65-0.95), Never: Ref	Drinker, Non-Drinker	OR, 95%<br>Crude Model, Veterans<br>Non-drinker: 0.59, (0.48-0.73), Drinker: Ref<br>Adj. Model, Veterans<br>Non-drinker: 0.64, (0.51-0.81), Drinker: Ref<br>Crude Model, Non-Veterans<br>Non-drinker: 0.67, (0.64-0.69), Drinker: Ref<br>Adj. Model, Non-Veterans<br>Non-drinker: 0.71, (0.68-0.74), Drinker: Ref<br><br>Model for Veterans Only:<br>Crude: Non-drinker: 0.59, (0.48-0.73), Drinker: Ref<br>Adj.: Non-drinker: 0.62, (0.49-0.78), Drinker: Ref	Crude: Age<br>Adj.: Sociodemographic, Economic, Model Characteristics, Health Behaviour, Diseases and Physical Measurements
Li et al., 2001[40]	Current, Former, Never	RR, 95%CI<br>Current: 1.11, (1.01-1.21)<br>Former: 0.87, (0.74-1.03)<br>Never: 0.96, (0.88-1.05)	Current, Former, Non-Drinker	RR, 95%CI<br>Current: 1.16, (1.07-1.27)<br>Former: 0.87, (0.68-1.12)<br>Never: 0.89, (0.81-0.96)	Sociodemographic, Health Behaviour
Liu & Su, 2016[41]	Smoking (Yes/ No)	β= 0.042, SE=0.03, p-value: 0.163<br>No: Ref	Alcohol (Yes/ No)	β=0.201, SE: 0.03, p-value < 0.001<br>No: Ref	Sociodemographic, Health Behaviour, Attitude & Social Environment

For peer review only - http://bmjopen.bmj.com/site/about/guidelines.xhtml

Newman et al., 2003[42]	Self-Reported Pack-years: None =<10 >10-20 >20-40 >40	Proportion of SY, 95% CI Current Smoker: 0.77, (0.65-0.90) (multiplication factor) =<10 : 0.84 (0.73-0.98) >10-20 : 0.90 (0.76-1.07) >20-40 : 0.99 (0.85-1.15) >40 : 0.82 (0.71-0.95) None: Ref	-	-	Sociodemographic, Economic, Health Behaviour, Diseases and Physical Measurements
Newson et al., 2010[43]	Never, Former or Current	Survival Morbidity free vs non-survival, OR, 95% CI: Age and sex-adjusted model: 1.38, (1.07, 1.77) Full model: 1.26, (0.97, 1.65)	average consumption(gr)/day	not statistically significant	Sociodemographic, Economic, Health Behaviour, Diseases and Physical Measurements, Attitude & Social Environment
Pruchno & Wilson-Genderson, 2015[44]	Current Smoker (Yes, No)	Successful: Ref Unsuccessful: b=0.55, SD=0.26, 95% CI=(1.03, 2.93), exp(b)=1.74 Subjective only: b=0.32, SD=0.25, 95% CI=(0.83, 2.27), exp(b)=1.38 Objective only: b=0.24, SD=0.23, 95% CI=(0.81, 1.99), exp(b)=1.27	Days per week where have at least one drink of alcohol (0-7)	Successful: Ref Unsuccessful: b=-0.09, SD=0.08, 95% CI=(0.83, 1.00), exp(b)=0.91 Subjective only: b=-0.02, SD=0.04, 95% CI=(0.90, 1.06), exp(b)=0.98 Objective only: b=0.01, SD=0.03, 95% CI=(0.94, 1.08), exp(b)=1.01	Sociodemographic, Economic, Health Behaviour, Attitude & Social Environment
Reed et al., 1998[45]	Cigarette pack-years = (usual number of cigarettes/day) * (number of years)	OR, 95% CI Healthy vs Illness With Impairment: 0.52, (0.39-0.70) Healthy vs Illness Without Impairment: 0.46, (0.33-0.64) Healthy vs Impairment Without Illness: - Survival Ratio, 95%CI: 0.72, (0.65-0.79)	monthly intake of beer, wine, and liquor (ml of ethanol/day).	-	Sociodemographic, Health Behaviour, Diseases and Physical Measurement, Model Characteristics
Sabia et al., 2012[46]	Current or Former smoker, Never	OR, 95% CI Never Smoker: 1.29, (1.11, 1.49) Former or Current Smoker: Ref	Abstinence: no alcohol in the last week Moderate: 1–14 units/wk women; 1–21 units/wk men	OR, 95% CI Moderate : 1.31, (1.12, 1.53) No Moderate: Ref	Sociodemographic, Economic, Health Behaviour

For peer review only - http://bmjopen.bmj.com/site/about/guidelines.xhtml

			Heavy: ≥15 units/wk women; ≥21 units/wk men.		
Sarnak et al., 2008[47]	Current, Former, Never	HR, 95% CI<br>Current: 1.73, (0.79, 1.80)<br>Former: 1.21, (0.92, 1.58)<br>Never: Ref	-	-	Sociodemographic, Diseases and Physical Measurements
Shields & Martel, 2006[48]	Current daily smokers, had quit daily smoking within the past 15 years, had quit at least 15 years ago or had never smoked every day	HR, 95% CI<br>Current: 0.7, (0.5, 1.0)<br>Quit during past 15 yrs: 0.7, (0.5, 0.9)<br>Never smoked/ Quit for 15+ years: Ref	Weekly/ Occasional drinker, Non-drinker	HR, 95% CI<br>Weekly/ Occasional drinker: 1.4, (1.1, 1.8)<br>Non-drinker: Ref	Sociodemographic, Economic, Health Behaviour, Attitude & Social Environment
Sun et al., 2011[49]	-	-	Alcohol Intake Levels (g/d)<br>i. 0<br>ii. ≤5.0<br>iii. 5.1–15.0<br>iv. 15.1–30.0<br>v. 30.1–45.0	Fully adj. OR, 95% CI<br>i. 0 : Ref<br>ii. ≤5.0 : 1.11 (0.96–1.29)<br>iii. 5.1–15.0 : 1.19 (1.01–1.40)<br>iv. 15.1–30.0: 1.28 (1.03–1.58)<br>v. 30.1–45.0 : 1.24 (0.87–1.76)<br><br>Age adj. OR, 95% CI<br>i. 0 : Ref<br>ii. ≤5.0 : 1.12 (0.97–1.29)<br>iii. 5.1–15.0 : 1.22 (1.05–1.43)<br>iv. 15.1–30.0: 1.26 (1.03–1.53)<br>v. 30.1–45.0 : 1.05 (0.75–1.46)	Sociodemographic, Economic, Health Behaviour, Diseases and Physical Measurements

For peer review only

For peer review only - http://bmjopen.bmj.com/site/about/guidelines.xhtml

Tampubolon, 2016[50]	Current, No current	Baseline Model, Annual Rate of Phenotypic Decline: b: -0.679, 95%CI: (-0.852, -0.507)<br>Gender Interaction Model, Annual Rate of Phenotypic Decline: b: -0.685, 95%CI: (−0.858,−0.513)	Drink daily<br>Less than daily: Ref	Baseline Model, Annual Rate of Phenotypic Decline: b=0.414, 95%CI=(0.309, 0.519)<br>Gender Interaction Model, Annual Rate of Phenotypic Decline: b=0.412, 95%CI=(0.307, 0.517)	Baseline Model: Sociodemographic, Economic, Health Behaviour, Diseases and Physical Measurements
Terry et al., 2005[51]	Present (smoked within the year before any baseline examination), Absent	OR, 95%CI: 0.51, (0.41, 0.63)	-	-	Sociodemographic, Economic, Health Behaviour, Diseases and Physical Measurement
Vaillant & Mukamal, 2001[52]	Smoking < 30 pack-years (from age 15-50)<br>Smoking ≥ 30 pack-years (from age 15-50)	OR, 95%CI<br>Happy-Well Men vs Sad-Sick or Prematurely Dead<br>Smoking <30 pack-years: College Men at Age 75–80, 4.81, (0.84, 27.7)<br>Smoking <30 pack-years: Core-City Men at Age 65–70, 4.56, (2.29, 9.11)<br>Smoking in ≥30 pack-years: Ref	Alcohol Abuse Absent (Yes, No)	OR, 95%CI<br>Happy-Well Men vs Sad-Sick or Prematurely Dead<br>No alcohol abuse: College Men at Age 75–80, -<br>No alcohol abuse: Core-City Men at Age 65–70, 1.11, (0.53–2.35)	Sociodemographic, Diseases and Physical Measurement, Health Behaviour, Attitude and Social Environment
Vaillant & Western, 2001[53]	0-30 pack-years, ≥ 30 pack-years	OR, 95% CI<br>Happy-Well vs Sad-Sick and Prematurely Dead<br>Univariate Model: Smoking < 30 pack-years: 5.08 (2.85, 9.0)<br>Multivariate Model: Smoking < 30 pack-years: 5.00 (2.38, 10.5)	Alcohol Abuse Absent (Yes, No)	OR, 95%CI<br>Happy-Well vs Sad-Sick and Prematurely Dead<br>Univariate Model: No Alcohol abuse: 2.64 (1.45, 4.81)<br>Multivariate Model: No Alcohol abuse: 0.94 (0.72, 2.05).	Univariate: Diseases and Physical Measurements<br>Multivariate: Sociodemographic, Economic, Health Behaviour, Diseases and Physical Measurement, Attitude and Social Environment

For peer review only
For peer review only - http://bmjopen.bmj.com/site/about/guidelines.xhtml

Willcox et al., 2006[54]	Ever smoker, Never smoker	OR, 95%CI<br>Usual Survival vs Exceptional Survival<br>Age-adj. : 1.27 (1.06-1.53),<br>Fully-adj. : 1.23 (1.01-1.50)<br>Never Smokers: Ref	3 or more drinks/day, less than 3 drinks/day	OR, 95%CI<br>Usual Survival vs Exceptional Survival<br>Age-adj. : 1.84 (1.29-2.62) ,<br>Fully-adj.: 1.61 (1.11-2.34)<br>Less than 3 drinks/d: Ref	Sociodemographic

For peer review only

For peer review only - http://bmjopen.bmj.com/site/about/guidelines.xhtml

Table 3: Results of meta-analysis-Healthy Ageing compared to Smoking and Alcohol Consumption

Analysis	Number of Studies	ORs and 95%CI	p-value	I^2	Egger Bias; p-value	Trim-and-fill ORs and 95%CI	Number of Filled Studies
Never vs Current Smokers	7*	2.36 (2.03-2.75)	<.001	43.3%	1.42; .060	2.11 (1.82-2.45)	10
Never vs Former Smokers	5†	1.32 (1.23-1.41)	<.001	32.8%	-0.09; .935	unchanged	-
Past/Never vs Current	6‡	1.72 (1.20-2.47)	.003	87.2%	3.55; .142	1.20 (0.83-1.73)	9
Never vs Past/Current	5§	1.29 (1.16-1.43)	<.001	0.0%	1.08; .157	1.27 (1.14-1.40)	9
Drinkers vs Non-Drinkers	5‖	1.28 (1.08-1.52)	.004	72.1%	-1.25; .350	unchanged	-
Light vs Non-Drinkers	3¶	1.12 (1.03-1.22)	.010	0.0%	-8.17; .043	unchanged	-
Moderate vs Non- Drinkers	4**	1.35 (0.93-1.97)	.112	71.4%	1.77; .549	1.10 (0.77-1.57)	6
High to Non-Drinkers	3††	1.25 (1.09-1.44)	.002	0.0%	-0.63; .828	unchanged	-

OR: Odds Ratio; CI: Confidence Interval

* Bell et al., 2014[28]; Britton et al., 2008 (Males)[29]; Britton et al., 2008 (Females)[29]; Hodge, English et al., 2013[36]; Hodge, O'Dea et al., 2014[37]; LaCroix et al., 2016 (veterans)[39]; LaCroix et al., 2016 (non-veterans)[39]

† Bell et al., 2014[28]; Hodge, English et al., 2013[36]; Hodge, O'Dea et al., 2014[37]; LaCroix et al., 2016 (veterans)[39]; LaCroix et al., 2016 (non-veterans)[39]

‡ Ford et al., 2000[31]; Gu et al., 2009[32]; Guralnik & Kaplan, 1989[33]; Hamer et al., 2013[35]; Pruchno & Wilson-Genderson, 2015[44]; Terry et al., 2005[51]

§ Kaplan et al., 2008[38]; Newson et al., 2010[43]; Gureje et al., 2014[34]; Sabia et al., 2012[46]; Willcox et al., 2006[54]

|| Ford et al., 2000[31]; Gu et al, 2009[32]; Gureje et al., 2014[34]; LaCroix et al., 2016 (veterans)[39]; LaCroix et al., 2016 (non-veterans)[39]

¶ Hodge, English et al., 2013[36]; Hodge, O'Dea et al., 2014[37]; Sun et al., 2011[49]

** Britton et al., 2008 (Males)[29]; Britton et al., 2008 (Females)[29]; Guralnik & Kaplan, 1989[33]; Sun et al., 2011[49]

†† Hodge, English et al., 2013[36]; Hodge, O'Dea et al., 2014[37]; Sun et al., 2011[49]

For peer review only

List of figure legends

Figure 1: Selection Process Revised

Figure 2: Areas of information included in the definition of healthy ageing

Figure 3: Smoking and Healthy Ageing Forest Plot

Figure 4: Alcohol and Healthy Ageing Forest Plot

For peer review only

For peer review only - http://bmjopen.bmj.com/site/about/guidelines.xhtml

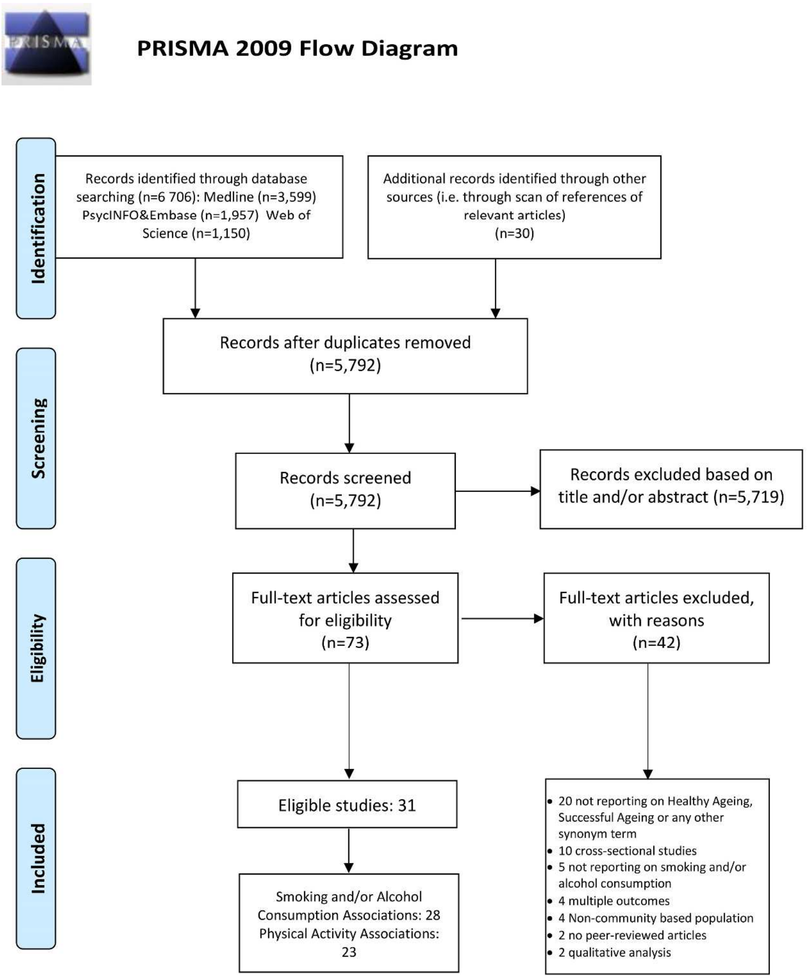

From: Moher D, Liberati A, Tetzlaff J, Altman DG, The PRISMA Group (2009). *P*referred *R*eporting *I*tems for *S*ystematic Reviews and *M*eta-*A*nalyses: The PRISMA Statement. PLoS Med 6(7): e1000097. doi:10.1371/journal.pmed1000097

For more information, visit www.prisma-statement.org.

Figure 1: Selection process-PRISMA flow diagram

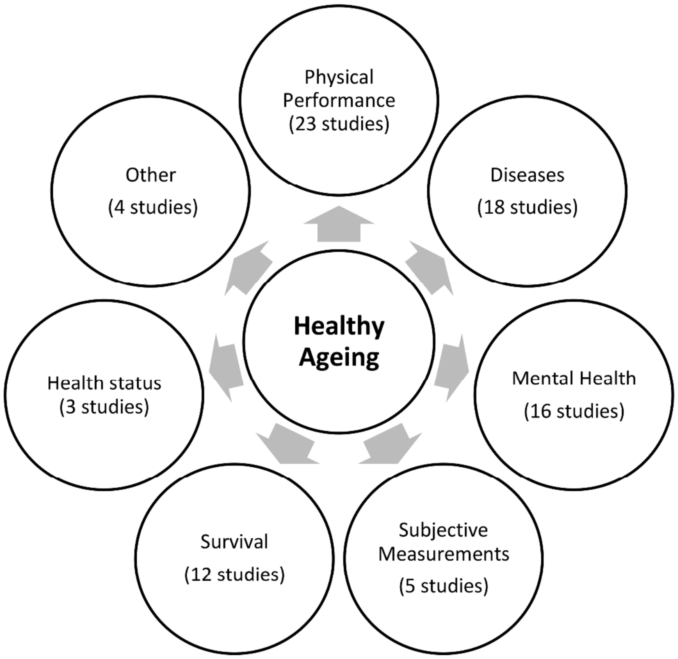

Figure 2: Areas of information included in the definition of healthy ageing

For peer review only - http://bmjopen.bmj.com/site/about/guidelines.xhtml

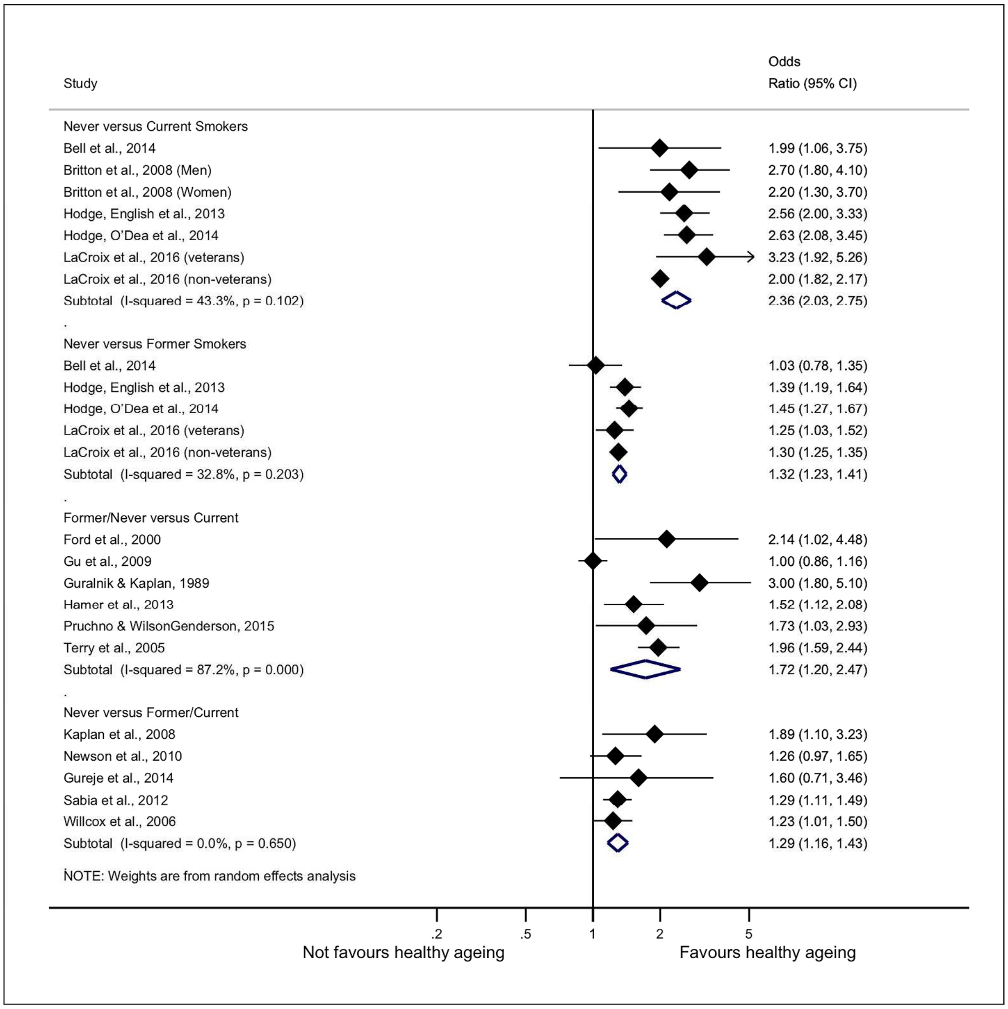

Figure 3: Smoking and healthy ageing forest plot

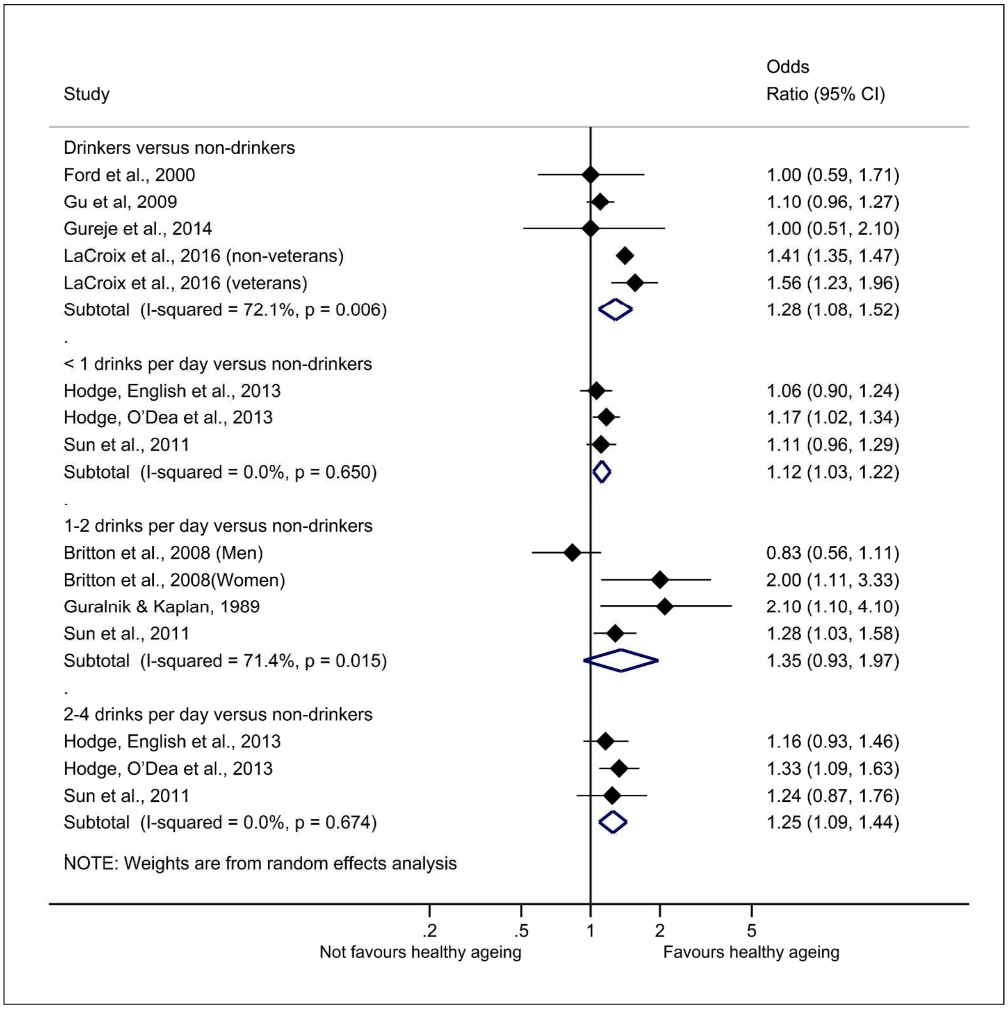

Figure 4: Alcohol and healthy ageing forest plot

Table A1: Areas of information included in the definition of healthy ageing

Authors	Survival	Health Status	Physical Performance	Diseases	Mental Health	Subjective Measurements	Others
Andrews et al., 2002			x		x		
Bell et al., 2014	x		x	x	x		
Britton et al., 2008		x	x	x	x		
Burke et al., 2001				x			
Ford et al., 2000							x
Gu et al., 2009	x		x				
Guralnik & Kaplan, 1989			x				
Gureje et al., 2014			x	x		x	
Hamer et al., 2013			x	x	x		
Hodge, English et al., 2013	x		x	x	x		
Hodge, O'Dea et al., 2014	x		x	x	x		
Kaplan et al., 2008		x	x				
LaCroix et al., 2016	x		x	x			
Li et al., 2001			x		x	x	
Liu & Su, 2016			x	x	x		
Newman et al., 2003			x	x	x		
Newson et al., 2010	x			x			
Pruchno & Wilson-Genderson, 2015			x	x		x	
Reed et al., 1998	x		x	x	x		
Sabia et al., 2012			x	x	x		
Sarnak et al., 2008			x	x	x		
Shields & Martel, 2006		x	x		x		
Sun et al., 2011	x		x	x	x		
Tampubolon, 2016							x
Terry et al., 2005	x			x	x		
Vaillant & Mukamal, 2001	x		x			x	x
Vaillant & Western, 2001	x		x			x	x
Willcox et al., 2006	x		x	x	x		
**Areas of information included in the definition of Healthy Ageing**	**12**	**3**	**23**	**18**	**16**	**5**	**4**

For peer review only

Table A2: Quality assessment of the studies – aggregated results of QUIPS tool

Author, Year of Publication	The study sample represents the population of interest on key characteristics, sufficient to limit potential bias of the observed relationship between PF and outcome.	Loss to follow-up is not associated with key characteristics sufficient to limit potential bias to the observed relationship between PF and outcome.	PF is adequately measured in study participants to sufficiently limit potential bias.	Outcome of interest is adequately measured in study participants to sufficiently limit potential bias.	Important potential confounders are appropriately accounted for, limiting potential bias with respect to the relationship between PF and outcome.	The statistical analysis is appropriate for the design of the study, limiting potential for presentation of invalid or spurious results.	Total
Andrews et al., 2002	Low Bias	Moderate Bias	Moderate Bias	Low Bias	Moderate Bias	Low Bias	Moderate Bias
Bell et al., 2014	Low Bias	Low Bias	Low Bias	Low Bias	Moderate Bias	Low Bias	Low Bias
Britton et al., 2008	Low Bias	Moderate Bias	Low Bias	Low Bias	Moderate Bias	Low Bias	Low Bias
Burke et al., 2001	Low Bias	Low Bias	Low Bias	Moderate Bias	Moderate Bias	Low Bias	Low Bias
Ford et al., 2000	Low Bias	Moderate Bias	Moderate Bias	Moderate Bias	Low Bias	Low Bias	Moderate Bias
Gu et al., 2009	Low Bias	Low Bias	Low Bias	Low Bias	Moderate Bias	Low Bias	Low Bias
Guralnik & Kaplan, 1989	Low Bias	Moderate Bias	Moderate Bias	Moderate Bias	Moderate Bias	Low Bias	Moderate Bias
Gureje et al., 2014	Low Bias	Moderate Bias	Low Bias	Low Bias	Moderate Bias	Low Bias	Low Bias
Hamer et al., 2013	Low Bias	Low Bias	Moderate Bias	Low Bias	Low Bias	Low Bias	Low Bias
Hodge, English et al., 2013	Low Bias	Moderate Bias	Low Bias	Low Bias	Low Bias	Low Bias	Low Bias
Hodge, O'Dea et al., 2014	Low Bias	High Bias	Low Bias	Low Bias	Low Bias	Low Bias	Moderate Bias
Kaplan et al., 2008	Low Bias	Moderate Bias	Low Bias	Moderate Bias	Low Bias	Low Bias	Low Bias
LaCroix et al., 2016	Low Bias	Moderate Bias	Low Bias	Low Bias	Moderate Bias	Low Bias	Low Bias
Li et al., 2001	Low Bias	High Bias	Low Bias	Low Bias	Moderate Bias	Low Bias	Moderate Bias
Liu & Su, 2016	Low Bias	Moderate Bias	Low Bias	Low Bias	Moderate Bias	Low Bias	Low Bias
Newman et al., 2003	Low Bias	Low Bias	Low Bias	Low Bias	Moderate Bias	Low Bias	Low Bias
Newson et al.,	Low Bias	Moderate Bias	Low Bias	Low Bias	Moderate Bias	Low Bias	Low Bias

For peer review only

2010							
Pruchno & Wilson-Genderson, 2015	Low Bias	Moderate Bias	Low Bias	Low Bias	Moderate Bias	Low Bias	Low Bias
Reed et al., 2011	Low Bias	Moderate Bias	Low Bias	Low Bias	Low Bias	Moderate Bias	Low Bias
Sabia et al., 2012	Low Bias	Low Bias	Low Bias	Moderate Bias	Low Bias	Low Bias	Low Bias
Sarnak et al., 2008	Low Bias	Moderate Bias	Moderate Bias	Low Bias	Moderate Bias	Low Bias	Moderate Bias
Shields & Martel, 2006	Low Bias	Moderate Bias	Low Bias	Low Bias	Low Bias	Low Bias	Low Bias
Sun et al., 2011	Low Bias	Low Bias	Low Bias	Low Bias	Low Bias	Low Bias	Low Bias
Tampubolon, 2016	Low Bias	Low Bias	Low Bias	Low Bias	Moderate Bias	Low Bias	Low Bias
Terry et al., 2005	Low Bias	Low Bias	Low Bias	Moderate Bias	Moderate Bias	Moderate Bias	Moderate Bias
Vaillant & Mukamal, 2001	High Bias	Moderate Bias	Low Bias	Moderate Bias	Moderate Bias	Low Bias	High Bias
Vaillant & Western, 2001	High Bias	Low Bias	Low Bias	Low Bias	Moderate Bias	Low Bias	Moderate Bias
Willcox et al., 2006	Moderate Bias	Moderate Bias	Low Bias	Low Bias	Moderate Bias	Low Bias	Moderate Bias

For peer review only

Table A3: Sensitivity analysis of meta-analytic results

Analysis	Number of Studies	DerSimonian-Laird ORs and 95%CI	p-value	I^2	Paule-Mandel ORs and 95%CI	p-value	I^2
Never vs Current Smokers	7*	2.36 (2.03-2.75)	<.001	43.3%	2.29 (2.03-2.59)	<.001	23.6%
Never vs Former Smokers	5†	1.32 (1.23-1.41)	<.001	32.8%	1.31 (1.21-1.43)	<.001	50.0%
Past/Never vs Current	6‡	1.72 (1.20-2.47)	.003	87.2%	1.69 (1.25-2.29)	<.001	81.3%
Never vs Past/Current	5§	1.29 (1.16-1.43)	<.001	0.0%	1.29 (1.16-1.43)	<.001	0.0%
Drinkers vs Non-Drinkers	5‖	1.28 (1.08-1.52)	.004	72.1%	1.29 (1.10-1.50)	.002	68.2%
Light vs Non-Drinkers	3¶	1.12 (1.03-1.22)	.010	0.0%	1.12 (1.03-1.22)	.010	0.0%
Moderate vs Non-Drinkers	4**	1.35 (0.93-1.97)	.112	71.4%	1.37 (0.90-2.08)	.138	77.0%
High to Non-Drinkers	3††	1.25 (1.09-1.44)	.002	0.0%	1.25 (1.09-1.44)	.002	0.0%

OR: Odds Ratio; CI: Confidence Interval

* Bell et al., 2014; Britton et al., 2008 (Males); Britton et al., 2008 (Females); Hodge, English et al., 2013; Hodge, O'Dea et al., 2014; LaCroix et al., 2016 (veterans); LaCroix et al., 2016 (non-veterans)

† Bell et al., 2014; Hodge, English et al., 2013; Hodge, O'Dea et al., 2014; LaCroix et al., 2016 (veterans); LaCroix et al., 2016 (non-veterans)

‡ Ford et al., 2000; Gu et al., 2009; Guralnik & Kaplan, 1989; Hamer et al., 2013; Pruchno & Wilson-Genderson, 2015; Terry et al., 2005

§ Kaplan et al., 2008; Newson et al., 2010; Gureje et al., 2014; Sabia et al., 2012; Willcox et al., 2006

‖ Ford et al., 2000; Gu et al, 2009; Gureje et al., 2014; LaCroix et al., 2016 (veterans); LaCroix et al., 2016 (non-veterans)

¶ Hodge, English et al., 2013; Hodge, O'Dea et al., 2014; Sun et al., 2011

** Britton et al., 2008 (Males); Britton et al., 2008 (Females); Guralnik & Kaplan, 1989; Sun et al., 2011

†† Hodge, English et al., 2013; Hodge, O'Dea et al., 2014; Sun et al., 2011

For peer review only

Figure A1: Smoking and healthy ageing-age stratified

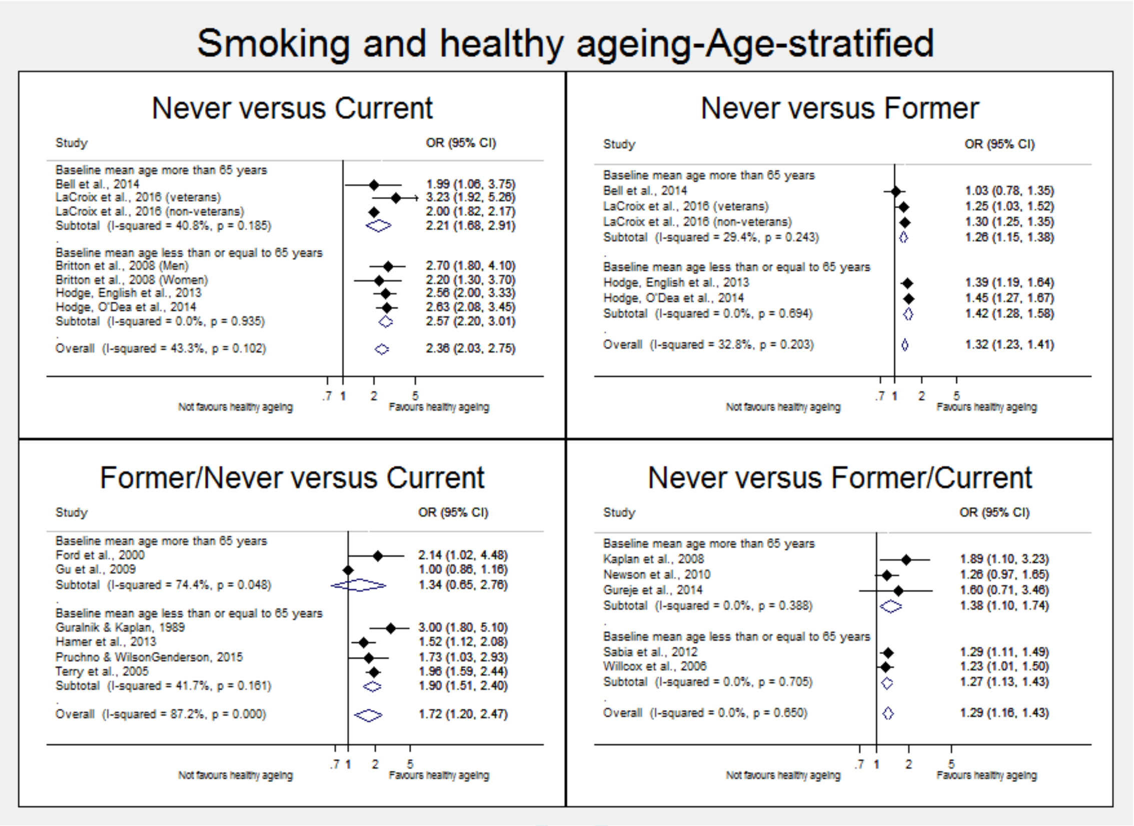

Figure A2: Smoking and healthy ageing-follow-up time stratified

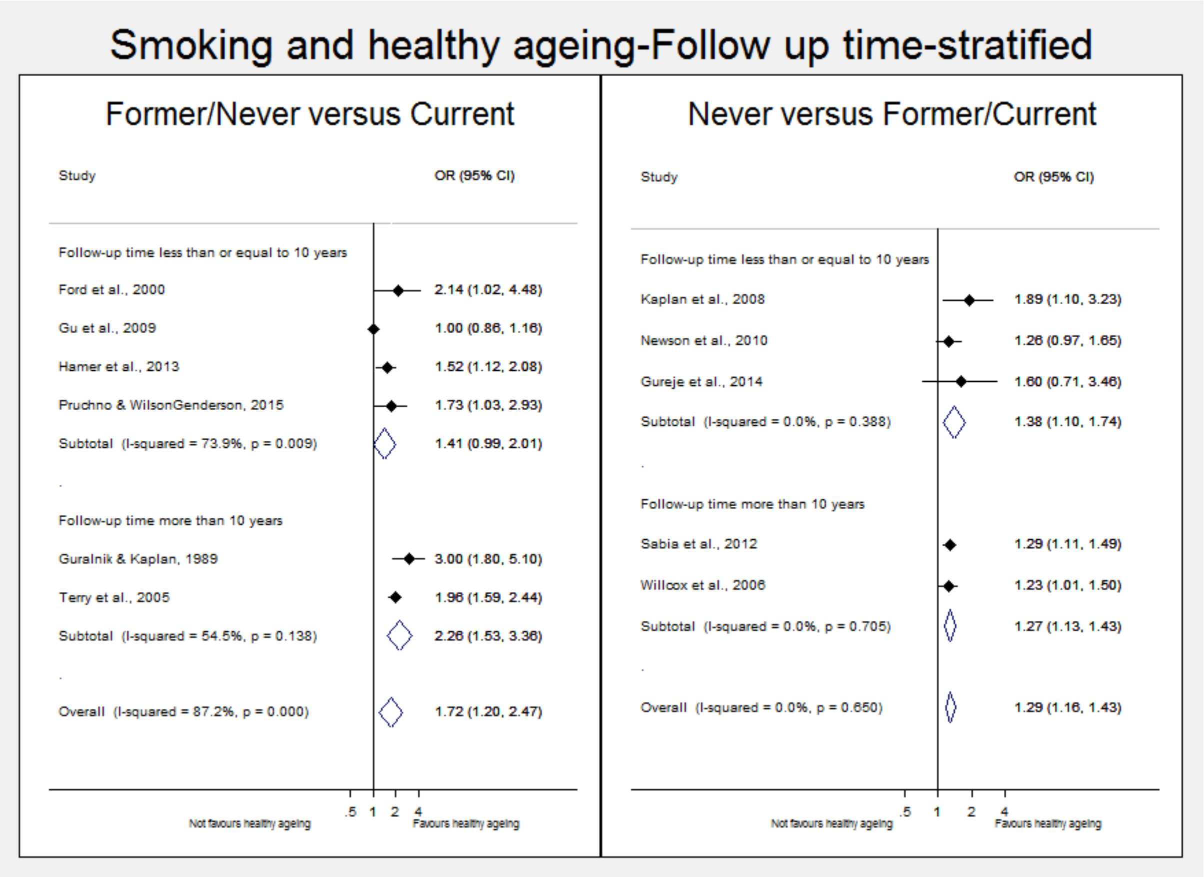

Figure A3: Alcohol and healthy ageing-follow-up stratified

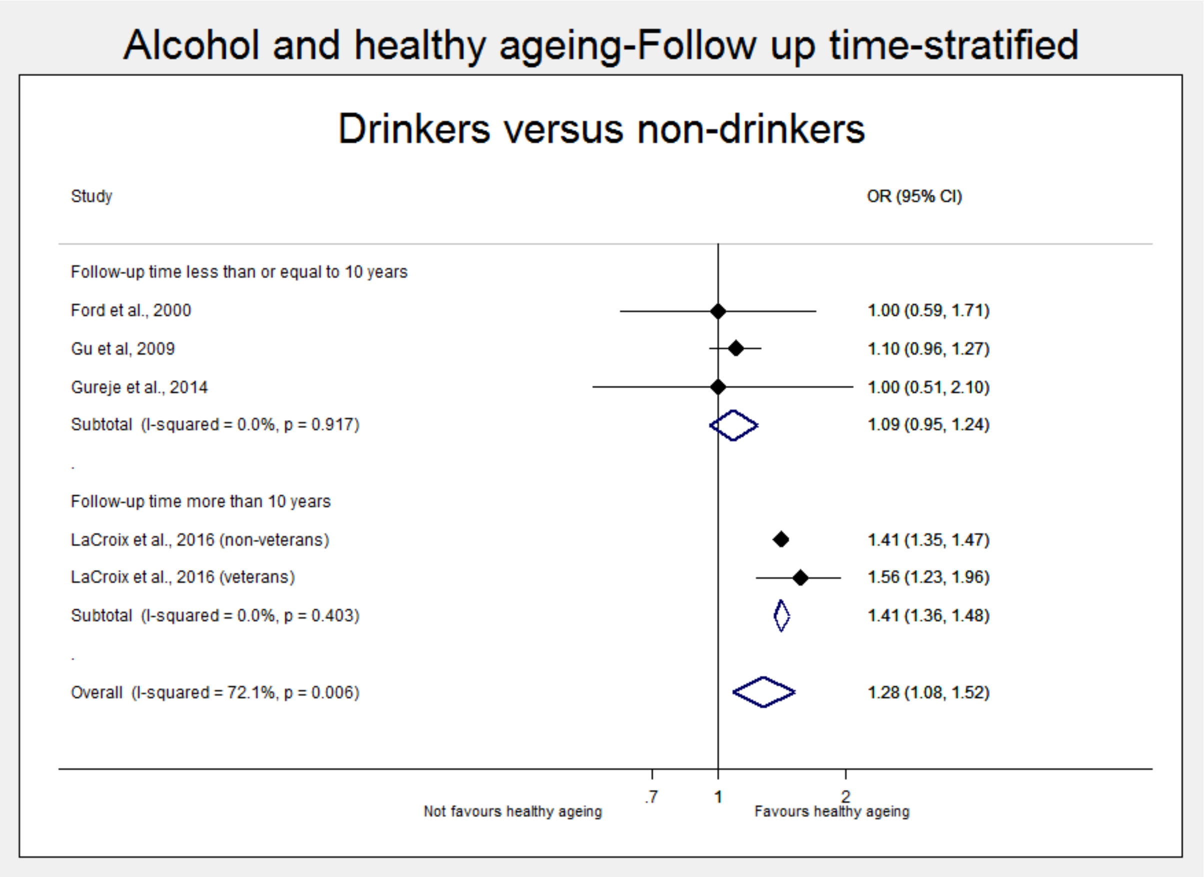

For peer review only

MOOSE Checklist for Meta-analyses of Observational Studies

Item No	Recommendation	Reported on Page No
Reporting of background should include		
1	Problem definition	To address the need for more accurate estimates of the relationship between smoking and healthy ageing and alcohol consumption and healthy ageing (p. 6)
2	Hypothesis statement	In systematic reviews focusing on elderly people, smoking is associated with premature mortality and an increased incidence of Alzheimer's disease. Studies showed that light to moderate alcohol consumption is associated with a decrease in dementia and cognitive incidence, as well as with a reduced risk for cardiovascular disease mortality, incident coronary heart disease, incident stroke, stroke mortality and all-cause mortality(p. 5)
3	Description of study outcome(s)	The primary outcome of this review was health status measured by healthy ageing, and any other term related to it (e.g. successful ageing, active ageing, healthy survival etc.) (p. 7)
4	Type of exposure or intervention used	Eligible studies had to meet the following criteria: i. being published in an electronic journal article; ii. constitute an original peer-reviewed longitudinal study; and iii. report any kind of longitudinal association between smoking and/or alcohol consumption and healthy ageing (p. 7)
5	Type of study designs used	longitudinal study (p. 7)
6	Study population	Studies that included cohorts of individuals who were institutionalised or hospitalised and animal studies were also excluded (p. 7)
Reporting of search strategy should include		
7	Qualifications of searchers (eg, librarians and investigators)	Credentials are indicated in the title page
8	Search strategy, including time period included in the synthesis and key words	Search strategy and selection criteria (p.6-7) and Appendix 3.docx
9	Effort to include all available studies, including contact with authors	In case that full text could not be retrieved, the corresponding author of the paper was contacted (p. 7).<br>Other relevant systematic reviews of healthy ageing and reference lists of the eligible studies were also searched (p.7)
10	Databases and registries searched	MEDLINE (PubMed interface), EMBASE (OVID interface), Psychinfo (OVID interface) and CENTRAL (Cochrane Central Register of Controlled Trials) (p. 6-7)
11	Search software used, name and version, including special features used (eg, explosion)	EndNote (ENDNOTE X7, Thomson Reuters), (p. 7)
12	Use of hand searching (eg, reference lists of obtained articles)	Other relevant systematic reviews of healthy ageing and reference lists of the eligible studies have also been searched (p. 7)
13	List of citations located and those excluded, including justification	Details of the literature search are outlined in the PRISMA flow-chart: Figure 1. The citation list is available upon request.
14	Method of addressing articles published in languages other than English	Acknowledgements (p. 20)
15	Method of handling abstracts and unpublished studies	exclusion of papers that were abstracts, conference papers, cross-sectional studies (p. 9-10)
16	Description of any contact with authors	we planned to contact authors for any missing data; however, this was not needed.

For peer review only

Reporting of methods should include		
17	Description of relevance or appropriateness of studies assembled for assessing the hypothesis to be tested	Search strategy and selection criteria paragraph (p. 6-7)
18	Rationale for the selection and coding of data (eg, sound clinical principles or convenience)	Data extraction and quality assessment paragraph (p. 8) and Table 1 and Table 2. We extracted data from each study that were relevant to the population, the exposure, the outcome and any confounder.
19	Documentation of how data were classified and coded (eg, multiple raters, blinding and interrater reliability)	Data extraction and quality assessment paragraph (p. 8).
20	Assessment of confounding (eg, comparability of cases and controls in studies where appropriate)	We extracted information regarding the different confounders that were used per study (Table 2)
21	Assessment of study quality, including blinding of quality assessors, stratification or regression on possible predictors of study results	Two investigators assessed study quality by using the QUIPS tool (Quality Assessment paragraph, p.11 and Table A2)
22	Assessment of heterogeneity	Heterogeneity was assessed by the I^2 (Data Analysis paragraph, p. 9)
23	Description of statistical methods (eg, complete description of fixed or random effects models, justification of whether the chosen models account for predictors of study results, dose-response models, or cumulative meta-analysis) in sufficient detail to be replicated	random effects meta-analysis was used (p. 9)
24	Provision of appropriate tables and graphics	We provided the PRISMA flow-chart, tables with the characteristics of studies, table with the results of meta-analysis and forest plots
Reporting of results should include		
25	Graphic summarizing individual study estimates and overall estimate	Figure 2 and Figure 3
26	Table giving descriptive information for each study included	Table 1 and Table 2
27	Results of sensitivity testing (eg, subgroup analysis)	Subgroup analysis (page 12-13)
28	Indication of statistical uncertainty of findings	95% confidence intervals were presented with all estimators, I^2 values, and results of the trim-and-fill algorithm. Table 3.

Item No	Recommendation	Reported on Page No
Reporting of discussion should include		
29	Quantitative assessment of bias (eg, publication bias)	Publication bias was assessed Egger test and the trim-and-fill algorithm (Table 3)
30	Justification for exclusion (eg, exclusion of non-English language citations)	We did not exclude any study due to language. We excluded studies for the meta-analysis because they did not report a relevant statistic to OR or because alcohol conversion to comparable metrics was not possible
31	Assessment of quality of included studies	Data extraction and quality assessment paragraph (p. 8) and Table A2.
Reporting of conclusions should include		
32	Consideration of alternative explanations for observed results	Limitations are discussed in parag. Strengths and Limitations (p. 17). The most important are: adjustment by different use of covariates, different follow-up time and attrition rate, self-reported questionnaires and inconsistency in the way smoking/alcohol have been measured among the different cohorts.
33	Generalization of the conclusions (ie, appropriate for the data presented and within the domain of the literature review)	The positive impact of smoking abstinence and smoking cessation on healthy ageing is identified in both the majority of our primary studies and in our pooled effect estimate as well (even when we adjusted for publication bias). A positive relationship between limited

For peer review only - http://bmjopen.bmj.com/site/about/guidelines.xhtml

		consumption of drinking could also be argued but more research is needed.
34	Guidelines for future research	Conclusion parag. (p. 19)
35	Disclosure of funding source	Funding parag. (p.20-21)

From: Stroup DF, Berlin JA, Morton SC, et al, for the Meta-analysis Of Observational Studies in Epidemiology (MOOSE) Group. Meta-analysis of Observational Studies in Epidemiology. A Proposal for Reporting. *JAMA*. 2000;283(15):2008-2012. doi: 10.1001/jama.283.15.2008.
Transcribed from the original paper within the NEUROSURGERY® Editorial Office, Atlanta, GA, United Sates. August 2012.

For peer review only

UNIVERSITY of York
Centre for Reviews and Dissemination

NHS
National Institute for Health Research

PROSPERO International prospective register of systematic reviews

ATHLOS: impact of physical activity, smoking and alcohol on healthy ageing throughout the lifespan: protocol for a systematic review

Christina Daskalopoulou, Matthew Prina, Martin Prince, Artemis Koukounari

Citation

Christina Daskalopoulou, Matthew Prina, Martin Prince, Artemis Koukounari. ATHLOS: impact of physical activity, smoking and alcohol on healthy ageing throughout the lifespan: protocol for a systematic review. PROSPERO 2016:CRD42016038130 Available from http://www.crd.york.ac.uk/PROSPERO_REBRANDING/display_record.asp?ID=CRD42016038130

Review question(s)

What is the impact of physical activity, smoking and alcohol consumption across the life span on healthy ageing?

Quantification and summary of the relationship between the aforementioned lifestyle factors and healthy ageing by using a meta-analytical approach.

Searches

The literature search will include any related term or synonym of healthy ageing and text words related to physical activity, smoking and alcohol. Where possible, the latter will be developed using medical subject headings (MeSH). We will search MEDLINE (PubMed interface), EMBASE (OVID interface), PsycINFO (OVID interface), Web of Science and CENTRAL (Cochrane Central Register of Controlled Trials).

In addition, scanning of the reference lists of the included studies will take place so as to identify any relevant studies that were not included by the initial research. Finally, authors of relevant studies will be contacted in case that supplementary information is required.

Types of study to be included

This review will be restricted to original studies (peer-reviewed).

Opinion pieces, books, letters, editorials, abstracts, dissertations/thesis and/or conference proceedings will not be considered.

Condition or domain being studied

Health status measured by healthy ageing.

Participants/ population

Eligible studies will include people, who are community-based, across the whole life span. Hospitalised or institutionalised populations will not been considered. Participants being disabled or diagnosed with dementia, frailty or any chronic disease will be included in the systematic review as long as they are community-based. There will be no other restrictions, for instance gender, age, education level, occupation and/or ethnicity.

Intervention(s), exposure(s)

Associations of either physical activity, smoking and/or alcohol consumption to one of the outcomes of healthy ageing, irrespectively of the way the latter is being measured/defined.

Comparator(s)/ control

Not applicable.

Context

Community-based populations and not clinical ones.

For peer review only - http://bmjopen.bmj.com/site/about/guidelines.xhtml

UNIVERSITY of York
Centre for Reviews and Dissemination

NHS
National Institute for Health Research

Outcome(s)

Primary outcomes

Health status measured by healthy ageing.

Secondary outcomes

Not applicable.

Data extraction, (selection and coding)

An EndNote (ENDNOTE X7, Thomson Reuters) library will be created so as to store all the studies retrieved by the electronic databases. Using EndNote's auto-deduplication function, duplicate citations will be removed. Since auto-deduplication is thought to be only partially successful (Qi et al., 2013), the remaining duplicates will be identified by hand-searching techniques. To do this, references will be alphabetically ordered according to the first authors' names and thereafter according to their titles.

Studies from the electronic database searches will be initially determined as relevant or not by title and abstract screening. References will be characterised as "Not Eligible" and "Potentially Eligible" by screening the title and the abstract of the study.

In order to include a study in the "Potential Eligible" folder the following criteria will be applied:

Study characteristics: "potentially eligible studies" have to be published in an electronic journal article and constitute an original peer-reviewed longitudinal study. We will include studies that examined healthy ageing as an outcome and that reported on the following determinants: physical activity, smoking and/or alcohol consumption.

Study participants: to be "potentially eligible", the title or abstract of the study must clearly state that the study population is an observational study/cohort study and that people are not hospitalised or belong to a specific clinical population. Participants being disabled or diagnosed with dementia, frailty or any chronic disease will be included in the systematic review as long as they are community based. There will be no other restrictions.

Interventions: To be "potentially eligible", the title or abstract must clearly state that the study reports the associations of either physical activity, smoking and/or alcohol consumption to one of the outcomes of healthy ageing, irrespectively of the way the latter is being measured/defined.

The final inclusion will be based on full text screening. Full text studies of "potentially eligible" references will be retrieved and assessed for their final inclusion by the first reviewer and a second researcher. A random sample of those, accounting for the 5% of the total studies, will be checked by a senior epidemiologist. Once the screening process is completed, the creation of a PRISMA flow diagram will be generated so as to illustrate the study selection process.

Risk of bias (quality) assessment

The risk of bias in primary studies will be assessed by using the Quality in Prognosis Studies (QUIPS) tool (Hayden et al., 2013). Validity and bias should be assessed by considering the following 6 areas: participation, attrition, prognostic factor measurement, confounding measurement and account, outcome measurement and analysis and reporting. During the first step, the 6 aforementioned potential sources of bias will be classified as either "yes", "partly", "no" or "unsure" by the first reviewer. Each study will then be characterised with an overall risk bias rating of "high", "moderate" or "low".

This characterisation will be defined as follows: "low" risk of bias will have these studies which fulfil all or the majority of the 6 QUIPS tool domains (no source of bias should be characterised as "yes" and at most one "partly"). "Moderate" risk of bias will have the studies that fullfil some of the aforementioned criteria; and "high" risk of bias will have those that meet few or none of the criteria.

During the next step, the first reviewer and the senior epidemiologist will judge if any of the "high" and "moderate" level studies present fatal flaws or considerable amount of bias and hence, in order to avoid reaching any misleading conclusion, it would be better to exclude these studies from the results (Sterne et al., 2001). The reasons based on which any studies have been excluded will be provided with full detail in a supplementary table.

For peer review only - http://bmjopen.bmj.com/site/about/guidelines.xhtml

Publication bias will be assessed for syntheses including five or more studies using a funnel plot and the Egger regression asymmetry test.

Strategy for data synthesis

The impact of physical activity, smoking and alcohol consumption on healthy ageing across the lifespan will be presented. In particular, the different levels of these factors and how these are related to healthy ageing will be extracted.

We will consider the different levels/measurements of these factors as statistically significant if i) the reported P-value is less than 0.05; ii) the author reported that there was a significant association; and iii) the 95% confidence interval around odds ratio or a similar statistic did not include 1.

Studies that refer no impact of the aforementioned determinants will also be presented. In addition, any concerns regarding the generalisability of the findings will be mentioned. For example, a small sample size, ethnicity/gender bias or important number of participants that were lost to follow-up will be referred.

Assuming non-substantial heterogeneity in study characteristics (e.g. determinant measurements, outcomes, etc.), we will carry out a meta-analysis to pool together effect estimates for each determinant. I-squared statistic will be used in order to assess the heterogeneity of the studies and based on this and the theoretical ground of the included studies, a fixed or random effect meta-analysis will be performed (Higgins et al., 2011). If a meta-analysis is not feasible due to amplified heterogeneity for the outcome and/or the lifestyle determinant measurement, a narrative synthesis for summarising the evidence will be performed.

In case of any discrepancies among the studies, study designs, populations, precision and publication bias will be assessed. Quality of our evidence will be considered as high when further research is very unlikely to change our confidence in the estimate of effect, moderate when further research is likely to have a considerable impact on our confidence and may change our estimate. The quality of evidence will be considered as low, in case that further research is very likely to have an important impact on our confidence in the estimate of effect, and is likely to change the estimate; and very low, when we are not certain about the estimate effect.

Quality of our findings will be assessed by performing a sensitivity analysis, should the selected studies permit it. For example, we will repeat the meta-analysis by excluding studies with low quality and see what will be the impact of it to our conclusions.

Analysis of subgroups or subsets

If the selected data permit, sub-group analyses will be carried out so as to stratify for different physical activity measurements (i.e subjective/self-report vs objective assessment of PA).

Dissemination plans

The findings of this review will be presented to relevant scientific conferences, for example ATHLOS consortium workshops, as well as to academic journals. Furthermore, conclusions will be disseminated through research and university networks.

Contact details for further information

Christina Daskalopoulou

King's College London, Institute of Psychiatry, Psychology & Neuroscience, Centre for Global Mental Health, Department of Health Services and Population Research, David Goldberg Centre, De Crespigny Park, Denmark Hill, London SE5 8AF, UK.

christina.daskalopoulou@kcl.ac.uk

Organisational affiliation of the review

King's College London, Institute of Psychiatry, Psychology & Neuroscience, Centre for Global Mental Health, Department of Health Services and Population Research

For peer review only - http://bmjopen.bmj.com/site/about/guidelines.xhtml

UNIVERSITY of York
Centre for Reviews and Dissemination
NHS
National Institute for Health Research

http://www.kcl.ac.uk/ioppn/depts/hspr/index.aspx

Review team

Ms Christina Daskalopoulou, King's College London
Dr Matthew Prina, King's College London
Professor Martin Prince, King's College London
Dr Artemis Koukounari, King's College London

Collaborators

Dr Carolina Kralj, King's College London

Anticipated or actual start date

14 March 2016

Anticipated completion date

31 December 2016

Funding sources/sponsors

Department of Health Services and Population Research, Institute of Psychology, Psychiatry and Neuroscience at King's College London is the Sponsor, meaning that it has overall control of the data. This project falls under the ATHLOS (Ageing Trajectories of Health: Longitudinal Opportunities and Synergies) project, funded by the European Union's Horizon 2020 Research and Innovation Programme under grant agreement number 63531.

Conflicts of interest

None known

Language

English

Country

England

Subject index terms status

Subject indexing assigned by CRD

Subject index terms

Age Factors; Aging; Alcohol Drinking; Exercise; Health; Health Status; Humans; Motor Activity; Risk Factors; Smoking

Reference and/or URL for protocol

http://www.crd.york.ac.uk/PROSPEROFILES/38130_PROTOCOL_20160326.pdf

Stage of review

Ongoing

Date of registration in PROSPERO

26 April 2016

Date of publication of this revision

26 April 2016

Stage of review at time of this submission	Started	Completed
Preliminary searches	Yes	No
Piloting of the study selection process	Yes	No

For peer review only - http://bmjopen.bmj.com/site/about/guidelines.xhtml

UNIVERSITY of York
Centre for Reviews and Dissemination

NHS
National Institute for Health Research

Formal screening of search results against eligibility criteria	No	No
Data extraction	No	No
Risk of bias (quality) assessment	No	No
Data analysis	No	No

PROSPERO
International prospective register of systematic reviews
The information in this record has been provided by the named contact for this review. CRD has accepted this information in good faith and registered the review in PROSPERO. CRD bears no responsibility or liability for the content of this registration record, any associated files or external websites.

For peer review only

For peer review only - http://bmjopen.bmj.com/site/about/guidelines.xhtml

Systematic Review Protocol: ATHLOS: Impact of Physical Activity, Smoking and Alcohol on Healthy Ageing throughout the Life Span: protocol for a systematic review.

Embase, Psychinfo (OVID interface) and Cochrane Library

(health* ageing) OR (health* aging) OR (successful ageing) OR (successful aging) OR (positive ageing) OR (positive aging) OR (productive ageing) OR (productive aging) OR (ageing well) OR (aging well) OR (optim* ageing) OR (optim* aging) OR (unimpaired ageing) OR (unimpaired aging) OR (effective ageing) OR (effective aging) OR (robust ageing) OR (robust aging) OR (exceptional survival)

AND ((smoking) OR (tobacco) OR (cigarette*) OR (physical activity) OR (physical inactivity) OR (exercise*) OR (alcohol) OR (Alcohol Drinking))

MedLine search (PubMed/PMC)

(("health* ageing") OR ("health* aging") OR ("successful ageing") OR ("successful aging") OR ("positive ageing") OR ("positive aging") OR ("productive ageing") OR ("productive aging") OR ("ageing well") OR ("aging well") OR ("optim* ageing") OR ("optim* aging") OR ("unimpaired ageing") OR ("unimpaired aging") OR ("effective ageing") OR ("effective aging") OR ("robust ageing") OR ("robust aging") OR ("exceptional survival")) AND (("motor activity"[MeSH Terms]) OR ("smoking"[MeSH Terms]) OR ("Alcohol Drinking"[MesH Terms]))

For peer review only
